# Multiscale modes of functional brain connectivity

**DOI:** 10.1162/IMAG.a.1031

**Published:** 2025-12-10

**Authors:** S. Rezvan Farahibozorg, Samuel J. Harrison, Janine D. Bijsterbosch, Mark W. Woolrich, Stephen M. Smith

**Affiliations:** FMRIB, Oxford Centre for Integrative Neuroimaging, Nuffield Department of Clinical Neuroscience, Oxford University, Oxford, United Kingdom; Department of Radiology, Washington University in St Louis, St. Louis, MO, United States; OHBA, Oxford Centre for Integrative Neuroimaging, Department of Psychiatry, Oxford University, Oxford, United Kingdom

**Keywords:** multiscale modes, resting-state fMRI, individual-specific modelling, PROFUMO, trait prediction, big data

## Abstract

Information processing in the brain spans from localised sensorimotor processes to higher-level cognition that integrates across multiple regions. Interactions between and within these subsystems enable multiscale information processing. Despite this multiscale characteristic, functional brain connectivity is often either estimated based on 10–30 distributed modes or parcellations with 100–1000 localised parcels, both missing *across*-scale functional interactions. We present Multiscale Probabilistic Functional Modes (mPFMs), a new mapping which comprises modes over various scales of granularity, thus enabling direct estimation of functional connectivity within- and across-scales. Crucially, mPFMs were not formulated, but emerged from data-driven multilevel Bayesian modelling of large functional MRI (fMRI) populations and every individual. We demonstrate that mPFMs capture both distributed brain modes and their co-existing subcomponents. In addition to validating mPFMs using simulations and real data, we show that mPFMs can predict ~900 personalised traits from UK Biobank more accurately than current standard techniques. Therefore, mPFMs can offer a new basis for functional connectivity modelling and yield enhanced fMRI biomarkers for traits and diseases.

## 1 Introduction

Within the complex system of 86 billion neurons (Herculano-Houzel, 2009) in the human brain, large ensembles of neurons work in synchrony, such that they produce distinct modes of extended, correlated activity (Smith et al., 2009). These functional modes exhibit a spectrum of scales and functionalities, from localised brain modes facilitating specialised information processing, such as responses to sensory or motor stimuli, to higher-level cognitive modes integrating across multiple regions through long-range connections. Functional modes underlie spontaneous brain activity during task-free periods (often referred to as resting-state networks, RSNs), adapt dynamically during cognitive tasks, and undergo permanent changes in neurodegenerative diseases (Biswal et al., 1995; Buckner & Vincent, 2007; Calhoun et al., 2008; Damoiseaux et al., 2006; Raichle et al., 2001; Smith et al., 2013b). Growing evidence, especially from new datasets with thousands of individuals, has highlighted that spatiotemporal characteristics of functional modes vary systematically across individuals, providing biomarkers for traits and disease, akin to a diagnostic assay for the brain (Elliott et al., 2018; Finn et al., 2015; Jiang et al., 2020; Kong et al., 2019; Smith et al., 2015; Vidaurre et al., 2017). In this paper, we introduce a novel representation, Multiscale Probabilistic Functional Modes (mPFMs), which, unlike the existing single-scale modes, encapsulates an ensemble of modes across multiple scales. mPFMs have the potential to provide a shift in how functional connectivity has been modelled to date, by offering a new basis to gain insights into spatiotemporal connectivity within and across diverse processing scales, and providing more sensitive biomarkers from functional neuroimaging.

Functional MRI (fMRI), with a spatial resolution of ~2 mm and a temporal resolution of ~1 second, provides a suitable spatiotemporal resolution to characterise functional modes non-invasively. This has largely been done using two distinct approaches: (a) high-dimensional (highD) decomposition of fMRI data into functional parcellations with 100–1000 modes (i.e., parcels), where each mode is typically *localised* to a single brain region (Bellec et al., 2010; Craddock et al., 2012; Schaefer et al., 2018); (b) low-dimensional (lowD) decomposition of fMRI data into ~20–30 modes (Beckmann & Smith, 2004; Calhoun et al., 2001)—the conventional RSNs—each *distributed* across multiple distant brain regions, and often with some spatial overlap between the modes. The latter is often carried out using spatial Independent Component Analysis (ICA) (McKeown et al., 1998), or lowD parcellations (Yeo et al., 2011). Each of these two approaches offers distinct benefits. On the one hand, fine-grained, highD parcellations capture local details in the brain’s organisation, especially details that have proved useful for cross-individual variability modelling, and prediction of traits and disease (Dadi et al., 2020; Pervaiz et al., 2020). On the other hand, lowD large-scale modes capture the more global functional configuration of the brain. Distant brain regions with intrinsic long-range connections are grouped into biologically meaningful neural entities such as language and attention networks, allowing for easier interpretation and putative links to cognitive functions (Power et al., 2011; Smith et al., 2009; Yeo et al., 2011). Additionally, lower data dimensionality makes subsequent statistical analyses more manageable. Therefore, distributed versus localised modes each offers distinct benefits and limitations for capturing global versus local functional architecture of the brain. Crucially, however, neither of these approaches directly model inherent interactions across multiple scales of information processing in the brain.

In this context, we report the finding of mPFMs, which can bridge this gap by providing a unified representation that encompasses modes across multiple scales. mPFMs were identified through high-dimensional decomposition of resting-state fMRI using the sPROFUMO framework (Farahibozorg et al., 2021). sPROFUMO defines a Bayesian hierarchy with two levels of group and individuals across big data populations, where group priors are iteratively used for top-down regularisation of individuals, and evidence across individuals is accumulated and fed back to the group. Two key distinguishing aspects of the model are that (1) sPROFUMO is subject specific, that is, inferring the modes for populations and individuals *simultaneously*; (2) unlike ICA or parcellation-based techniques, sPROFUMO does not impose strong constraints on spatial or temporal independence between the modes, thus yielding modes that are more flexibly interacting, spatially and temporally (Farahibozorg et al., 2021; Harrison et al., 2020).

**Fig. 1.**
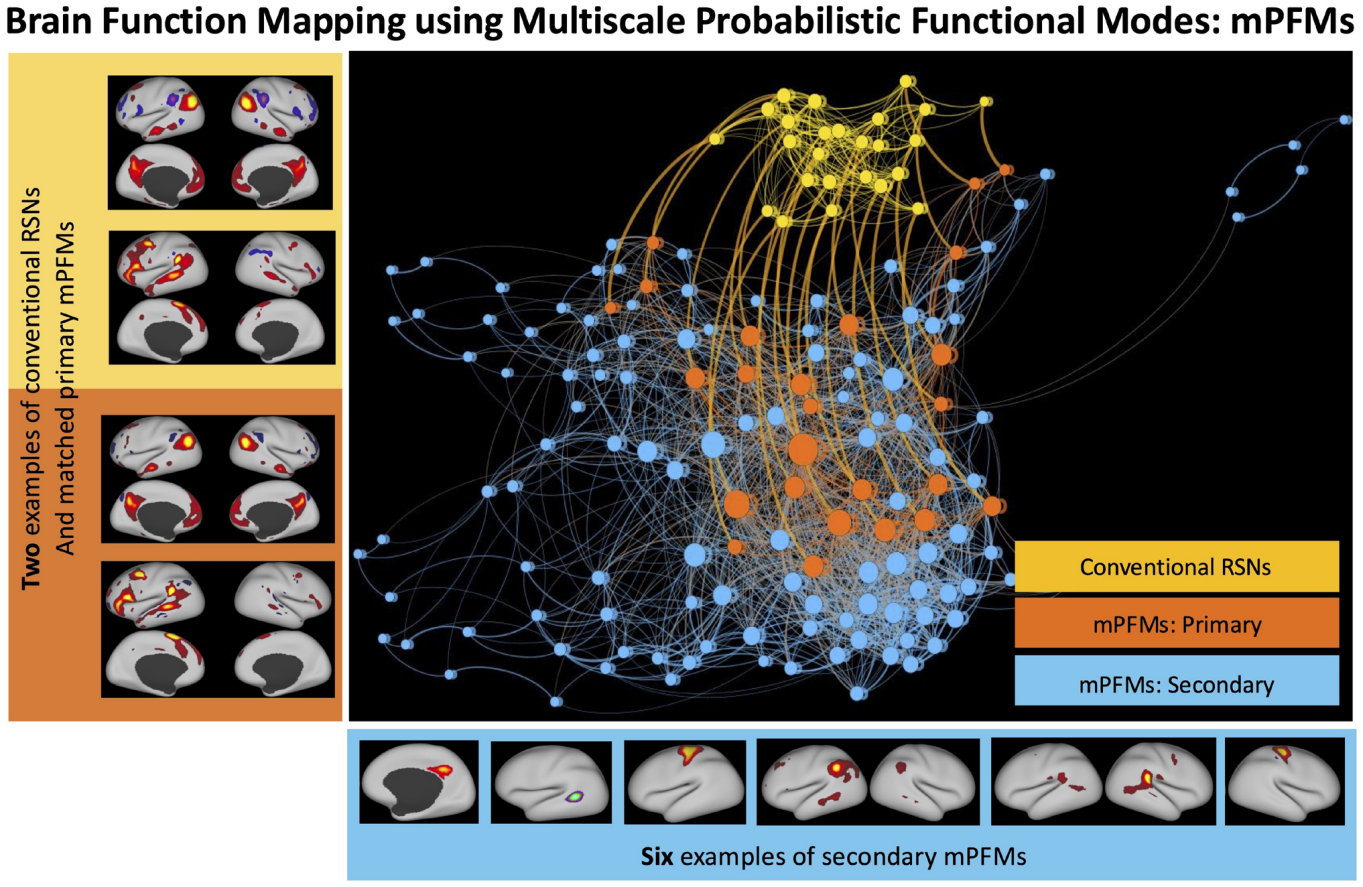
Brain function mapping using Multiscale Probabilistic Functional Modes (mPFMs): High-dimensional (150) Probabilistic Functional Modes estimated from resting-state fMRI (1003 HCP subjects) yield a new representation of the brain’s functional organisation, which consists of an ensemble of conventional distributed-only (i.e., large-scale) resting-state networks (RSNs) and newly added modes across more fine-grained scales. We refer to this new representation as Multiscale Probabilistic Functional Modes, mPFMs. When paired with large-scale RSNs from 25-mode decomposition (yellow), 25 of the mPFMs showed a clear matching, labelled as Primary mPFMs (orange). Secondary mPFMs (blue) are the remaining 125 mPFMs, which start appearing with increased dimensionality. A force-directed layout is used to visualise the spatial similarities (i.e., correlations) between the modes. In this layout, each circle denotes a functional mode, which is a node in the connectivity matrix, and each line is the connection between a pair of modes. The size of each mode shows the average strength of its connections, line widths denote the strength of spatial correlations (i.e., overlaps) between two modes, and the distance between the modes denotes how close two modes are in the connectivity matrix, both within and between low-dimensional versus high-dimensional decompositions. All connections within-large-scale and within-multiscale, as well as paired connections between large-scale and multiscale were maintained in visualisation. Matrix-based visualisation of these results is shown in Supplementary Fig. S2.

We report that this added flexibility in modelling population variations, and in spatiotemporal connectivity between the modes, resulted in the discovery of mPFMs (Fig. 1), which preserve conventional large-scale modes, and add new modes with different scales of granularity to the ensemble. Fig. 1 shows direct comparisons between lowD versus highD Probabilistic Functional Modes (PFM) decomposition of rfMRI data in HCP, where a force-directed network layout is used for visualisation (Bastian et al., 2009). LowD decomposition (shown in yellow) yielded the conventional distributed-only resting-state networks, hereafter referred to as large-scale RSNs. The highD decomposition yielded a mixture of modes across various scales. By spatial pairing of the modes from highD and lowD decompositions, we found a one-to-one correspondence between a set of highD modes (shown in orange) and large-scale RSNs, indicating that as we increase the dimensionality from 25 to 150, conventional RSNs are preserved, and new modes are added (shown in blue). This is a novel type of decomposition, and since it includes functional modes across multiple scales, we refer to it as Multiscale Probabilistic Functional Modes, mPFMs.

mPFMs can be considered an alternative to conventional (“single scale”) resting-state networks (RSNs). We report that (a) mPFMs emerge to explain the existence of multiple distinct time courses within each large-scale mode, thus capturing voxel-to-voxel variability of temporal dynamics that cannot be captured by these distributed modes alone; (b) we validate mPFMs using several methods, including synthetic data, reproducibility between Human Connectome Project (HCP) and UK Biobank (UKB) data, and link to HCP’s Multimodal Parcellation (Glasser et al., 2016); (c) we further show the added benefit of cross-scale interactions of mPFMs, compared with within-scale interactions, for capturing cross-individual variability and predicting personalised traits; (d) finally, we show that, compared with two commonly used techniques, spatial ICA modes and parcellations from the Schaefer Atlas (Schaefer et al., 2018), mPFMs (of matched high dimensionality) yield more accurate prediction of a wide range of imaging- and non-imaging-derived phenotypes (IDPs/nIDPs) related to cognition, cardiovascular health, brain anatomy, and task fMRI in UK Biobank.

## 2 Materials and Methods

Supplementary Fig. S1 shows a flowchart of different subsections of materials and methods, and their link to the corresponding results subsections.

### 2.1 Data

#### 2.1.1 Human Connectome Project (HCP)

We used minimally pre-processed fMRI data from Human Connectome Project (HCP) from S1200 data release (https://www.humanconnectome.org/study/hcp-young-adult), with acquisition and processing details outlined in Glasser et al. (2013) and Smith et al. (2013a). Resting-state data from 1003 subjects aged 22–35 years were used here and included 4 x 15-minute runs per subject (i.e., 1 hour recording per subject). With repetition time (TR) of 0.72 seconds, this dataset includes 4800 time points per individual. Study protocol of the HCP study was approved by the WU-Minn HCP Consortium’s institutional review boards, and written informed consent was obtained from all participants prior to data collection. We used two data formats, volumetric and HCP CIFTI. CIFTI data include grey matter voxels only (i.e., greyordinates), where cortical grey matter is surface registered using MSMAll (Robinson et al., 2014) and subcortical grey matter is in volumetric space. This results in inherent data differences such as signal-to-noise ratio between cortical and subcortical regions, affecting matrix factorisation techniques in, for example, finding an unbalanced number of cortical versus subcortical modes. To prevent such unbalances and make the results more easily comparable across CIFTI and volumetric fMRI, we only used cortical greyordinates of CIFTI in this paper. ICA-FIX was used to remove artefacts from volumetric data before resampling to 32k_fs_LR space and projecting onto the surface. Standard HCP preprocessing includes spatial smoothing with a 2 mm FWHM smoothing kernel. We applied additional smoothing to obtain 5 mm FWHM (for both volumetric and CIFTI data), which improves the signal-to-noise ratio (SNR) and was found to be useful for obtaining reliable subject-specific modes (Farahibozorg et al., 2021).

HCP data were predominantly used for analyses that aimed at examining time course and functional connectivity modelling using mPFMs, their reproducibility across datasets (HCP and UK Biobank), and validation of mPFMs against existing functional parcellations. Given the longer recordings per subject (1 hour vs. 6 minutes), we deemed HCP more suitable for these analyses.

#### 2.1.2 UK Biobank (UKB)

We randomly selected 4999 subjects aged >45 years from the May 2019 release of UK Biobank data (application number 8107). The UKB has approval from the North West Multi-Centre Research Ethics Committee (MREC) to obtain and disseminate data and samples from the participants (http://www.ukbiobank.ac.uk/ethics/), and these ethical regulations cover the work in our study. Written informed consent was obtained from all participants prior to data collection. Resting-state fMRI data in UKB (at the time that this research was conducted) were in standard volumetric space, and included one recording of ~6 minutes per subject. With a TR of 0.735 seconds, data consisted of 490 (occasionally 530) time points per session. The standard UKB pipeline, which includes quality control, brain extraction, motion correction, artefact rejection using FSL-FIX, high-pass temporal filtering (sigma = 50.0 seconds, Gaussian-weighted least-squares straight line fitting), and registration to standard MNI-2 mm space, was used for preprocessing (Alfaro-Almagro et al., 2018). Standard UKB preprocessing includes spatial smoothing with a 2 mm FWHM smoothing kernel. We applied additional smoothing to obtain 5 mm FWHM, which improves SNR and is useful for obtaining reliable subject-specific high-dimensional PFMs. UKB was used for evaluating reproducibility of mPFMs (i.e., high-dimensional PFMs) across datasets, and their prediction accuracy for personalised traits. Given the wide range of imaging-derived phenotypes (IDPs) and non-imaging-derived phenotypes (nIDPs) in UK Biobank, this dataset was deemed more suitable for prediction of traits (see Section 2.6 for details).

### 2.2 Estimating resting-state networks

#### 2.2.1 Probabilistic functional modes (PFMs)

Details of PROFUMO/sPROFUMO models and inference are elaborated in our previous work (Farahibozorg et al., 2021; Harrison et al., 2020). Here we provide a brief summary of the model and its application to the data. sPROFUMO is a matrix factorisation model for big fMRI data, which uses hierarchical Bayes with two levels of modelling and inference: population and individual.

At the subject level, fMRI time series (**D**^sr^) are decomposed into a set of spatial maps (**P**^s^), time courses (**A**^sr^), and time course amplitudes (**H**^sr^), with residuals $\varepsilon^{sr}$ :

$$\begin{array}{l} D^{sr}=P^{s}H^{sr}A^{sr}+\varepsilon^{sr}D^{sr}\in {\mathbb{R}}^{N_{v}\times N_{t}},P^{s}\in {\mathbb{R}}^{N_{v}\times N_{m}},\\ \;\;\;\;\;\;\;\;\;\;\;\;\;\;\;\;\;H^{sr}\in {\mathbb{R}}^{N_{m}\times N_{m}},A^{sr}\in {\mathbb{R}}^{N_{m}\times N_{t}}, \end{array}$$ (1)

where s denotes subject, r denotes a recording session, and **H**^sr^ denotes mode amplitudes. N_v_, N_m_, and N_t_ denote the number of voxels, modes, and time points, respectively.

**P**^s^ denotes spatial mode layout across brain voxels, hereafter referred to as *spatial maps*, or just *maps*. These are modelled using a Double-Gaussian Mixture Model (DGMM), with one Gaussian component used to model signal and a second Gaussian distribution to model the background spatial noise in each voxel. To model the spatial maps hierarchically, in addition to **P**^s^ for each subject, a consensus set of group-level parameters is also estimated to capture both the group mean and variance. **A**^sr^ represents mode time courses per subject and scan. The time courses also model signal and noise elements separately, where the signal element is constrained by the Haemodynamic Response Function (HRF) and the noise element follows a Gaussian distribution. Connectivity between the time courses of functional modes can have a consensus structure across individuals. To model this simultaneously for population and individuals, a second hierarchy is defined over the precision matrix $\alpha^{sr}$ using Wishart distributions. To further allow for modelling of haemodynamic responses that govern the Blood Oxygenation Level Dependent (BOLD) signal, HRF-constrained autocorrelations are incorporated in the modelling of this functional connectivity between the modes. **H**^sr^ denotes mode amplitudes. These are modelled using a multivariate normal distribution, with a Bayesian hierarchy between the group and the subject levels. **H**^sr^ is diagonal (one value per PFM) and captures time course variance for each subject and recording.

To find a solution for the probabilistic model described above, sPROFUMO uses stochastic Variational Bayesian (VB) inference. This includes dividing subjects from large data into small batches, and optimising the parameters of an approximating distribution q, with the aim that it is as close as possible to the true posterior. The group model is maintained across batches and continuously updated over time, and within each iteration of each batch, it is used for top-down regularisation of subject-specific matrix factorisations, from which the posterior distributions for subject-specific spatial maps, time course correlations, and amplitudes are inferred. Subsequently, posterior distributions are accumulated across individuals and fed back to the group to update the group model. The model iterates between these two levels of estimation until convergence.

We used the model’s default parameters for application to both HCP and UKB datasets. The forget rate (β) was set to 0.6; this controls the degree to which global parameter updates rely on the current compared with previous batches. Delay parameter (τ) was set to 5; this controls the degree to which initial batches influence the overall inference. Batch size was set to 50 subjects, and the number of batches was tuned such that each subject will be visited 2.5 times on average (i.e., normally chosen in 2 to 3 batches). Given the different number of subjects in the two datasets, to achieve the abovementioned number of updates per subjects, the overall group model was set to be updated at least 5000 times for UKB and 4000 times for HCP. For the UK Biobank (taking 150-mode decomposition as an example), the model was run on 10 processing cores on a compute node, taking ~210 hours to complete, and the memory usage peaked at ~100 GB. For the HCP (taking 150-mode decomposition as an example), the model was run on 10 processing cores on a compute node, taking ~103 hours to complete, and the memory usage peaked at ~90 GB.

When applying to each dataset, and as a part of sPROFUMO’s internal initialisation, voxel-wise de-meaning and variance normalisation were applied to rfMRI recordings. Additionally, to initialise the model using a realistic set of initial group maps that can place the parameters within a realistic ballpark, sPROFUMO internally applies an online PCA algorithm called MELODIC’s Incremental Group-PCA (MIGP) (Smith et al., 2014) followed by variational ICA. These initial maps, together with hyperpriors, are used to initialise the group-level parameters before conducting the full Bayesian inference at subject level and group level. It is worth noting that, to make the results fully comparable across HCP and UKB, the same set of initial maps were used to initialise both datasets. Two features in sPROFUMO help the model escape local minima: firstly, we use 10 random re-initialisations to generate the initial group-level maps; secondly, the mini-batching technique in stochastic variational Bayesian inference reduces the risk of getting stuck in local minima.

We estimated 25, 50, 100, and 150 PFMs, each used in a subset of the analyses, as elaborated in the Results section. Initial maps were estimated for each dimensionality independently, since the number of the initialised modes will have to match the final number of the estimated modes. However, it is worth noting that the probabilistic framework has the capacity to switch off some of these modes in the final decomposition, if there is not enough posterior evidence in the data to support specific modes in higher dimensionalities (e.g., in noisier datasets or noisier modes for specific subjects). This feature, which reduces the risk of false-positive modes, has been investigated in detail in our previous work (Farahibozorg et al., 2021).

After estimating PFMs with various dimensionalities, we validated whether each mode existed at both group level and subject level. A few artefactual modes (typically ~2–3 due to, e.g., white matter artefacts) were manually removed from subsequent analyses. At subject level, spatial and temporal aspects of the modes were used for evaluation. In particular, subject-to-group similarity of spatial maps is a useful metric. Very low similarities (~<0.1) would indicate highly noisy estimation of a mode at subject level. Whereas, artefactually high similarities (>0.99) would indicate that posterior parameters were not estimable at subject level, and thus, the spatial map for that specific subject-PFM was reverted back to group priors. An example of subject–group spatial map similarity for d = 150 in UK Biobank is shown in Supplementary Fig. S3. In addition to this, we used mode amplitudes (H^sr^) and standard deviation of spatial maps across voxels to identify PFMs with weak signal at subject level. PFMs included in the subsequent analyses were reliably estimated at both subject level and group level.

##### 2.2.1.1 Volumetric versus CIFTI PFMs

sPROFUMO was applied to volumetric and CIFTI data from HCP separately, and group-level spatial maps were used as the basis for computing the reproducibility scores reported in the Results section 3.2.2. In order to compare volumetric and CIFTI results, volumetric PFMs were mapped onto CIFTI using linear regression at the subject level. More specifically, for each subject and run, we used volumetric PFM time series (${\text{A}}_{VOL}^{sr}$ ) and amplitudes (${\text{H}}_{VOL}^{sr}$ ), as well as raw CIFTI fMRI time series ${\text{D}}_{CIFTI}^{sr}$ . We computed spatial maps in CIFTI space (${\text{P}}_{CIFTI}^{s}$) through back projection of ${\text{A}}_{VOL}^{sr}{\text{H}}_{VOL}^{sr}$ onto ${\text{D}}_{CIFTI}^{sr}$ using linear regression with L2 regularisation. Regression betas were then converted to t-stat values. We next averaged ${\text{P}}_{CIFTI}^{s}$ across subjects and obtained volume-to-surface mapped group spatial maps to compare against surface-based spatial maps.

#### 2.2.2 Spatial ICA and dual regression

We applied the widely used group-level spatial ICA followed by dual regression (DR) to characterise subject-specific RSNs and compare against sPROFUMO results. In standard pipelines of large datasets such as HCP and UKB, ICA/ICA-DR have been used for RSN characterisation, thus providing a suitable baseline to compare sPROFUMO results against. We applied ICA to UKB data and identified 100 group-level spatial components using FSL *MELODIC*, and subsequently mapped these group-level results onto single subject data using FSL *dual_regression*. Subject-specific time courses for each mode and subject-specific spatial maps were obtained from stages 1 and 2 of Dual Regression, respectively. Mode amplitudes were computed as standard deviation of the time courses and functional connectivity was computed using Tikhonov/L2-regularised partial correlations. In order to make the final sPROFUMO and ICA/ICA-DR results fully comparable, the same PCA initialisation (using the MIGP approach) was used for both models. ICA-DR results were used to predict non-imaging phenotypes and compare PFM’s performance against.

### 2.3 Identifying temporally distinct subcomponents within large-scale RSNs

Low-dimensional representation of functional modes yields large-scale RSNs that are distributed across multiple brain areas. Matrix factorisation techniques (e.g., sPROFUMO or ICA) describe each of these modes with a single time course. We tested whether there are distinct temporal subcomponents within each of these modes, and whether or not these subcomponents will be better represented by lowD large-scale RSNs versus highD mPFMs. For this purpose, we focused on 25-mode decomposition from HCP CIFTI rfMRI data of 1003 subjects, and conducted the following tests:

#### 2.3.1 Characterising distinct subcomponents within each conventional RSN

This step aimed to test whether a single prominent subcomponent can explain a majority of *group-level* temporal variance in the voxel-wise activity within each large-scale mode. For this purpose, we used PCA as follows:

- N_voxel_ × N_time_ subject rfMRI time series were weighted by each subject PFM (d = 25) spatial map, obtaining N_voxel_ × N_time_ weighted rfMRI time series (the data dimensions are unchanged, but voxels within a given map are up-weighted);
- PCA was applied to each of these weighted time series, and 10 components were estimated per subject per mode, reducing the weighted subject fMRI data of each mode to N_voxel_ × 10. The resulting PCA maps (for each mode separately) were concatenated across subjects, resulting in a feature space of size N_voxel_ × (10 × N_subject_). A second group-level PCA was next applied, and 10 group-level components were extracted per mode. The explained variance per PC was estimated (by considering the 10 singular values); the results of this analysis are reported in 3.1.1.

#### 2.3.2 Are temporal subcomponents best represented by mPFMs?

This step aimed to test whether multiple temporally distinct subcomponents exist within each large-scale RSN (i.e., distributed only), and whether subcomponents originating from large-scale RSNs can be better represented by large-scale (lowD) versus multiscale (highD) PFMs. This was done using temporal ICA, and further replicated using spatial ICA and PCA. This step, as well as the subsequent steps in 2.3.3 focused on *single-subject* modes.

- Similar to step 2.3.1, N_voxel_ × N_time_ subject rfMRI time series were weighted by each subject PFM (d = 25) spatial map, and 5 spatial PCs were extracted per mode per subject. The choice of 5 PCs was determined based on results of step 2.3.1, where PCs > 6 were found to explain less than 3% of variance for all the PFMs (Supplementary Fig. S4).
- For each PFM, the five PC maps were concatenated across subjects and group-level PCA was used to extract five spatial subcomponents per PFM.
- These group-level PCs were then linearly regressed onto each subject’s PFM-weighted rfMRI time series (as described in step 2.3.1a), giving five temporal components per subject per mode, which were then concatenated across subjects to obtain five long time series per PFM.
- Temporal ICA was applied to the resulting long time series to obtain time series for five temporally independent subcomponents. These time series were split up into temporal chunks to obtain subject-specific tICA subcomponent time series.
- Subject-specific tICA subcomponent time series were linearly regressed onto each subject’s PFM-weighted rfMRI time series (as described in step 2.3.2a) to obtain subject-specific tICA subcomponent spatial maps.
- By correlating the subject-specific time courses of each subcomponent with all the original lowD and highD mode time series, we applied a winner-takes-all approach to determine how many of the subcomponents originating from large-scale lowD modes were best represented by lowD versus primary versus secondary mPFMs. An unpaired t-test was used to conduct statistical comparisons, and results were Bonferroni corrected for multiple comparisons.

#### 2.3.3 Do secondary mPFMs emerge out of areas of spatial overlap and/or temporal co-activation?

1. This step aimed to test whether subcomponents identified in step 2.3.2 were exclusive to one lowD mode, or whether they resided in the *temporal co-activation periods* shared by multiple lowD modes:
  - Similar to step 2.3.2a, N_voxel_ × N_time_ subject fMRI time series were weighted by each subject PFM (d = 25) spatial map, obtaining 25 weighted fMRI datasets per subject.
  - Linear regression was used to regress out the time courses of every other (24) PFM from each PFM’s weighted fMRI. Then five PCs were extracted per mode per subject.
  - Steps 2.3.2b-f were repeated to test whether subcomponents originating from the temporally exclusive space of lowD modes were best represented by lowD versus primary versus secondary mPFMs.
2. This step aimed to test whether subcomponents identified in step 2.3.2 were exclusive to a single lowD mode, or whether they resided in the *spatial* space shared by multiple lowD PFMs:
  - Linear regression was used to regress out the spatial maps of every other (24) PFM from each PFM’s spatial map.
  - N_voxel_ × N_time_ subject fMRI time series were weighted by the resulting subject PFM (d = 25) spatial map after regression, obtaining 25 weighted fMRI datasets per subject.
  - Linear regression was used to regress the spatial maps of every other (24) PFM from each PFM’s weighted fMRI. Then five PCs were extracted per mode per subject.
  - Steps 2.3.2b-f were repeated to test whether subcomponents originating from the spatially exclusive (non-overlapping) space occupied by lowD modes were best represented by lowD versus primary versus secondary mPFMs.

### 2.4 Simulations

Based on real data (Methods section 2.3 and Results section 3.1), we found that Secondary mPFMs were likely due to the existence of temporally distinct subcomponents within the Primary mPFMs, that is, the ensemble would capture integrated-segregated modes of brain connectivity. Here we simulated datasets where overlapping distributed and localised RSNs exist in the brain, such that localised RSNs are spatial sub-nodes of distributed RSNs but have distinct time courses. Using these simulations, we tested how well PFM decomposition can recover the co-existence of global–local RSNs in the brain, and compared its performance with spatial ICA as the standard technique. Details of simulations were as follows:

- Data: 10 resting-state fMRI datasets were simulated, each consisting of 50 subjects and 2 runs per subject.
- Twelve resting-state modes were simulated in each dataset, and they were created from scratch in each dataset.
- Six of these modes were distributed, covering ≥  2 brain areas, 6 of these modes were localised, covering 1 brain area. Distributed modes were allowed to spatially overlap with each other, such that on average, 1.3 modes were included in a given voxel.
- Localised modes were simulated such that they were primarily a spatial sub-node of one distributed mode, while spatially overlapping with two or multiple distributed modes.
- Data properties per subject and run: 10,000 voxels, 300 time points at a TR of 0.72 seconds. Data were created as detailed below:

We defined a set of spatial maps **P_g_** at the group level. Each mode consisted of one or more randomly selected contiguous blocks of voxels (i.e., parcels). Signal weights (per voxel per mode) were drawn from a Gamma distribution. Subject-specific spatial maps, **P_s_**, were defined based on the group maps by adding background Gaussian noise and applying spatial warps. Subject maps were generated to be spatially misaligned in reference to the group, with any given subject mode having on average 83% overlap with the group average (i.e., 17% spatial misalignment). Time courses were generated independently for each session, subject, and mode to mimic the unconstrained resting-state data (but following the between-mode temporal correlation structure described next). We assigned a hierarchical link between the group and subjects’ temporal correlation matrices, following a Wishart distribution. This was done to obtain a consistent functional connectivity pattern between subjects and the group. Time courses were initially simulated as semi-Gaussian neural time courses with amplified frequencies <0.1 Hz, and subsequently convolved with a random draw from the FLOBS haemodynamic basis functions (Woolrich et al., 2004) to mimic realistic BOLD signal. Finally, data were created using outer product of subject mode spatial maps and time courses, and random noise was added to the outer product to create space-time data matrices. More details of simulation parameters are available in Harrison et al. (2020).

These simulated modes were then estimated using PFM decomposition and ICA-Dual Regression, and results were compared with the ground truth. We initialised both models based on the same set of spatial bases from MIGP, to ensure that the observed differences were not due to initialisation of the probabilistic models.

### 2.5 mPFMs versus HCP-MMP

We applied sPROFUMO to cortical rfMRI data of 446 HCP subjects (matching the number of subjects for which HCP-MMP was available) and estimated 360 PFMs. The two decompositions were compared at two levels: First, we computed group correspondence between mPFMs and HCP-MMP. We binarised group-level mPFMs’ spatial maps by hard thresholding at 0.3 and computed their dice similarity to group-level parcels in HCP-MMP. Second, we examined subject-level correspondence by binarising subject-level mPFMs’ spatial maps by hard thresholding, and finding dice similarity between binarised mPFMs and subject-level parcels in HCP-MMP. The modes were paired based on group-level matching, and this ordering was used in both group-level and subject-level visualisations.

### 2.6 Out-of-sample prediction pipeline

We investigated the link between the spatial/temporal properties of mPFMs and a range of phenotypes in UKB and HCP. This was done using out-of-sample predictions in UKB and Canonical Correlation Analysis (CCA) in HCP. These accuracies were used to compare mPFMs versus conventional large-scale RSNs, primary versus secondary mPFMs, and mPFMs versus spatial ICA and Schaefer parcellation of same dimensionality. Depending on the question at hand in each subsection, we conducted predictions based on all or some of the following features: (a) Mode spatial maps, (b) Mode temporal network matrices (TNETs or temporal NetMats; partial correlation matrix between mode time courses), (c) Mode spatial NetMats (SNETS, the full spatial correlation matrix between mode maps).

#### 2.6.1 Imaging and non-imaging derived phenotypes (IDPs and nIDPs)

We used 893 imaging-derived and non-imaging-derived phenotypes (IDPs and nIDPs) from UK Biobank as targets in the prediction pipeline. These included IDPs related to grey matter, white matter and task fMRI, as well as nIDPs related to cognitive scores and cardiovascular health metrics. A brief summary of these phenotypes is included in this subsection, and a full list is included in Supplementary Tables S7–S13.

##### 2.6.1.1 Grey matter

148 IDPs related to GM area and 148 IDPs related to GM thickness, generated with Freesurfer by parcellation of the white surface using Destrieux (a2009s) parcellation. More details about these IDPs (e.g., histograms) can be found on the UKB website: https://biobank.ctsu.ox.ac.uk/crystal/label.cgi?id=197.

##### 2.6.1.2 White matter

Three IDPs extracted based on the volume of white matter hyperintensities using BIANCA. In total, 162 IDPs extracted based on mean intensity for dtifit outputs (FA, MD, MO3) and NODDI outputs (ICVF, OD, ISOVF) for the 27 tracts segmented with the probabilistic tractography analysis. In total, 288 IDPs extracted based on mean intensity for dtifit outputs (FA, MD, MO) and NODDI outputs (ICVF, OD, ISOVF) for the 48 tracts segmented with the TBSS-like analysis.

##### 2.6.1.3 Task fMRI

UKB includes data for one task, which involves brain responses to images of emotionally valenced faces and shapes. One IDP, called “contrast” in the results section, was estimated as 90th percentile (z-statistic) of activity for response to faces versus shapes (https://biobank.ctsu.ox.ac.uk/crystal/field.cgi?id=25766).

##### 2.6.1.4 Cognitive

Starting from 1172 nIDPs related to cognitive tests, we narrowed these down to 68 using 2 criteria. Firstly, one of the authors prefiltered the outcome measures manually, by only including active measures that were indicative of a subject’s performance. For example, among outputs directly related to “Reaction Time”, which entails viewing two cards (A and B) and pressing buttons when identical, “Mean time to correctly identify matches” was included whereas “Index for card A in round” was excluded. Secondly, we only selected tests that had a non-NaN value in at least 25% of the subjects. The final list of 68 tests belonged to the following categories: *Reaction time, Trail making, Matrix pattern completion, Numeric memory, Prospective memory, Pairs matching, Symbol digit substitution*, and *Fluid intelligence*. More details about these nIDPs are available here: https://biobank.ctsu.ox.ac.uk/crystal/label.cgi?id=100026.

##### 2.6.1.5 Cardiovascular health metrics

Starting from 992 nIDPs related to cardiovascular health, we narrowed these down to 77 using 2 criteria. Firstly, one of the authors prefiltered the outcome measures manually, by excluding metrics that were not directly related to health. For example, “LV stroke volume (2.0)” and “Systolic blood pressure, manual reading (0.0)” were included, whereas “Completion status of test (0.0)” and “Program category (0.0)” were excluded. Secondly, we only selected tests that had a non-NaN value in at least 25% of the subjects. More details about these nIDPs are available in the following links: https://biobank.ctsu.ox.ac.uk/crystal/label.cgi?id=100011, https://biobank.ctsu.ox.ac.uk/crystal/label.cgi?id=104 and https://biobank.ctsu.ox.ac.uk/crystal/label.cgi?id=100012.

#### 2.6.2 Confound removal in UK Biobank

When conducting brain–behaviour analyses in big data, for example, association or out-of-sample predictions, imaging confounds can significantly distort the interpretability of the results (Alfaro-Almagro et al., 2020; Snoek et al., 2019). For example, if a common confounding factor such as head size or head motion is correlated with both brain features and behavioural targets, this is likely to inflate our prediction accuracies (Snoek et al., 2019). Therefore, we opted to de-confound the data before conducting any brain–behaviour analyses. This was done by linearly regressing out the confounds from both imaging and non-imaging variables.

For UK Biobank, we started with a comprehensive set of 602 confounds proposed by Alfaro-Almagro et al. (2020). We reduced this set by (a) selecting conventional confounds including age, age squared, sex, age × sex, site, head size, and head motion; (b) reducing the remaining confounds using Principal Component Analysis. The top PCs that explained >85% of the variance were kept. These two steps yielded 82 variables. Importantly, we applied deconfounding taking into account cross-validation folds in order to avoid leakage of information from test to train data, as proposed by Snoek et al. (2019).

#### 2.6.3 Elastic-Net prediction and cross-validation

We used Python 3.6.5 and scikit-learn 0.19.1 (Pedregosa et al., 2011) to set up the prediction pipeline. We used ElasticNet regression and nested 5-fold cross-validations to conduct out-of-sample predictions, with 20/80 test/train ratio. Next, we Gaussianised each predictor and target variable across subjects using quantile transformation (*QuantileTransformer*). In each cross-validation loop, the training set was used to compute the quantile transforms which were then applied to both train and test data. Similarly, deconfounding was also conducted within each cross-validation loop, where training set was used to estimate the regression parameters (or “betas”), which were then applied to de-confound both train and test data. Finally, we used *ElasticNetCV* to predict target variables in the test set. Hyperparameters related to ElasticNet; that is, ratio of Lasso to Lasso+Ridge regularisation (*L1 ratio* varied between [0.1, 0.5, 0.7, 0.9, 0.95, 0.99, 1.0] with 10 alphas per *L1 ratio*), were optimised using nested cross-validations within the training set. Finally, we computed correlations between estimated and actual values of the target variables across subjects in the test set, which will be reported as prediction accuracies.

#### 2.6.4 Feature spaces: Large-scale versus multiscale PFMs

The comparison between conventional large-scale RSNs and mPFMs aimed to determine any added benefit of the latter, and additionally to determine the added benefit of having both types of primary and secondary modes in a decomposition, as opposed to one type only. For this purpose, we focused on interactions between the modes, that is, spatial/temporal correlations, and their prediction power for phenotypes in UKB. Specifically, the comparison was made between prediction power of correlations in three scenarios: large-scale RSNs versus mPFMs; between primary and secondary mPFMs versus within primary mPFMs; and between primary and secondary mPFMs versus within secondary mPFMs.

To summarise mode interactions, two types of feature matrices were used, Spatial NetMats (SNETs) and partial temporal NetMats (TNETs). TNETs were computed based on precision matrices and with a Tikhonov regularisation parameter 0.01. SNETs and TNETs are of size N_mode_ × N_mode_ for each subject in each PFM decomposition.

Based on highD decomposition (d = 100), we divided each of the NetMats into three sub-matrices

1. SNET_pri2pri_: N_pri_ × N_pri_ and TNET_pri2pri_: N_pri_ × N_pri_, with N_pri_ = 25
2. SNET_sec2sec_: N_sec_ × N_sec_ and TNET_sec2sec_: N_sec_ × N_sec_, with N_sec_ = 75
3. SNET_pri2sec_: N_pri_ × N_sec_ and TNET_pri2sec_: N_pri_ × N_sec_
4. Based on large-scale lowD decomposition (d = 25), we obtained SNET_low_dim_: N_lowD_ × N_lowD_ and TNET_low_dim_: N_lowD_ × N_lowD_. Similarly, based on multiscale highD decomposition (d = 100), we obtained SNET_high_dim_ : N_highD_ × N_highD_ and TNET_high_dim_ : N_highD_ × N_highD_.

We applied the Fisher r-to-Z transformation, and unwrapped these matrices into 1D vectors. Since Pri2Pri, Sec2Sec, highD2highD and LowD2LowD matrices (but not Pri2Sec) are symmetric, only the upper diagonal elements were kept before unwrapping. Next, we used SVD to dimension reduce each of these ten N_subject_ × N_edges_ matrices to feature matrices of size N_subject_ × 200, which were fed into ElasticNet. The dimensionality reduction was done to obtain the same number of features to use in predictions, reducing the possibility of one mode type outperforming due to the number of features. Five-fold cross-validations were performed and accuracies computed, as detailed in Section 2.6.3. Cross-validations were repeated k times for each phenotype category, k being: GM: 20, WM: 20, task fMRI-contrast (since there is only 1 phenotype in this category): 200, cognitive: 20, cardiovascular metrics: 20.

For each IDP/nIDP category, accuracies were computed for each phenotype, and pooled across the number of phenotypes in that category (N_phenotype_) and number of repeats (k), yielding two vectors of size k* N_phenotype_ to be compared between different mode types.

#### 2.6.5 Feature spaces: mPFMs versus standard techniques

##### 2.6.5.1 mPFMs and spatial ICA features

The comparison of mPFMs with highD ICA aimed to examine any added benefit of mPFMs over state-of-the-art RSN decompositions for capturing individualistic traits and phenotype predictions. For this purpose, three types of feature matrices were used in predictions, spatial maps (SMAPs), SNETs, and TNETs. HighD decompositions with 100 RSNs from UKB data were used in these analyses. This dimensionality was optimised based on a left-out phenotype (age): we compared age prediction using 50, 100, 150, and 200 dimensional decompositions and found 100 to be optimal for both mPFMs and highD ICA. It is worth noting that d = 100 was found to be optimal here for UK Biobank data. The optimal choice may vary for datasets with a smaller number of subjects, or datasets with longer recording sessions per subject. Feature spaces were estimated using a pipeline similar to the Uni-mode predictions described in our previous study (Farahibozorg et al., 2021):

- For SMAPs, estimating 100 modes resulted in a matrix of N_subject_ × N_voxel_ × 100. We performed dimensionality reduction across the second dimension (per mode) using sparse dictionary learning (Mairal et al., 2010) and obtained feature matrix of size (N_subject_ × 500) × 100. Next, we used the feature space of each PFM separately in phenotype predictions.
- For SNETs and TNETs, we started with matrices of size N_subject_ × N_mode_ × N_mode_. After applying the Fisher r-to-Z transformation per subject, we used rows of correlation matrices (i.e., one per mode) for each subject without additional dimensionality reduction, thus obtaining 100 feature matrices of size N_subject_ × 99 from SNETs and 100 feature matrices of size N_subject_ × 99 from TNETs that were used separately for predictions. TNETs were computed using partial correlations with Tikhonov regularisation.

##### 2.6.5.2 Schaefer parcellation features

The standard Schaefer parcellation in MNI space, Schaefer2018_100Parcels_17Networks_order_FSLMNI152_2mm.nii.gz, from https://github.com/ThomasYeoLab/CBIG/tree/master/stable_projects/brain_parcellation/Schaefer2018_LocalGlobal/Parcellations/MNI was applied to pre-processed fMRI time series of UK Biobank subjects; time courses of voxels within each parcel were averaged to obtain one time course per parcel/mode. Subsequently, similar to the PFM/sICA procedure explained above, TNET features were computed from these time courses using Fisher r-to-Z transformed partial correlations with Tikhonov regularisation. These resulted in 100 feature matrices of size N_subject_ × 99 which were used in Uni-mode predictions.

##### 2.6.5.3 Summary of features from mPFM/sICA/Schaefer

We used features of each mode separately in predictions.

On the one hand, for PFM and sICA, SMAP, SNET, and TNET feature matrices were concatenated horizontally, yielding 100 feature matrices of size N_subject_ × 799 to use in cross-validated ElasticNet predictions, as elaborated in Section 2.6. Accuracies were computed for each mode and pooled across modes to compare PFMs and ICA-DR.

On the other hand, mPFMs and spatial ICA both yield spatial and temporal features, whereas hard parcellations such as Schaefer generate TNET features only. This is due to the fact that hard parcellations yield binarised spatial topographies for the modes/parcels, with fixed hard boundaries between the parcels. Therefore, the number of features derived from the Schaefer parcellation will be lower. To balance this number across the three methods, we used cross-validated feature selection based on correlation to the target in the training set to select a subset of features from mPFM/sICA, matching the number of features (99 per mode) from the Schaefer parcellation.

For each phenotype category (cardiovascular, cognitive, etc.), accuracies were computed for each mode and each phenotype, and pooled across the number of phenotypes in that category (N_phenotype_) and number of modes (100), yielding three vectors of length 100* N_phenotype_ to compare mPFMs with ICA-DR and the Schaefer Parcellation.

### 2.7 Canonical correlation analysis

Canonical Correlation Analysis (CCA) was used to find a single multivariate mapping between a set of PFM features and a set of non-imaging variables. Each CCA component estimates a linear combination of PFM features and a linear combination of phenotypes, such that the transformed outputs are maximally correlated. In other words, CCA components project data onto common axes of subject variability that co-vary between brain and behaviour. In previous studies of rfMRI functional connectivity using spatial ICA, CCA has identified a positive–negative axis of brain–behaviour associations in HCP data (Smith et al., 2015). Here, using HCP and focusing on the same set of phenotypes, we conduced CCA aiming to examine the behavioural relevance of mPFMs in the context of existing literature. In total, 1001 out of 1003 subjects who were included in both fMRI and phenotype recordings were included in this analysis.

#### 2.7.1 Non-imaging phenotypes

We used 158 phenotypes for the CCA. These were sub-selected from a wider range of phenotypes using a set of criteria described in Smith et al. (2015), and included metrics related to cognition such as various measures of fluid intelligence, executive function, language, episodic memory, working memory, attention; metrics of emotion such as life satisfaction, friendship, loneliness; metrics of psychiatric and life function such as depression, anxiety, aggression; metrics of alertness related to sleep, five metrics of personality related to agreeableness, extraversion, neuroticism, conscientiousness, and openness, metrics of physical health related to sensory–motor function; and lifestyle metrics related to substance use such as alcohol, tobacco, and drug. A full list of these phenotypes is presented in Supplementary Table S14.

#### 2.7.2 Confound removal in HCP

Similar to Section 2.6.2, we de-confounded both feature and phenotype matrices before conducting CCA. For this purpose, we used 13 imaging confounds that are included regularly in HCP studies: acquisition reconstruction software version; age; age squared; sex; age × sex; sex × age squared; race, ethnicity; height; weight; a summary statistic quantifying average subject head motion during the resting-state fMRI acquisitions; the cube-root of total brain volume (including ventricles), as estimated by FreeSurfer; and the cube-root of total intracranial volume, as estimated by FreeSurfer.

#### 2.7.3 mPFM feature spaces

For CCA, we need a single feature matrix of size N_subject_ × N_feature_ estimated from brain imaging to be paired with a single phenotype matrix of size N_subject_ × N_phenotype_ from behavioural/non-imaging traits. Therefore, we required a further condensed feature space compared with what described earlier in trait prediction. We extracted feature summaries separately from SMAPs, SNETs, and TNETs, and ran CCA using each feature type independently. Feature summaries were created as follows:

- For the spatial maps (SMAPs), estimating 150 modes resulted in a **P**_grand_ matrix of (N_subject_ × N_voxel_) x 150, yielding ~35 million features per subject. To extract a few hundred features that can meaningfully capture the essence of subject SMAPs, we used unsupervised learning by applying FMRIB’s Linked ICA for big data (BigFLICA) (Gong et al., 2021; Groves et al., 2011). This is an ICA framework originally proposed for multimodal data fusion, and was applied here in two steps. First, subject SMAPs were dimension reduced across voxels using sparse dictionary learning, yielding a matrix of size (N_subject_ × 1000) × 150. Next, each PFM was used as a separate “modality” within bigFLICA to obtain a feature matrix of size N_subject_ × 500. Using FLICA here allows us to preserve subject-specific variations in each mPFM and efficiently summarise them across numerous modes to obtain a set of independent features to characterise the sources of population variations in mPFMs.
- For the spatial and partial temporal correlation matrices (SNET, TNET), we started with matrices of size N_mode_ × N_mode_ for each subject. After applying the Fisher r-to-Z transformation, we flattened these matrices by taking the above-diagonal elements. With 150 PFMs, this resulted in two feature matrices, one for SNET another for TNET, each of size N_subject_ × (150 × 149 / 2) = N_subject_ × 11175.

Each of these three matrices was used separately in CCA estimation, and results were combined post hoc, as elaborated in the next subsection.

#### 2.7.4 Conducting CCA

CCA consisted of the following steps:

1. One side of CCA received mPFM feature matrices as input: SMAPs: 1001 × 500 (N_subject_ × N_FLICA_); SNETs: 1001 × 11175 (N_subject_ × N_NET_ELEMENT_); TNETs: 1001 × 11175 (N_subject_ × N_NET_ELEMENT_). Preprocessing steps similar to Smith et al. (2015) were applied to these matrices, which included normalisation and dimensionality reduction using SVD. Each feature matrix was reduced to a matrix of size 1001 × 50. The dimension reduction helps to avoid an over-determined (rank deficient) CCA solution.
2. The other side of CCA received phenotype matrices as input: 1001 × 158. Preprocessing steps similar to Smith et al. (2015) were applied to these matrices, which included inverse Gaussian transformation and dimensionality reduction using SVD. The phenotype matrix was reduced to a matrix of size 1001 × 50.
3. Imaging confounds were regressed out of both phenotype and PFM feature matrices, as elaborated in Section 2.6.2.
4. CCA was conducted for three pair-wise comparisons: (1) PFM SMAP versus phenotypes; (2) PFM SNET versus phenotypes; (3) PFM TNET versus phenotypes. This was done using the “canoncorr” function in Matlab.
5. CCA yields a linear transformation of PFM feature matrix (X) and phenotype feature matrix (Y) so as to maximise their correlation, that is, Y × A = U ∼ X × B = V, where U and V are the linearly transformed versions of phenotype and PFM feature matrices, respectively.
6. By finding correlations between columns of U and V for the top 50 CCA components, we estimated shared variances for each pairwise comparison.
7. We finally tested how many of the CCA components were significantly correlated. For this purpose, we conducted multi-level block permutations (Winkler et al., 2015), which takes family structure of HCP data into account. In each iteration, X in step 5 was kept fixed, and rows of Y were randomly permuted (i.e., permuting subjects, while keeping family members together), and step 6 was repeated. Across 10,000 permutations, we constructed a null distribution for the correlations of the top 50 CCA components. The correlation value corresponding to the top 5% of the null distribution for the *first* CCA component was used as threshold for p-value <0.05 significance level for all the CCA components. This yields a significance threshold that is Family-Wise Error-rate (FWE) corrected for multiple comparisons (across CCA components).

## 3 Results

**Fig. 2.**
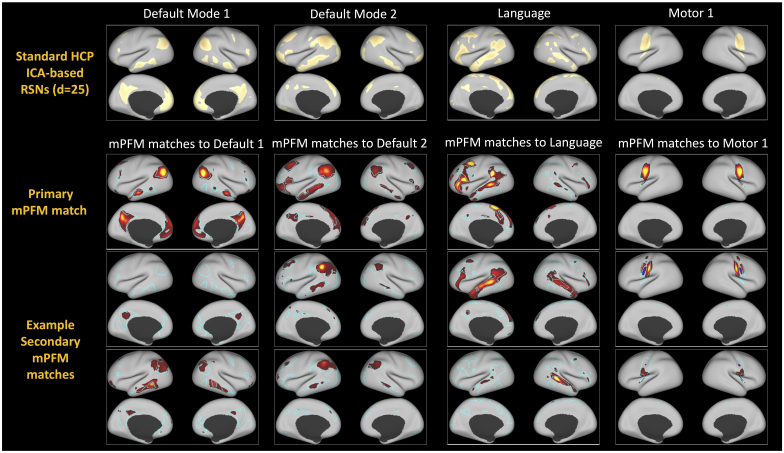
Four well-known RSNs and example primary and secondary mPFM matches to each. Top panel shows example well-known RSNs, included in HCP’s standard resting-state networks (RSNs) releases, and estimated using ICA (d = 25). mPFMs that are shown in the middle and bottom panels are a subset of the RSNs that are obtained from a high-dimensional (d = 150) decomposition of cortical rfMRI data from 1003 HCP subjects. mPFMs are overlayed on cyan borders of the ICA-based RSNs for easier visual comparison. As can be noted, primary mPFMs are, in principle, the well-known RSNs: Default Mode 1 (DMN1), Default Mode 2, Language and Motor 1 networks. Secondary mPFMs depicted below show subsystems of these networks.

Fig. 2 illustrates four examples of the well-known large-scale RSNs, estimated using ICA in HCP’s standard pipelines. Below, we have shown one primary mPFM (the closest spatial match) and two example secondary mPFMs, matching subsystems of these RSNs. Default Modes (DMN1 and 2) closely resemble the two DMNs that have been reported in the literature (Andrews-Hanna et al., 2010), and distinct from each other in various ways, for example, DMN1 features a para-hippocampal node that is missing in DMN2; DMN1 encompasses posterior IPL and Angular Gyrus, whereas DMN2 encompasses anterior IPL; lateral temporal areas such as the anterior temporal lobes are more pronounced in DMN2; DMN1 encompasses ventromedial PFC, whereas DMN2 encompasses dorsomedial PFC. The secondary mPFMs under each DMN highlight a few of their subnodes that contribute to distinct functionalities. Similarly, the illustrated language network has been reported in previous meta-analytic analyses of language processing in the brain (Lipkin et al., 2022), featuring a predominantly left-lateralised network that covers temporal, inferior parietal, inferior frontal, and pre-motor regions. Each of the secondary mPFMs then highlight subparts of this network. Finally, one of the brain’s motor networks located at the central sulcus and two of its subsystems are shown on the right-hand side of the figure. In the next section, we dive into understanding what these mPFMs are, and why they appear as separate, yet overlapping, entities in high-dimensional decomposition of resting-state fMRI (rfMRI) data using sPROFUMO.

We applied sPROFUMO to rfMRI data from 1003 HCP subjects and separately to 4999 UK Biobank subjects. Results predominantly focus on a 25-mode low-dimensional (lowD) PFM decomposition and its comparison with higher-dimensional (highD, >100) PFM decompositions, to investigate the functional relevance and the added benefit of mPFMs. Supplementary Table S1 includes a summary of the terminology used in the paper.

The following subsections aim to unravel the properties of the brain function that give rise to mPFMs, validating them based on simulations and real data, as well as demonstrating the utility of mPFMs for capturing individualistic traits and prediction of cognition and health. HCP data, compared with UK Biobank, provide higher data quality and longer fMRI recordings (1 hour vs. ~6 minutes) per subject. Thus, HCP data were primarily used for untangling the functional relevance of mPFMs, and how they yield a more comprehensive summary of brain activity and connectivity. UK Biobank, given its larger sample size and more extensive phenotyping, was primarily used for evaluating mPFMs’ performance in out-of-sample prediction of phenotypes. Both datasets were used for reproducibility analyses.

### 3.1 The functional relevance of mPFMs

Focussing on 25 and 150 sPROFUMO RSNs from rfMRI data of 1003 HCP subjects, we investigated the properties of the brain function that underlie mPFMs, and their added benefit over large-scale (distributed-only) decompositions for capturing multiscale information processing in the brain. We use the following terminology in the rest of the paper (see Fig. 1): (a) large-scale RSNs (yellow): 25 modes from low-dimensional decomposition; (b) “primary” mPFMs (orange): 25 distributed modes from high-dimensional PFM decomposition that best-match the low-dimensional RSNs; (c) “secondary” mPFMs (blue): remaining 125 modes from high-dimensional PFM decomposition.

#### 3.1.1 Multiple distinct subcomponents within each conventional RSN

Each functional mode is typically characterised by two key characteristics: (a) the spatial topography across brain voxels (i.e., a spatial map), from which one can derive spatial correlations or overlaps with other modes; (b) a time course, and the resulting temporal correlations with other modes (i.e., functional connectivity).

This representation assumes that voxels within a mode are highly correlated with each other, such that they can be summarised with one consensus time course. In other words, it assumes that temporal variability across voxels within each RSN’s spatial map is negligible. To determine whether lowD decomposition complies with this assumption, we asked: how many components are needed to explain a majority of variance in the voxel-wise activity within each large-scale RSN? We used principal component analysis (PCA), which projects data onto new orthogonal axes of variation, and allows to identify distinct subcomponents based on the proportion of variance explained (details in Section 2.3.1). We found that (Supplementary Fig. S4) the first principal component could only explain up to 53.2% of variance of temporal variability, with the second to fifth principal components each explaining ~5–10% of variance.

**Fig. 3.**
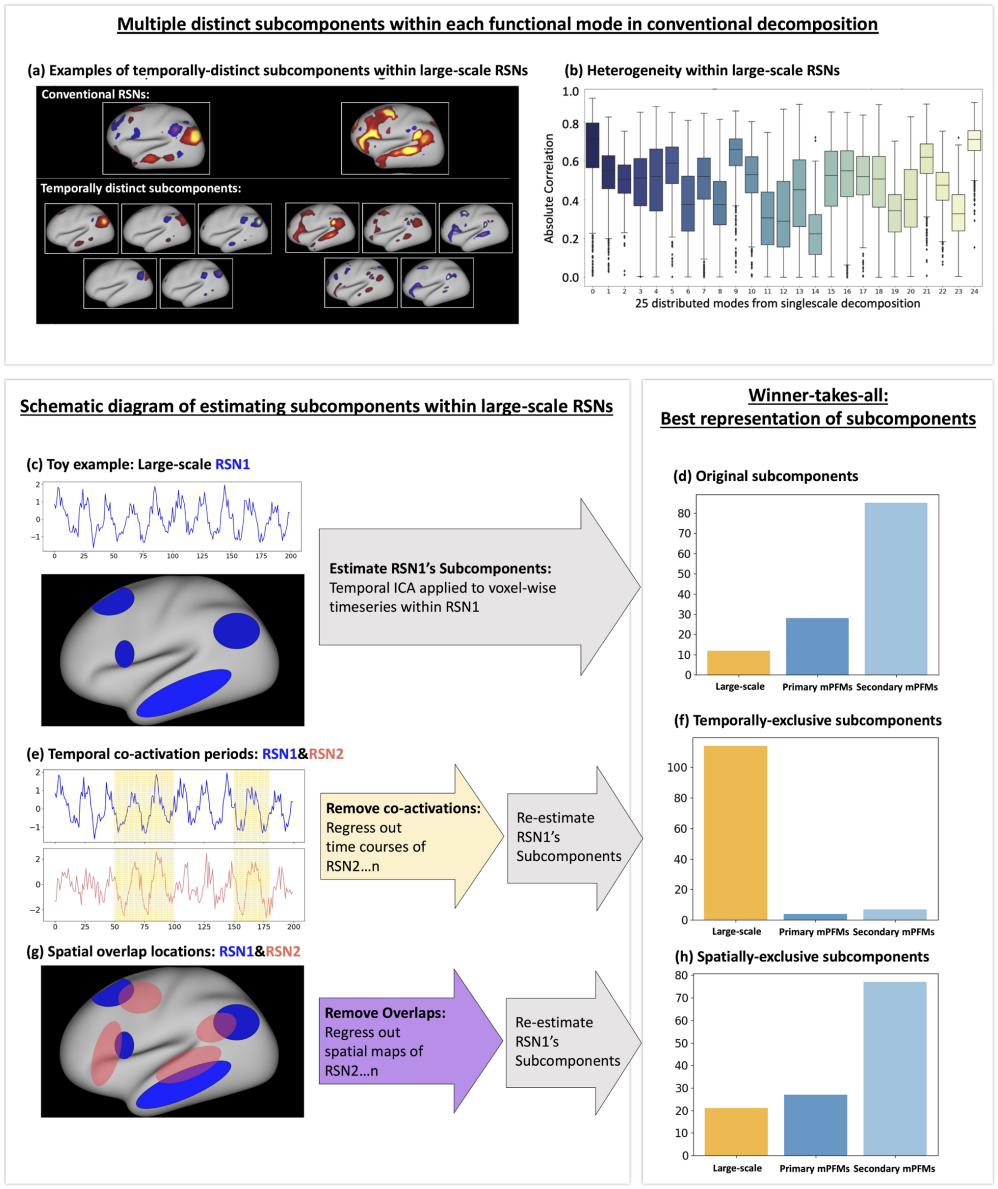
Temporally distinct subcomponents within conventional large-scale RSNs give rise to multiscale Probabilistic Functional Modes (mPFMs). A low-dimensional PFM decomposition consisting of 25 modes was estimated from cortical rfMRI data of 1003 HCP subjects, yielding conventional large-scale RSNs. Follow-up temporal ICA was applied to voxel-wise time series within each RSN to identify temporally distinct subcomponents. (a) Examples of two conventional RSNs and five subcomponents within each RSN. (b) Temporal correlation of the best-matching subcomponents to the large-scale RSNs that they originated from. (c) A schematic illustration of subcomponent estimation. (d) A winner-takes-all approach was applied, and it was found that of the 25 x 5 subcomponents, only 12 were best represented by the large-scale RSNs that they originated from, whereas 28 and 85 subcomponents were better represented by primary and by secondary mPFMs, respectively. (e) A schematic illustration of estimating temporally exclusive subcomponents. (f) A winner-takes-all approach was applied, and it was found that of the 25 x 5 temporally exclusive subcomponents, 114 (i.e., the majority) were best represented by large-scale RSNs, 4 by primary and 7 by secondary mPFMs. (g) A schematic illustration of estimating spatially exclusive subcomponents. (h) A winner-takes-all approach was applied, and it was found that of the 25 x 5 spatially exclusive subcomponents, 21 were best represented by large-scale RSNs, 27 by primary and 77 by secondary mPFMs. These results show that temporally distinct subcomponents within large-scale RSNs give rise to secondary PFMs, and that periods of temporal co-activation between subnetworks of large-scale RSNs give rise to secondary PFMs.

Next, we applied follow-up temporal ICA on data of each mode from lowD decomposition, as detailed in Section 2.3.2. Temporal ICA projects data onto statistically independent axes of variations and allows us to identify *temporally independent subcomponents* more interpretably. We restricted the number of subcomponents to 5 per mode, that is, 5 subcomponents for each of the 25 modes. Fig. 3a shows examples of these subcomponents for the default mode and language networks. We found that these subcomponents were spatially and temporally distinct, with one subcomponent being spatiotemporally best represented by the original large-scale decomposition compared with the rest of the subcomponents (Supplementary Fig. S5). Fig. 3b shows the temporal correlation of each of the best-matching subcomponents to the large-scale RSN that they originated from. Interestingly, we found that even the temporal representation of the best-represented subcomponent ranged from Pearson correlation coefficient r = 0.22 ± 0.13 to 0.70 ± 0.09 across the 25 large-scale RSNs (i.e., in many cases there is not a strong temporal match). We replicated this finding using two alternative subcomponent identification techniques, spatial ICA and PCA (Supplementary Fig. S6).

These results provide evidence that voxel-wise temporal variability within large-scale RSNs results in *temporally distinct* subcomponents that are not well represented by one time course, as assigned in conventional RSN decompositions. Instead, multiple subcomponents are needed to explain this voxel-wise temporal variability.

#### 3.1.2 Temporal subcomponents were best represented by mPFMs

We next tested whether the 5 × 25 subcomponents described in the previous subsection were better represented by mPFMs, compared with lowD large-scale RSNs alone. For this purpose, we applied a winner-takes-all approach: the time course of each of the subcomponents was correlated with: (a) large-scale RSNs, (b) primary mPFMs, and (c) secondary mPFMs. As shown in Fig. 3d, we found that only 12 of the 125 subcomponents were temporally best represented by the large-scale RSNs, 9 of which were significant (Bonferroni-corrected, Supplementary Table S2). The remaining 28 and 85 subcomponents were better represented by the primary and secondary mPFMs, respectively (28/28 and 83/85 statistically significant). Therefore, the ensemble of multiscale modes in mPFMs can capture the temporally distinct subcomponents within conventional large-scale RSNs.

#### 3.1.3 Secondary mPFMs were due to temporal co-activation periods of multiple large-scale RSNs

Resting-state networks in the brain might be spatially and temporally correlated. Therefore, it is plausible that the subcomponents that give rise to the secondary mPFMs are either due to spatially overlapping regions or due to distinct temporal co-activation periods of multiple large-scale RSNs. To test this hypothesis, we compared the original subcomponents (described in the previous subsection) with two alternative scenarios: a) temporally exclusive subcomponents: these were estimated from each large-scale RSN, after the time courses of the other 24 RSNs were regressed out of its time course; b) spatially-exclusive subcomponents: these were estimated from each large-scale RSN, after spatial maps of the other 24 RSNs were regressed out of its spatial map. Conceptual illustrations of these two analyses are included in Fig. 3e and g, respectively.

As shown in Fig. 3f, we found that a majority of the temporally exclusive subcomponents (114 out of 125, with 109 statistically significant, Supplementary Table S3) were temporally best represented by the *lowD decomposition*, unlike the original subcomponents that were best represented by mPFMs. Conversely, as shown in Fig. 3h, the majority of the spatially exclusive subcomponents (104 out of 125, 100 statistically significant, Supplementary Table S4) were instead best represented by primary and secondary mPFMs, that is, similar to original subcomponents. All results were Bonferroni corrected for multiple comparisons.

Put together, these analyses suggest that temporal co-activation periods of two or multiple large-scale RSNs from lowD decomposition give rise to new mixed-scale RSNs that are temporally distinct from the existing RSNs. These emerge as secondary mPFMs. In other words, secondary mPFMs are likely due to transient dynamic connectivity between subnetworks of large-scale modes, which are eliminated by temporal averaging (across voxels and/or time points) in low-dimensional decompositions.

#### 3.1.4 Functional connectivity modelling using mPFMs

Having examined time course modelling in mPFMs, we next investigated the functional connectivity (i.e., temporal correlations) between the modes to compare large-scale RSNs (lowD, d = 25) with mPFMs (d = 100 and 150). This was done using 4999 subjects in UK Biobank (Supplementary Fig. S7a), and separately using 1003 subjects in HCP (Supplementary Fig. S7b). Supplementary Fig. S7-right shows the histograms of between-subject consistency of functional connectivity. We observed that in both datasets, between-subject consistency was increased with dimensionality. In HCP, this was increased from 0.19 ± 0.09 for d = 25 to 0.46 ± 0.06 and 0.59 ± 0.05 for d = 100 and 150, respectively. In UK Biobank, this was increased from 0.07 ± 0.07 for d = 25 to 0.43 ± 0.13 and 0.70 ± 0.12 for d = 100 and 150, respectively. Therefore, mPFMs yielded more consistent functional connectivity values than lowD. Interestingly, subject-specific functional connectivity became sparser for mPFMs (Supplementary Fig. S7-left). This sparsity can be understood in light of the previous subcomponent analysis: by attempting to merge the time courses of multiple temporally distinct subcomponents, conventional RSNs can yield spurious connectivity between large-scale modes, which in reality can be due to one or a few of the underlying subcomponents. mPFMs capture the subcomponents using secondary modes, thus reducing such spurious connectivity, and yielding sparser subject-specific functional connectivity. As a result, functional connectivity in mPFMs becomes less contaminated by modelling inaccuracies, and more consistent across individuals.

#### 3.1.5 Cross-scale interactions of mPFMs predict individualistic traits

**Fig. 4.**
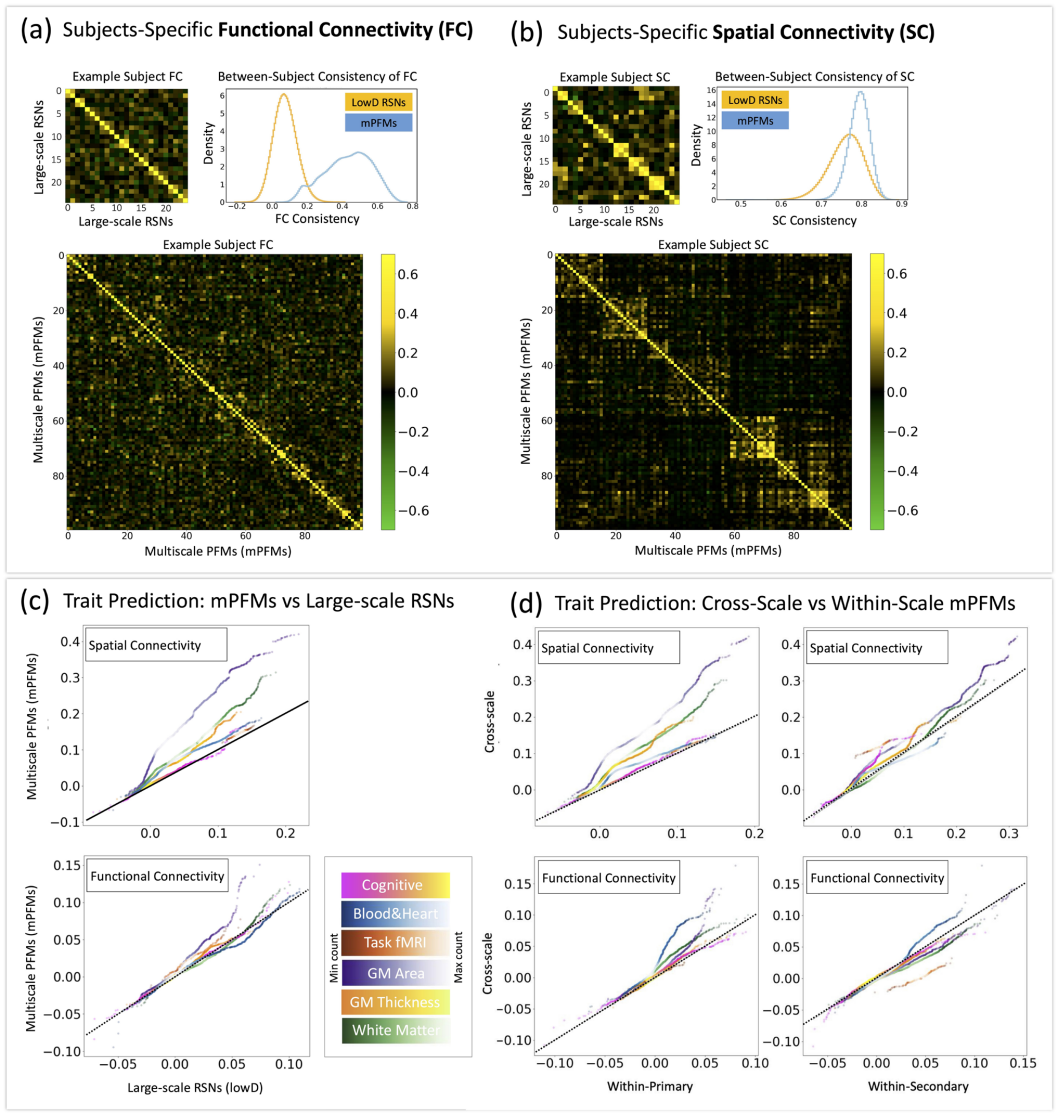
Spatiotemporal interaction of mPFMs captures subject variability that is predictive of personalised traits in UK Biobank. (a) Examples of temporal correlations (functional connectivity) between the modes, which were used as one set of features for trait prediction. Functional connectivity matrices are shown separately for conventional large-scale RSNs from lowD (25 mode) decomposition and mPFMs (highD, 100 mode) decomposition. Histograms show that between-subject consistency of functional connectivity was notably higher in mPFMs. (b) Examples of spatial connectivity between the modes which were used as another set of features for trait prediction. Spatial correlation matrices are shown separately for conventional large-scale RSNs from (lowD, 25 mode) decomposition and mPFMs (highD, 100 mode) decomposition. Histograms show that between-subject consistency of spatial connectivity was notably higher in mPFMs. (c) Comparing the prediction performance of mPFMs with large-scale RSNs for a range of 893 phenotypes across 6 categories. Spatial (top) and temporal (bottom) correlations were used as predictors. (d) Cross-scale correlations refer to spatial and temporal interactions between primary and secondary mPFMs, an aspect of the brain’s functional connectivity that conventional distributed-only RSNs or localised-only parcellations cannot capture. The prediction performance of this cross-scale spatial (top) and temporal (bottom) correlations was compared with that of within primary or within secondary modes, and a clear improvement was observed, especially in spatial domain. Density scatter plots are used to compare prediction performances, and colours denote densities, as shown in colour bars. Dotted black lines denote equal performance.

mPFMs can directly characterise the *spatiotemporal correlations between modes across multiple scales of information processing in the brain*. We evaluated the utility of temporal and spatial correlations between mPFMs for out-of-sample prediction of a range of phenotypes in UK Biobank. This was performed using volumetric rfMRI data from 4999 UK Biobank subjects and 893 phenotypes divided into 6 categories: region-wise cortical area (148 phenotypes) and thickness (148), white matter (WM) tracts’ microstructure (451), task fMRI response to the contrast of emotional faces and shapes (1), cognition (68), and cardiovascular health (77), see Section 2.6.1 for details of phenotypes, feature extraction, and prediction pipeline. Fig. 4a and b show functional and spatial connectivity of large-scale RSNs (d = 25) and mPFMs (d = 100) for an example subject. As demonstrated in these panels, cross-subject consistency of both functional and spatial connectivity was higher in mPFMs than in large-scale RSNs.

First, we compared the prediction performance of mPFMs with large-scale RSNs, shown in Fig. 4c. We found that spatial connectivity of mPFMs, after matching the number of features as elaborated in Section 2.6.4, significantly outperformed (Bonferroni corrected, Supplementary Table S5) that of large-scale decomposition in predicting all phenotype categories except cognitive scores. Similarly, functional connectivity of mPFMs significantly outperformed that of large-scale decomposition in predicting region-wise cortical area and task fMRI phenotypes. This shows that subject-specific spatiotemporal correlations between mPFMs carry additional information about each person’s traits that is otherwise missed in large-scale decomposition.

Next, we divided spatiotemporal interactions of mPFMs into three subtypes: (i) within primary mPFMs, (ii) within secondary mPFMs, and (iii) between primary and secondary mPFMs. Aiming to evaluate the added benefit of cross-scale interactions (iii), we compared its prediction performance with within-scale interactions (i.e., i and ii). As shown in Fig. 4d-top, cross-scale spatial connectivity significantly outperformed (Bonferroni corrected, Supplementary Table S5) within-secondary for region-wise cortical area and thickness, task fMRI and cognitive phenotype categories, and they additionally outperformed within-primary for all the phenotype categories, except cognitive scores. Cross-scale functional connectivity, as shown in Fig. 4d-bottom, performed on par with or worse than within-secondary, but significantly outperformed (Bonferroni corrected, Supplementary Table S5) within-primary for region-wise cortical area, thickness, cardiovascular health metrics.

These results demonstrate that spatial and temporal connectivity of the ensemble of mPFMs yields improved biomarkers for individualistic traits in two ways: first, compared with conventional large-scale RSNs, and second, subject variability captured in spatiotemporal correlations *across* multiple scales is more phenotypically relevant compared with within a single scale.

### 3.2 Validation of mPFMs

Our results so far have focussed on resolving the origins and functional relevance of mPFMs in modelling brain connectivity. Here we set to validate this new decomposition based on simulations, its reproducibility between HCP and UK Biobank datasets, and similarity to HCP’s Multimodal Parcellation.

#### 3.2.1 Simulations

**Fig. 5.**
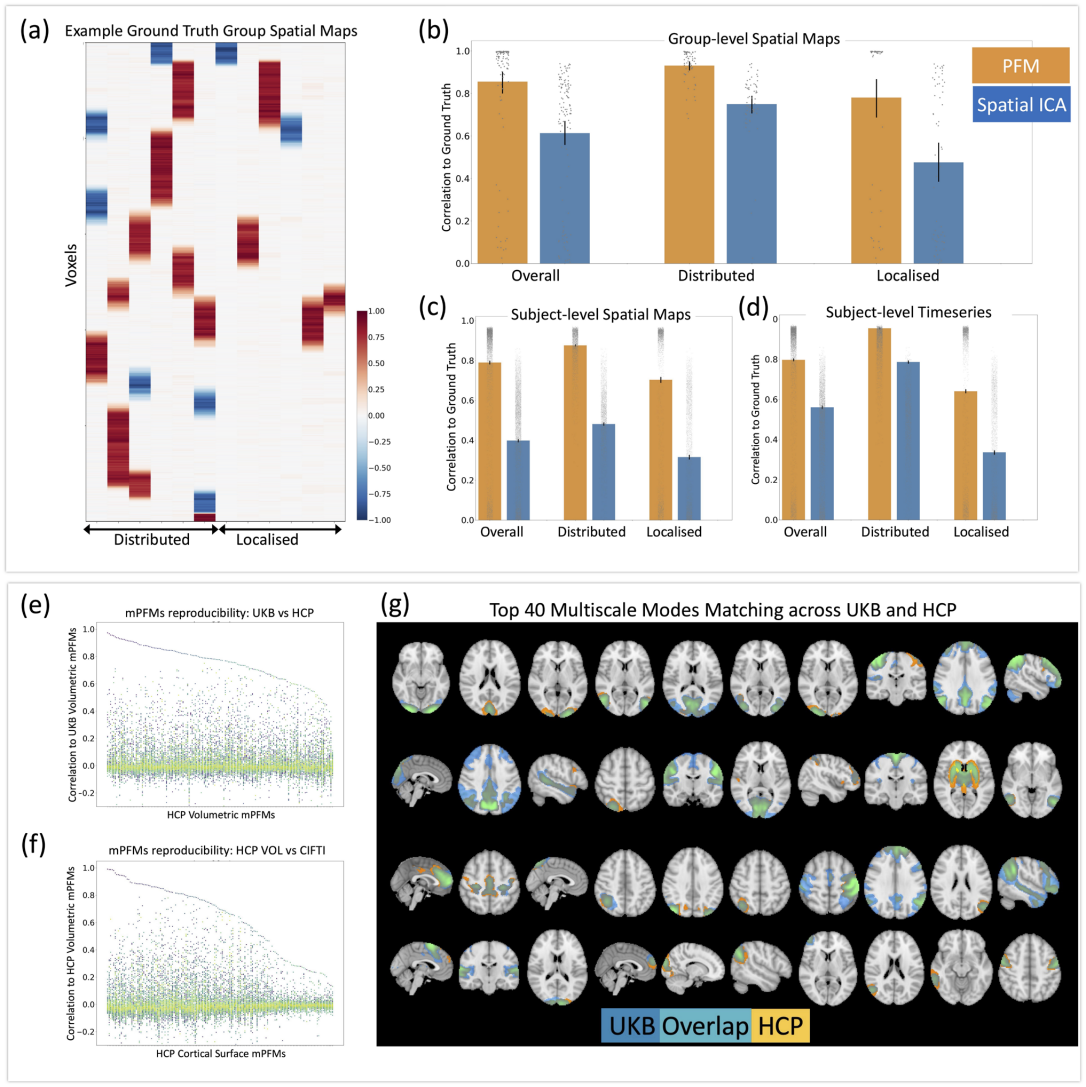
Validation of mPFMs using synthetic data (top panel) and reproducibility in real data (bottom panel). Simulations: (a) examples of simulated group-average spatial maps (1 of 10 simulated datasets). In total, 12 modes were simulated, 6 mimicking distributed modes and 6 mimicking localised subcomponents of these modes. Aiming to validate findings of Section 3.1.1, we observed that PFM decomposition can well capture: (b) group spatial maps, (c) subject spatial maps, and (d) subject time courses of these co-existing modes. The performance was ~50% higher than that of spatial ICA at group level and up to 100% higher at subject level. Real data reproducibility: PFM decomposition with 150 modes was compared at the group level between (e) UK Biobank (UKB) and HCP volumetric fMRI; each HCP mode is shown on the x axis and correlations to all UKB modes on the y axis. Clear one-to-one pairing can be seen for the top 100 modes. (f) A similar comparison was conducted between HCP volumetric and surface-based fMRI data types. (g) The top 40 matching mPFMs between HCP and UKB (both volumetric fMRI) are illustrated. HCP modes are shown in yellow, UKB in blue, and overlap in green/cyan.

Based on real data in Section 3.1, we found evidence that secondary mPFMs likely emerge to explain temporally distinct subcomponents within the conventional large-scale RSNs. In other words, we showed that the ensemble of multiscale modes captures the integrated–segregated functional brain connectivity. Here we simulated datasets, where distributed and localised modes co-existed, such that localised modes were spatial sub-modes of the distributed modes, with distinct time courses. Using these simulations, we tested how well PFM decomposition can recover the co-existence of multiscale modes, and compared its performance with spatial ICA, which is the current standard technique for RSN modelling. Across 10 simulated datasets, each included 12 modes, 6 distributed and 6 localised. Fig. 5a shows examples of group-average simulated modes in one of the datasets. We compared three elements of the estimated modes with the ground truth: group spatial maps, subject spatial maps, and subject time courses, shown in Fig. 5b–d, respectively. For the group spatial maps, the accuracy of PFM decomposition for distributed and localised modes was 0.93 and 0.78, respectively, which was reduced to 0.74 and 0.47 in spatial ICA modes. For the subject spatial maps, the accuracy of PFM decomposition for distributed and localised modes was 0.87 and 0.70, respectively, which was nearly halved (0.48 and 0.31) in spatial ICA modes. For the subject time courses, the accuracy of PFM decomposition for distributed and localised modes was 0.95 and 0.64, respectively, which was reduced to 0.78 and 0.33 in spatial ICA modes. Therefore, at both group and subject level, PFMs can recover the simulated multiscale modes with overall (average) accuracy of ~0.8 or higher.

#### 3.2.2 Reproducibility across datasets and data formats

The next step of validation involved cross-dataset reproducibility tests. We applied sPROFUMO separately to three rfMRI datasets and obtained 150 modes per run: (1) 1003 HCP subjects, volumetric whole-brain rfMRI (HCP VOL); (2) 1003 HCP subjects, CIFTI cortical rfMRI (HCP CORT); and (3) 4999 UKB subjects, volumetric whole-brain rfMRI (UKB VOL). All these scenarios yielded mPFMs consisting of primary and secondary modes. We paired these mPFMs across runs based on spatial correlation of group-level spatial maps.

Firstly, by comparing HCP VOL and UKB VOL (Fig. 5e), we found 100 modes to show very good matching (absolute correlation ≥0.7), 40 modes to show good matching (absolute correlation between 0.5 and 0.7), and 10 modes to show mediocre matching (absolute correlation ≤0.5). Some of the modes in the latter category were due to differences in structured noise across datasets (Supplementary Fig. S8). The top 40 best matching mPFMs across these datasets are shown in Fig. 5g.

Secondly, by comparing HCP VOL and HCP CORT (Fig. 5f), we found 74 modes to show very good matching (absolute correlation ≥0.7), 28 modes to show good matching (absolute correlation between 0.5 and 0.7), and 48 modes to show mediocre matching (absolute correlation ≤0.5). Some of the modes in the latter category were due to structured noise differences across data types, and the remainder were due to inherent differences between volumetric and CIFTI fMRI reconstructions.

##### 3.2.2.1 Reproducibility across multiple dimensionalities

We additionally tested for reproducibility of the mPFMs across multiple dimensionalities: 50, 100, and 150. This was done separately for UK Biobank and HCP datasets, with each dimensionality starting from a different independent initialisation. Results are shown in Supplementary Fig. S9, starting from low-dimensional d = 25 decomposition, we compared 25 versus 50, 50 versus 100, and 100 versus 150. The modes were sorted and paired based on spatial correlations between the group-level spatial maps. For the UK Biobank volumetric fMRI, we found mean ± STD of the paired correlations to be 0.90 ± 0.11 (d = 25 vs. 50), 0.84 ± 0.14 (d = 50 vs. 100), and 0.82 ± 0.14 (d = 100 vs. 150). For the HCP Cortical CIFTI fMRI, we found mean ± STD of the paired correlations to be 0.88 ± 0.08 (d = 25 vs. 50), 0.90 ± 0.11 (d = 50 vs. 100), and 0.88 ± 0.11 (d = 100 vs. 150). These strong pairings show that, as we increase the dimensionality, the existing modes at lower dimensions are largely maintained, and new modes are added to the ensemble, indicating an eventual addition of more and more secondary PFMs.

#### 3.2.3 Validating secondary mPFMs using HCP’s multi-modal parcellation

As the next step, we validated mPFMs based on the HCP’s Multimodal Parcellation v1.0 (HCP-MMP), aiming to validate the secondary mPFMs. We compared mPFMs against the HCP-MMP, which includes 360 cortical parcels. This parcellation includes both group-average and subject-specific parcels, allowing us to validate secondary mPFMs at both levels. We applied sPROFUMO to cortical rfMRI data of 446 HCP subjects (matching the number of subjects in HCP-MMP) and estimated 360 modes. Similar to before, this yielded a multiscale ensemble of modes (i.e., mPFMs).

We found an interesting group correspondence (Supplementary Fig. S10a) between secondary mPFMs and HCP-MMP, especially 95 parcel-like mPFMs showed a strong correspondence to HCP-MMP (dice similarity ≥0.5), illustrated in Supplementary Fig. S10c. In total, 73 mPFMs showed less than 0.2 dice similarity to any HCP-MMP parcels; these included conventional low-dimensional modes and the less well-known variants of distributed modes, and typically occupied multiple distant sub-regions. We further found that modes with high group-level correspondence also showed high subject-level correspondence (Supplementary Fig. S10b), with the top 95 matching modes at the group level also showing correlation of 0.47 ± 0.19 (average ± standard deviation across subjects and modes) at the subject level. These evaluations add to the previous validation steps, by demonstrating that those of the mPFMs that are parcel like have clear spatial similarities to the state-of-the-art individualised parcellations, both at the group level and the subject level.

In summary, this section on mPFM validation sheds light on robustness and interpretation of the multiscale representation of the RSNs. Specifically, in Section 3.2.1, we simulated scenarios resembling multiscale modes, that is, temporally distinct pairs of modes where one mode is a spatial sub-node of the other, thus mimicking the primary and secondary modes in real data. We showed that these can be recovered using PFM decomposition much more accurately than ICA decomposition. Next, in Section 3.2.2, we demonstrated reproducibility of mPFMs across two large-scale datasets (HCP and UKB), two standard fMRI data formats (volumetric and CIFTI), and a range of dimensionalities (25, 50, 100, 150) in both HCP and UKB. These results demonstrate that the multiscale representation is a stable finding and not a byproduct of certain data properties or choices of preprocessing or dimensionality. Finally, in Section 3.2.3, we demonstrated that secondary mPFMs can be reliably linked to the well-established HCP-MMP parcellation. Considering that the multiscale representation is (as shown earlier in Fig. 1, and Section 3.2.2.1), in principle, an ensemble of the large-scale RSNs (primary mPFMs), with the addition of the fine-grained secondary mPFMs, this analysis validated that the secondary mPFMs (especially the parcel-like) are meaningful functional entities, and not due to fMRI smoothness or noise properties.

### 3.3 mPFMs yield enhanced biomarkers of personalised traits

We have so far investigated the brain properties underlying mPFMs, and their added benefit over conventional large-scale RSNs. Here we focus on the usefulness of individual-specific mPFMs for providing biomarkers for traits and disease. This was done using two steps: 1- population covariations between mPFMs and behavioural traits in HCP and 2- out of sample prediction of phenotypes in UK Biobank. CCA and phenotype prediction results serve two distinct, complementary goals. mPFMs provide a new type of RSN representation, which is subject specific and multiscale. Therefore, we aimed to test 1- how interpretably they can be linked to non-imaging variables (CCA analysis in HCP), and 2- compared with existing techniques, how accurately they can predict non-imaging variables (phenotype prediction in UK Biobank).

#### 3.3.1 Positive–negative axis of population covariation between mPFMs and behaviour

The goal of CCA was mainly qualitative, and to illustrate how interpretably mPFMs can be linked to personalised traits. In order to achieve this, we referred to the literature and investigated whether mPFMs can capture one of the key findings in the brain–behaviour literature: a positive–negative mode of population covariation that has been discovered to link brain features to cognition and lifestyle. Positive traits such as language comprehension or life satisfaction are placed at one end of this axis, and negative traits such as antisocial behaviour or substance use at the other end. This was originally reported by Smith et al. (2015) using HCP data, and has been replicated on other large datasets since (Goyal et al., 2022).

Canonical Correlation Analysis (CCA) allows to investigate associations between a set of features from brain data and a set of personalised traits (i.e., phenotypes), all in a single integrated multivariate analysis. Using CCA, we identified modes of population co-variation between brain and phenotypes, as pairs of canonical components along which the phenotypes and mPFMs co-varied in a similar way across subjects. We used the same analysis pipeline as Smith et al. (2015), as detailed in Section 2.7.

**Fig. 6.**
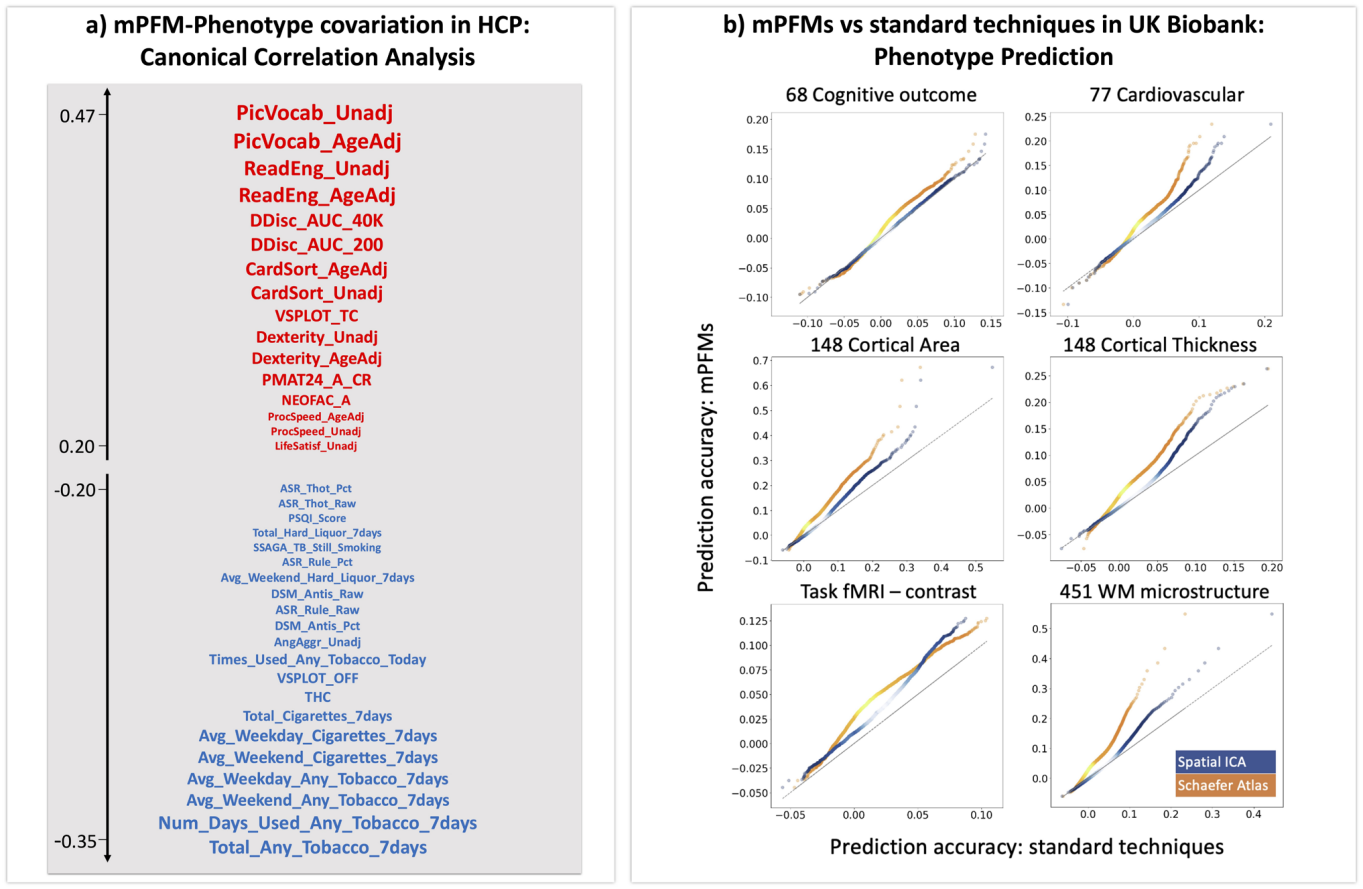
mPFMs yield biomarkers for behaviour and traits: (a) shared population covariation between mPFMs and behaviour in HCP: A positive–negative axis of population covariation identified based on top CCA components of spatial and temporal mPFMs features. (b) mPFMs yield enhanced fMRI biomarkers of traits and health compared with current standard techniques in UKB. In total, 893 phenotypes across 6 categories were used. Comparisons between trait prediction accuracies of mPFMs (y-axis) with spatial ICA and the Schaefer parcellation (x-axis blue and orange, respectively) are shown. Correlation coefficient between actual and predicted phenotypes in the test set was used as metric of accuracy. Density scatter plots are used to compare mPFM’s prediction accuracies with that of the standard techniques, each dot corresponds to the prediction accuracy of a mode for a phenotype/trait, and brighter colours (e.g., light yellow and light blue) denote higher densities. Dotted grey lines denote equal performance. The 100-mode decompositions were used for all the methods, and prediction accuracies were computed per mode, in order to obtain a more detailed summary of the modes. mPFMs outperformed the standard techniques for all the categories.

To examine the link between personalised traits and spatiotemporal characteristics of mPFMs, CCA was conducted on features related to spatial maps, spatial and functional connectivity of mPFMs. Supplementary Fig. S11a shows results of correlation between brain and phenotypes after CCA transformation. As shown in Fig. 6a, we identified the raw behavioural metrics that were most strongly linked to the top CCA components. This was done by correlating the behavioural metrics with the topmost CCA components related to spatial maps, spatial and functional connectivity, and taking the average correlation across the three. This unravelled a single axis of population variation: at one end of this axis, we found behavioural traits positively associated with the prominent CCA component. These included metrics related to cognition and emotion such as performance on language tests, executive function, self-regulation, and life satisfaction. At the other end of this axis, we found behavioural traits negatively associated with the prominent CCA component. These included metrics related to psychiatric disorders and substance use such as rule breaking, antisocial behaviour, alcohol, tobacco, and cannabis use. Therefore, mPFMs, similar to existing literature, provide a simple interpretable mapping between brain features and positive versus negative traits.

#### 3.3.2 Enhanced predictions of personalised traits in UK Biobank

Next, we evaluated the potential of mPFMs for out-of-sample prediction of traits. For this purpose, we compared mPFMs with two of the most commonly used techniques in the literature: spatial ICA, the current main RSN modelling technique in the core UK Biobank pipeline (Alfaro-Almagro et al., 2018), and the Schaefer atlas, a high-dimensional hard parcellation derived from the Yeo parcellation (Schaefer et al., 2018). For this purpose, we used the 100-dimensional Schaefer parcellation and further applied the other 2 methods to volumetric rfMRI data of 4999 UK Biobank subjects, estimating 100 mPFMs and 100 ICs. We used features of each functional mode (feature matrix, X) to make predictions about phenotypes (target, y), details in Section 2.6. We used ElasticNet regression with 5-fold nested cross-validation, and predicted 893 phenotypes divided into 6 categories: region-wise cortical area (148) and thickness (148), white matter (WM) tracts’ microstructure (451), task fMRI response to the contrast of emotional faces versus shapes (1), cognition (68) and cardiovascular health metrics (77). Correlation coefficient between actual and predicted phenotypes in the test sets was used as metric of accuracy. Results are shown in Fig. 6b, with prediction accuracies of mPFMs shown on the y-axis, and accuracies of spatial ICA modes on the x-axis. We found that mPFMs significantly outperformed (Bonferroni corrected, Supplementary Table S6) Schaefer parcellation in prediction of all the phenotype categories, and significantly outperformed spatial ICA in prediction of all the phenotype categories except cognitive traits. These results were replicated using higher dimensionalities, as shown in Supplementary Fig. S12.

These results, taken together with the findings of Section 3.1.5, demonstrate three key benefits of mPFMs over single-scale representations for phenotype predictions: firstly, the spatiotemporal connectivity between the large-scale and fine-grained PFMs (i.e., cross-scale connectivity) outperforms that of within-scale connectivity—this was illustrated using the PFM modelling framework only, since standard techniques do not yield multiscale representations; secondly, the spatiotemporal connectivity of the full multiscale representation outperforms that of lowD large-scale-only RSNs; thirdly, the full set of features obtained from the multiscale highD representation (including spatial topographies and spatiotemporal connectivity) outperforms that of standard single-scale highD representations obtained from ICA and Schaefer atlas.

## 4 Discussion

In this paper, we presented Multiscale Probabilistic Functional Modes (mPFMs), a new representation of the brain’s functional modes, that improves fMRI modelling and its utility for prediction of cognition and health. mPFMs were identified using stochastic Probabilistic Functional Modes (sPROFUMO) modelling and, unlike any existing representation, include an ensemble of modes across multiple scales. sPROFUMO does not impose or prevent this behaviour by design, instead, it allows the modes to be spatiotemporally correlated, and accumulate Bayesian evidence across individuals in large populations. Therefore, if a multiscale organisation arises from the results, this is driven by the structure in the data. Indeed, as demonstrated in the Results section, the presence of multiple distinct subcomponents within each large-scale (distributed) mode indicates spatiotemporal multiplicity within these modes, which cannot be captured by distributed-only or localised-only representations. Put together, our results demonstrate mPFMs’ potential to provide a useful representation of multiscale information processing in the brain, and enhance fMRI-derived biomarkers for traits and disease. We release this representation publicly; we have made the group-level maps openly available, whereas subject-level maps will be released in the future as part of ongoing work to rebuild a “v2” UKB pipeline (Alfaro-Almagro et al., 2018).

Multiscale modes are compatible with the notion of hierarchal information processing in the brain; that brain function is organised in a hierarchical manner, with each level of the hierarchy underlying increasingly complex and abstract processing (Cordes et al., 2002; Deco and Kringelbach, 2017; Friston, 2008; Vidaurre et al., 2017). Hierarchical processing is enabled via interactions between local unimodal information processing and distributed higher level cognitive function. Such interactions have been well established, for example, in visual search tasks, where interactions between sub-regions of the visual cortex and fronto-parietal attention networks allow for top–down modulation of visual processing by attention, as well as bottom–up modulation of attention by the incoming stimulus (Buschman & Miller, 2007; Gazzaley & Nobre, 2012). Interactions between brain systems at different levels of these hierarchies cannot be directly modelled using existing functional mode representations, considering that they can either be used to measure interactions between distributed modes (lowD decompositions) or localised regions (highD parcellations). As a result, the interactions *between* these two layers will have to be modelled using post hoc techniques, such as hierarchical clustering (Smith et al., 2013a) or module detection (Sporns & Betzel, 2016). Even then, the resulting connectivity will depend on the assumptions of the post hoc techniques, and change depending on the choice of parameters, rather than being integrated within the original brain function mapping.

In contrast, mPFM inherently incorporates such multiscale connectivity within fMRI decomposition. Crucially, as we demonstrated in the Results Section, these interactions provide stronger biomarkers of cognition and health than connectivity within large-scale or within localised modes alone. This complements previous brain–behaviour research (Bolt et al., 2018), which compared the association of individual brain regions and their respective whole-brain networks with task performance. The authors showed that for tasks such as working memory and relational, areal-level associations to task performance will become redundant when large-scale network covariation in accounted for, unlike arithmetic task, where areal-level and network-level associations showed distinctively significant roles. This study, similar to ours, highlights the need for multiscale representations.

Additionally, by allowing the modes to be spatially overlapping, mPFMs can capture functional multiplicity in the brain’s organisation, where a single region contributes to different functions depending on the context. Until recently, spatially overlapping functional modes have been largely absent from the resting-state literature. Specifically, studies have commonly relied on either spatial ICA (Beckmann et al., 2005; Calhoun & Adali, 2012), which imposes spatial independence between the modes, or hard parcellations, which enforce rigid boundaries between the modes (Craddock et al., 2012; Yeo et al., 2011). Therefore, the literature has relied on spatial bases that minimise or prevent spatial overlap. However, growing evidence is now highlighting the importance of spatial overlap in modelling cross-individual variability in brain function (Bijsterbosch et al., 2019). Importantly, recent successful characterisation of brain connectivity using gradients or connectopy mapping (Haak et al., 2018; Margulies et al., 2016) has brought functional multiplicity to the fore of resting-state connectivity. These techniques have unravelled multiple overlapping connectivity patterns within brain regions, for example, retinotopic organisation of the visual cortex, and follow-up studies have highlighted the cognitive and clinical relevance of these overlapping connectopy maps. As such, allowing for spatial overlap, in addition to and not instead of temporal correlations between the modes, is proving imperative for obtaining a more comprehensive representation of brain function. mPFMs are, on the one hand, similar to conventional spatial ICA modes, in that they capture large-scale prominent networks in the brain. One the other hand, mPFMs additionally allow multiple overlapping subcomponents within these networks to co-exist. As such, mPFMs can be viewed as a new category of brain function mapping that stands somewhere in-between conventional RSNs and the more recent gradients of connectivity. In this context, future studies would be valuable, to compare mPFMs with gradients of connectivity. Additionally, comparison with the recently introduced tensor-based decomposition methods such as NASCAR (Li et al., 2023) and Tucker (Han et al., 2024) would be valuable. Unlike spatial/temporal ICA, and similar to s/PROFUMO, these methods allow the estimation of RSNs that are both spatially and temporally correlated.

In addition to their functional relevance, mPFMs also provided enhanced biomarkers for prediction of individualistic traits in UK Biobank. To benchmark mPFMs against standard ICA-based or hard parcellation techniques, we used a range of imaging-derived and non-imaging-derived phenotypes such as cortical geometry, white matter microstructure, brain response to cognitive tasks, behavioural cognitive scores, and cardiovascular health outcome. The overall prediction accuracies of mPFMs were found to be significantly higher than standard techniques for various phenotype categories. One key reason for this higher prediction accuracy is likely the more comprehensive spatiotemporal feature set in mPFMs. More specifically, hard parcellations yield binary spatial maps for each mode/parcel, with no spatial overlaps between the modes, thus summarising individual-specific brain features solely in temporal space. Somewhat similarly, spatial ICA, due to its assumption of spatial independence between the modes, minimises spatial overlap by design (Farahibozorg et al., 2021; Kiviniemi et al., 2009; Smith et al., 2013b). Consequently, both approaches yield single-scale decompositions of functional modes. Therefore, large-scale modes, and their spatiotemporal correlation with each other and with localised modes, cannot be fully characterised by the standard techniques. Therefore, mPFMs yield a richer set of spatial and temporal features that, even after matching the number of features between the methods, can outperform the standard techniques in reflecting individualistic traits in brain function.

## Supplementary Material

Supplementary Material

## Data Availability

UK Biobank data are available upon registration and applying for data access from the UK Biobank website: http://www.ukbiobank.ac.uk/register-apply. Human Connectome Project data are available upon registration from the HCP website: https://db.humanconnectome.org. Group average mPFMs obtained from HCP and UK Biobank are available here: https://www.fmrib.ox.ac.uk/ukbiobank/PROFUMO. Code for sPROFUMO is currently available from the following repository, it will be made available in an upcoming FSL release: https://git.fmrib.ox.ac.uk/profumo/profumo/-/tree/sprofumo-cpp-clean. Code used for simulations is available from the following public repository: https://git.fmrib.ox.ac.uk/profumo/PFM_Simulations.

## References

[IMAG.a.1031-b1] Alfaro-Almagro, F., Jenkinson, M., Bangerter, N. K., Andersson, J. L. R., Griffanti, L., Douaud, G., Sotiropoulos, S. N., Jbabdi, S., Hernandez-Fernandez, M., Vallee, E., Vidaurre, D., Webster, M., McCarthy, P., Rorden, C., Daducci, A., Alexander, D. C., Zhang, H., Dragonu, I., Matthews, P. M., … Smith, S. M. (2018). Image processing and Quality Control for the first 10,000 brain imaging datasets from UK Biobank. Neuroimage, 166, 400–424. 10.1016/j.neuroimage.2017.10.034 29079522 PMC5770339

[IMAG.a.1031-b2] Alfaro-Almagro, F., McCarthy, P., Afyouni, S., Andersson, J. L. R., Bastiani, M., Miller, K. L., Nichols, T. E., & Smith, S. M. (2020). Confound modelling in UK Biobank brain imaging. Neuroimage, 224, 117002. 10.1016/j.neuroimage.2020.117002 32502668 PMC7610719

[IMAG.a.1031-b3] Andrews-Hanna, J. R., Reidler, J. S., Sepulcre, J., Poulin, R., & Buckner, R. L. (2010). Functional-anatomic fractionation of the brain’s default network. Neuron, 65, 550–562. 10.1016/j.neuron.2010.02.005 20188659 PMC2848443

[IMAG.a.1031-b4] Bastian, M., Heymann, S., & Jacomy, M. (2009). Gephi: An open source software for exploring and manipulating networks. Proc Int AAAI Conf Weblogs Soc Media, 3, 361–362. 10.1609/icwsm.v3i1.13937

[IMAG.a.1031-b5] Beckmann, C. F., DeLuca, M., Devlin, J. T., & Smith, S. M. (2005). Investigations into resting-state connectivity using independent component analysis. Philos Trans R Soc B Biol Sci, 360, 1001–1013. 10.1098/rstb.2005.1634 PMC1854918 16087444

[IMAG.a.1031-b6] Beckmann, C. F., & Smith, S. M. (2004). Probabilistic independent component analysis for functional magnetic resonance imaging. IEEE Trans Med Imaging, 23, 137–152. 10.1109/TMI.2003.822821 14964560

[IMAG.a.1031-b7] Bellec, P., Rosa-Neto, P., Lyttelton, O. C., Benali, H., & Evans, A. C. (2010). Multi-level bootstrap analysis of stable clusters in resting-state fMRI. Neuroimage, 51, 1126–1139. 10.1016/j.neuroimage.2010.02.082 20226257

[IMAG.a.1031-b8] Bijsterbosch, J. D., Beckmann, C. F., Woolrich, M. W., Smith, S. M., & Harrison, S. J. (2019). The relationship between spatial configuration and functional connectivity of brain regions revisited. Elife, 8, e44890. 10.7554/eLife.44890 31066676 PMC6541435

[IMAG.a.1031-b9] Biswal, B., Zerrin Yetkin, F., Haughton, V. M., & Hyde, J. S. (1995). Functional connectivity in the motor cortex of resting human brain using echo‐planar MRI. Magn Reson Med, 34, 537–541. 10.1002/mrm.1910340409 8524021

[IMAG.a.1031-b10] Bolt, T., Prince, E. B., Nomi, J. S., Messinger, D., Llabre, M. M., & Uddin, L. Q. (2018). Combining region- and network-level brain-behavior relationships in a structural equation model. Neuroimage, 165, 158–169. 10.1016/j.neuroimage.2017.10.007 29030103

[IMAG.a.1031-b11] Buckner, R. L., & Vincent, J. L. (2007). Unrest at rest: Default activity and spontaneous network correlations. Neuroimage, 37, 1091–1096. 10.1016/j.neuroimage.2007.01.010 17368915

[IMAG.a.1031-b12] Buschman, T. J., & Miller, E. K. (2007). Top-down versus bottom-up control of attention in the prefrontal and posterior parietal cortices. Science, 315, 1860–1862. 10.1126/science.1138071 17395832

[IMAG.a.1031-b13] Calhoun, V. D., & Adali, T. (2012). Multisubject independent component analysis of fMRI: A decade of intrinsic networks, default mode, and neurodiagnostic discovery. IEEE Rev Biomed Eng, 5, 60–73. 10.1109/RBME.2012.2211076 23231989 PMC4433055

[IMAG.a.1031-b14] Calhoun, V. D., Adali, T., Pearlson, G. D., & Pekar, J. J. (2001). A method for making group inferences from functional MRI data using independent component analysis. Hum Brain Mapp, 14, 140–151. 10.1002/hbm.1048 11559959 PMC6871952

[IMAG.a.1031-b15] Calhoun, V. D., Kiehl, K. A., & Pearlson, G. D. (2008). Modulation of temporally coherent brain networks estimated using ICA at rest and during cognitive tasks. Hum Brain Mapp, 29, 828–838. 10.1002/hbm.20581 18438867 PMC2649823

[IMAG.a.1031-b16] Cordes, D., Haughton, V., Carew, J. D., Arfanakis, K., & Maravilla, K. (2002). Hierarchical clustering to measure connectivity in fMRI resting-state data. Magn Reson Imaging, 20, 305–317. 10.1016/S0730-725X(02)00503-9 12165349

[IMAG.a.1031-b17] Craddock, R. C., James, G. A., Holtzheimer, P. E., Hu, X. P., & Mayberg, H. S. (2012). A whole brain fMRI atlas generated via spatially constrained spectral clustering. Hum Brain Mapp, 33, 1914–1928. 10.1002/hbm.21333 21769991 PMC3838923

[IMAG.a.1031-b18] Dadi, K., Varoquaux, G., Machlouzarides-Shalit, A., Gorgolewski, K. J., Wassermann, D., Thirion, B., & Mensch, A. (2020). Fine-grain atlases of functional modes for fMRI analysis. Neuroimage, 221, 117126. 10.1016/j.neuroimage.2020.117126 32673748

[IMAG.a.1031-b19] Damoiseaux, J. S., Rombouts, S. A. R. B., Barkhof, F., Scheltens, P., Stam, C. J., Smith, S. M., & Beckmann, C. F. (2006). Consistent resting-state networks across healthy subjects. Proc Natl Acad Sci U S A, 103, 13848–13853. 10.1073/pnas.0601417103 16945915 PMC1564249

[IMAG.a.1031-b20] Deco, G., & Kringelbach, M. L. (2017). Hierarchy of information processing in the brain: A novel ‘intrinsic ignition’ framework. Neuron, 94, 961–968. 10.1016/j.neuron.2017.03.028 28595052

[IMAG.a.1031-b21] Elliott, L. T., Sharp, K., Alfaro-Almagro, F., Shi, S., Miller, K. L., Douaud, G., Marchini, J., & Smith, S. M. (2018). Genome-wide association studies of brain imaging phenotypes in UK Biobank. Nature, 562, 210–216. 10.1038/s41586-018-0571-7 30305740 PMC6786974

[IMAG.a.1031-b22] Farahibozorg, S.-R., Bijsterbosch, J. D., Gong, W., Jbabdi, S., Smith, S. M., Harrison, S. J., & Woolrich, M. W. (2021). Hierarchical modelling of functional brain networks in population and individuals from big fMRI data. Neuroimage, 243, 118513. 10.1016/j.neuroimage.2021.118513 34450262 PMC8526871

[IMAG.a.1031-b23] Finn, E. S., Shen, X., Scheinost, D., Rosenberg, M. D., Huang, J., Chun, M. M., Papademetris, X., & Constable, R. T. (2015). Functional connectome fingerprinting: Identifying individuals using patterns of brain connectivity. Nat Neurosci, 18, 1664–1671. 10.1038/nn.4135 26457551 PMC5008686

[IMAG.a.1031-b24] Friston, K. (2008). Hierarchical models in the brain. PLoS Comput Biol, 4, e1000211. 10.1371/journal.pcbi.1000211 18989391 PMC2570625

[IMAG.a.1031-b25] Gazzaley, A., & Nobre, A. C. (2012). Top-down modulation: Bridging selective attention and working memory. Trends Cogn Sci, 16, 129–135. 10.1016/j.tics.2011.11.014 22209601 PMC3510782

[IMAG.a.1031-b26] Glasser, M. F., Coalson, T. S., Robinson, E. C., Hacker, C. D., Harwell, J., Yacoub, E., Ugurbil, K., Andersson, J., Beckmann, C. F., Jenkinson, M., Smith, S. M., & Van Essen, D. C. (2016). A multi-modal parcellation of human cerebral cortex. Nature, 536, 171–178. 10.1038/nature18933 27437579 PMC4990127

[IMAG.a.1031-b27] Glasser, M. F., Sotiropoulos, S. N., Wilson, J. A., Coalson, T. S., Fischl, B., Andersson, J. L., Xu, J., Jbabdi, S., Webster, M., Polimeni, J. R., Van Essen, D. C., & Jenkinson, M. (2013). The minimal preprocessing pipelines for the Human Connectome Project. Neuroimage, 80, 105–124. 10.1016/j.neuroimage.2013.04.127 23668970 PMC3720813

[IMAG.a.1031-b28] Gong, W., Beckmann, C. F., & Smith, S. M. (2021). Phenotype discovery from population brain imaging. Med Image Anal, 71, 102050. 10.1016/j.media.2021.102050 33905882 PMC8850869

[IMAG.a.1031-b29] Goyal, N., Moraczewski, D., Bandettini, P. A., Finn, E. S., & Thomas, A. G. (2022). The positive-negative mode link between brain connectivity, demographics and behaviour: A pre-registered replication of Smith et al. (2015). R Soc Open Sci, 9(2), 201090. 10.1098/rsos.201090 35186306 PMC8847886

[IMAG.a.1031-b30] Groves, A. R., Beckmann, C. F., Smith, S. M., & Woolrich, M. W. (2011). Linked independent component analysis for multimodal data fusion. Neuroimage, 54, 2198–2217. 10.1016/j.neuroimage.2010.09.073 20932919

[IMAG.a.1031-b31] Haak, K. V., Marquand, A. F., & Beckmann, C. F. (2018). Connectopic mapping with resting-state fMRI. Neuroimage, 170, 83–94. 10.1016/j.neuroimage.2017.06.075 28666880

[IMAG.a.1031-b32] Han, Y., Lin, Q.-H., Kuang, L.-D., Zhao, B.-H., Gong, X.-F., Cong, F., Wang, Y.-P., & Calhoun, V. D. (2024). A core tensor sparsity enhancement method for solving Tucker-2 model of multi-subject fMRI data. Biomed Signal Process Control, 95, 106471. 10.1016/j.bspc.2024.106471

[IMAG.a.1031-b33] Harrison, S. J., Bijsterbosch, J. D., Segerdahl, A. R., Fitzgibbon, S. P., Farahibozorg, S.-R., Duff, E., Smith, S. M., & Woolrich, M. W. (2020). Modelling subject variability in the spatial and temporal characteristics of functional modes. Neuroimage, 222, 117226. 10.1016/j.neuroimage.2020.117226 32771617 PMC7779373

[IMAG.a.1031-b34] Herculano-Houzel, S. (2009). The human brain in numbers: A linearly scaled-up primate brain. Front Hum Neurosci, 3, 31. 10.3389/neuro.09.031.2009 19915731 PMC2776484

[IMAG.a.1031-b35] Jiang, R., Calhoun, V. D., Fan, L., Zuo, N., Jung, R., Qi, S., Lin, D., Li, J., Zhuo, C., Song, M., Fu, Z., Jiang, T., & Sui, J. (2020). Gender differences in connectome-based predictions of individualized intelligence quotient and sub-domain scores. Cerebral Cortex, 30, 888–900. 10.1093/cercor/bhz134 31364696 PMC7132922

[IMAG.a.1031-b36] Kiviniemi, V., Starck, T., Remes, J., Long, X., Nikkinen, J., Haapea, M., Veijola, J., Moilanen, I., Isohanni, M., Zang, Y. F., & Tervonen, O. (2009). Functional segmentation of the brain cortex using high model order group PICA. Hum Brain Mapp, 30, 3865–3886. 10.1002/hbm.20813 19507160 PMC6870574

[IMAG.a.1031-b37] Kong, R., Li, J., Orban, C., Sabuncu, M. R., Liu, H., Schaefer, A., Sun, N., Zuo, X. N., Holmes, A. J., Eickhoff, S. B., & Yeo, B. T. T. (2019). Spatial topography of individual-specific cortical networks predicts human cognition, personality, and emotion. Cerebral Cortex, 29, 2533–2551. 10.1093/cercor/bhy123 29878084 PMC6519695

[IMAG.a.1031-b38] Li, J., Liu, Y., Wisnowski, J. L., & Leahy, R. M. (2023). Identification of overlapping and interacting networks reveals intrinsic spatiotemporal organization of the human brain. Neuroimage, 270, 119944. 10.1016/j.neuroimage.2023.119944 36801371 PMC10092006

[IMAG.a.1031-b39] Lipkin, B., Tuckute, G., Affourtit, J., Small, H., Mineroff, Z., Kean, H., Jouravlev, O., Rakocevic, L., Pritchett, B., Siegelman, M., Hoeflin, C., Pongos, A., Blank, I. A., Struhl, M. K., Ivanova, A., Shannon, S., Sathe, A., Hoffmann, M., Nieto-Castañón, A., & Fedorenko, E. (2022). Probabilistic atlas for the language network based on precision fMRI data from &gt;800 individuals. Sci Data, 9, 529. 10.1038/s41597-022-01645-3 36038572 PMC9424256

[IMAG.a.1031-b40] Mairal, J., Bach, F., Ponce, J., & Sapiro, G. (2010). Online learning for matrix factorization and sparse coding. J Mach Learn Res, 11, 19–60. 10.1145/1553374.1553463

[IMAG.a.1031-b41] Margulies, D. S., Ghosh, S. S., Goulas, A., Falkiewicz, M., Huntenburg, J. M., Langs, G., Bezgin, G., Eickhoff, S. B., Castellanos, F. X., Petrides, M., Jefferies, E., & Smallwood, J. (2016). Situating the default-mode network along a principal gradient of macroscale cortical organization. Proc Natl Acad Sci U S A, 113, 12574–12579. 10.1073/pnas.1608282113 27791099 PMC5098630

[IMAG.a.1031-b42] McKeown, M. J., Makeig, S., Brown, G. G., Jung, T. P., Kindermann, S. S., Bell, A. J., & Sejnowski, T. J. (1998). Analysis of fMRI data by blind separation into independent spatial components. Hum Brain Mapp, 6, 160–188. 10.1002/(SICI)1097-0193(1998)6:3<160::AID-HBM5>3.0.CO;2-1 9673671 PMC6873377

[IMAG.a.1031-b43] Pedregosa, F., Varoquaux, G., Gramfort, A., Michel, V., Thirion, B., Grisel, O., Blondel, M., Prettenhofer, P., Weiss, R., Dubourg, V., Vanderplas, J., Passos, A., Cournapeau, D., Brucher, M., Perrot, M., & Duchesnay, É. (2011). Scikit-learn: Machine learning in python. J Mach Learn Res, 12, 2825–2830. 10.3389/fninf.2014.00014

[IMAG.a.1031-b44] Pervaiz, U., Vidaurre, D., Woolrich, M. W., & Smith, S. M. (2020). Optimising network modelling methods for fMRI. Neuroimage, 211, 116604. 10.1016/j.neuroimage.2020.116604 32062083 PMC7086233

[IMAG.a.1031-b45] Power, J. D., Cohen, A. L., Nelson, S. M., Wig, G. S., Barnes, K. A., Church, J. A., Vogel, A. C., Laumann, T. O., Miezin, F. M., Schlaggar, B. L., & Petersen, S. E. (2011). Functional network organization of the human brain. Neuron, 72(4), 665–678. 10.1016/j.neuron.2011.09.006 22099467 PMC3222858

[IMAG.a.1031-b46] Raichle, M. E., MacLeod, A. M., Snyder, A. Z., Powers, W. J., Gusnard, D. A., & Shulman, G. L. (2001). A default mode of brain function. Proc Natl Acad Sci U S A, 98, 676–682. 10.1073/pnas.98.2.676 11209064 PMC14647

[IMAG.a.1031-b47] Robinson, E. C., Jbabdi, S., Glasser, M. F., Andersson, J., Burgess, G. C., Harms, M. P., Smith, S. M., Van Essen, D. C., & Jenkinson, M. (2014). MSM: A new flexible framework for multimodal surface matching. Neuroimage, 100, 414–426. 10.1016/j.neuroimage.2014.05.069 24939340 PMC4190319

[IMAG.a.1031-b48] Schaefer, A., Kong, R., Gordon, E. M., Laumann, T. O., Zuo, X. N., Holmes, A. J., Eickhoff, S. B., & Yeo, B. T. T. (2018). Local-global parcellation of the human cerebral cortex from intrinsic functional connectivity MRI. Cerebral Cortex, 28, 3095–3114. 10.1093/cercor/bhx17 28981612 PMC6095216

[IMAG.a.1031-b49] Smith, S. M., Beckmann, C. F., Andersson, J., Auerbach, E. J., Bijsterbosch, J., Douaud, G., Duff, E., Feinberg, D. A., Griffanti, L., Harms, M. P., Kelly, M., Laumann, T., Miller, K. L., Moeller, S., Petersen, S., Power, J., Salimi-Khorshidi, G., Snyder, A. Z., Vu, A. T., … Glasser, M. F. (2013a). Resting-state fMRI in the Human Connectome Project. Neuroimage, 80, 144–168. 10.1016/j.neuroimage.2013.05.039 23702415 PMC3720828

[IMAG.a.1031-b50] Smith, S. M., Fox, P. T., Miller, K. L., Glahn, D. C., Fox, P. M., Mackay, C. E., Filippini, N., Watkins, K. E., Toro, R., Laird, A. R., & Beckmann, C. F. (2009). Correspondence of the brain’s functional architecture during activation and rest. Proc Natl Acad Sci U S A, 106, 13040–13045. 10.1073/pnas.0905267106 19620724 PMC2722273

[IMAG.a.1031-b51] Smith, S. M., Hyvärinen, A., Varoquaux, G., Miller, K. L., & Beckmann, C. F. (2014). Group-PCA for very large fMRI datasets. Neuroimage, 101, 738–749. 10.1016/j.neuroimage.2014.07.051 25094018 PMC4289914

[IMAG.a.1031-b52] Smith, S. M., Nichols, T. E., Vidaurre, D., Winkler, A. M., Behrens, T. E. J., Glasser, M. F., Ugurbil, K., Barch, D. M., Van Essen, D. C., & Miller, K. L. (2015). A positive-negative mode of population covariation links brain connectivity, demographics and behavior. Nat Neurosci, 18(11), 1565–1567. 10.1038/nn.4125 26414616 PMC4625579

[IMAG.a.1031-b53] Smith, S. M., Vidaurre, D., Beckmann, C. F., Glasser, M. F., Jenkinson, M., Miller, K. L., Nichols, T. E., Robinson, E. C., Salimi-Khorshidi, G., Woolrich, M. W., Barch, D. M., Uǧurbil, K., & Van Essen, D. C. (2013b). Functional connectomics from resting-state fMRI. Trends Cogn Sci, 17(12), 666–682. 10.1016/j.tics.2013.09.016 24238796 PMC4004765

[IMAG.a.1031-b54] Snoek, L., Miletić, S., & Scholte, H. S. (2019). How to control for confounds in decoding analyses of neuroimaging data. Neuroimage, 184, 741–760. 10.1016/j.neuroimage.2018.09.074 30268846

[IMAG.a.1031-b55] Sporns, O., & Betzel, R. F. (2016). Modular brain networks. Annu Rev Psychol, 67, 613–640. 10.1146/annurev-psych-122414-033634 26393868 PMC4782188

[IMAG.a.1031-b56] Vidaurre, D., Smith, S. M., & Woolrich, M. W. (2017). Brain network dynamics are hierarchically organized in time. Proc Natl Acad Sci USA, 114, 201705120. 10.1073/pnas.1705120114 PMC5715736 29087305

[IMAG.a.1031-b57] Winkler, A. M., Webster, M. A., Vidaurre, D., Nichols, T. E., & Smith, S. M. (2015). Multi-level block permutation. Neuroimage, 123, 253–268. 10.1016/j.neuroimage.2015.05.092 26074200 PMC4644991

[IMAG.a.1031-b58] Woolrich, M. W., Behrens, T. E. J., & Smith, S. M. (2004). Constrained linear basis sets for HRF modelling using Variational Bayes. Neuroimage, 21, 1748–1761. 10.1016/j.neuroimage.2003.12.024 15050595

[IMAG.a.1031-b59] Yeo, B. T. T., Krienen, F. M., Sepulcre, J., Sabuncu, M. R., Lashkari, D., Hollinshead, M., Roffman, J. L., Smoller, J. W., Zöllei, L., Polimeni, J. R., Fisch, B., Liu, H., & Buckner, R. L. (2011). The organization of the human cerebral cortex estimated by intrinsic functional connectivity. J Neurophysiol, 106, 1125–1165. 10.1152/jn.00338.2011 21653723 PMC3174820

