# Supplementary material for: Multiscale modes of functional brain connectivity
Source: Imaging Neurosci (Camb). 2025 Dec 10;3:IMAG.a.1031. doi: 10.1162/IMAG.a.1031 (PMC12696669; doi:10.1162/IMAG.a.1031)
Supplement: Supplementary Material [file IMAG.a.1031_supp.pdf]

# Supplementary Materials

## Multiscale Modes of Functional Brain Connectivity


**Authors**

S Rezvan Farahibozorg,[1]* Samuel J Harrison,[1] Janine D Bijsterbosch,[2] Mark W Woolrich,[1,3] Stephen M Smith,[1]

**Affiliations**

[1]FMRIB, Oxford Centre for Integrative Neuroimaging, Nuffield Dept. of Clinical Neuroscience, Oxford University, Oxford, UK.

[2]Department of Radiology, Washington University in St Louis, St. Louis, USA.

[3]OHBA, Oxford Centre for Integrative Neuroimaging, Department of Psychiatry, Oxford University, Oxford, UK.

*Correspondence to: rezvan.farahibozorg@ndcn.ox.ac.uk

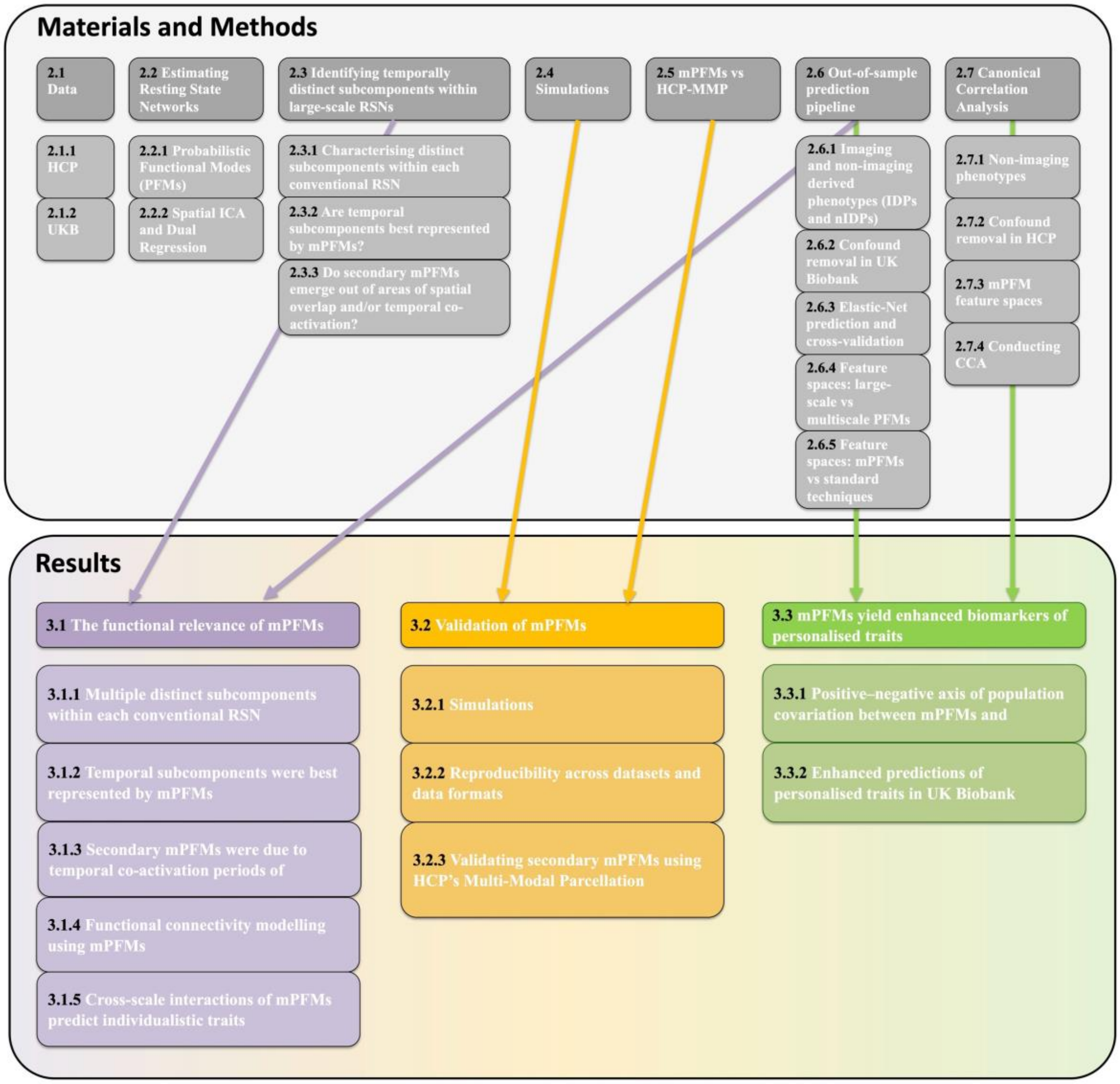


**Figure S 1 A Flowchart of different subsections of Methods and Results**, as well as the key analysis steps feeding into each Results subsection. Note that since Data and Estimating RSNs feed into multiple Results sections, they are not linked via arrows to a void unnecessary business of the graph.

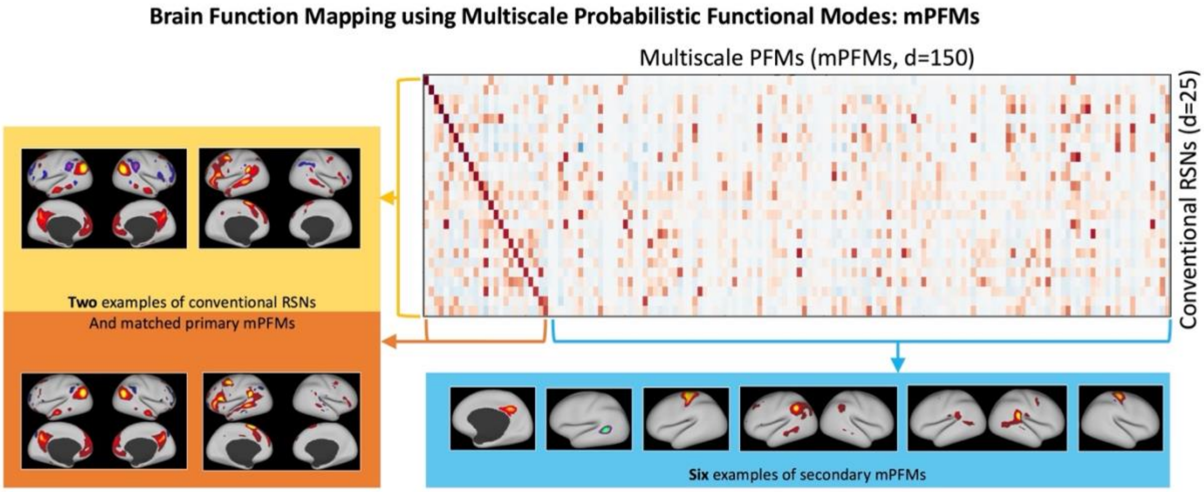


**Figure S 2 Supplement to Figure 1**: This figure provides an alternative visualisation of the comparison between multiscale Probabilistic Functional Modes (mPFMs) and conventional large-scale RSNs. This visualisation provides additional clarification that when mPFMs are spatially paired with large-scale RSNs from 25-mode decomposition (yellow), 25 of the mPFMs show a clear one-to-one matching, labelled as Primary mPFMs (orange). Secondary mPFMs (blue) are the remaining 125 mPFMs, that start appearing with increased dimensionality.

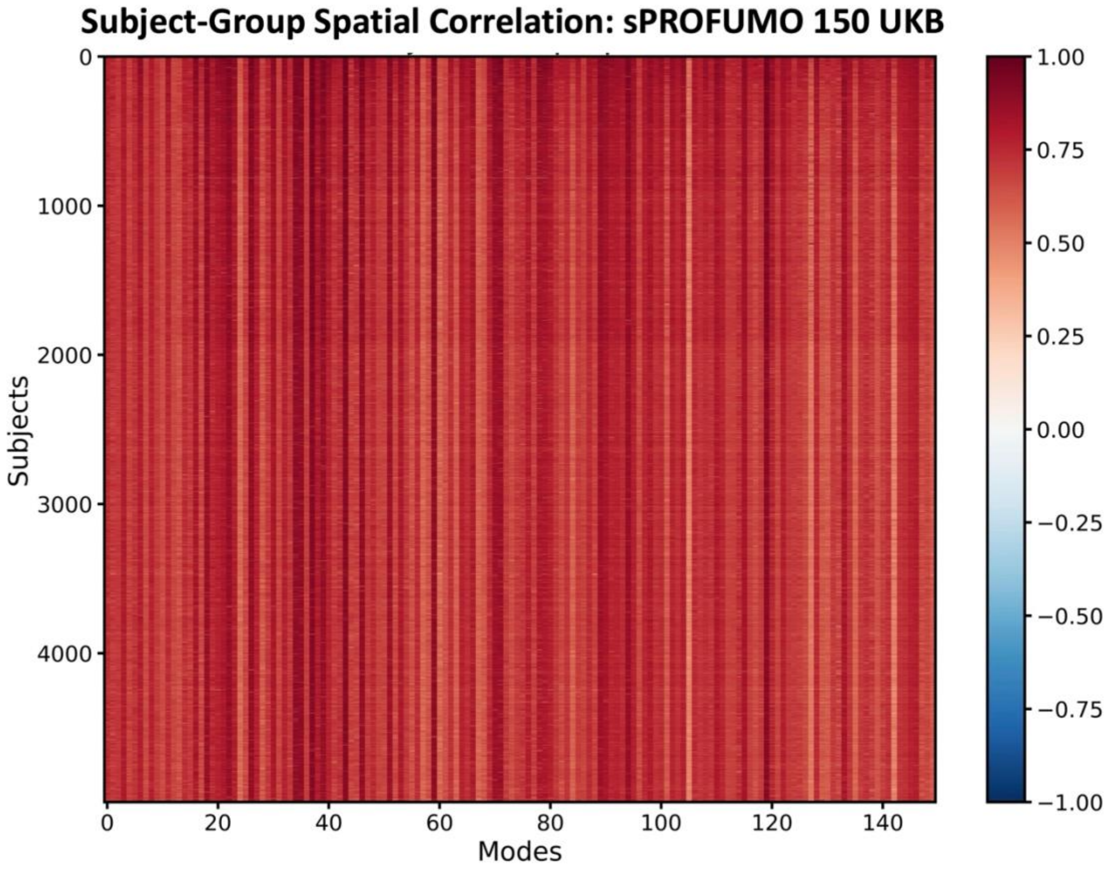


**Figure S 3 An example of Subject-group similarity of spatial maps obtained from 150-PFM decomposition of rfMRI data from 5000 subjects in UK Biobank (UKB).** This can be used to examine which subject-PFM pairs have been reliably estimated. Bad subject-PFMs can be identified using two heuristic criteria: firstly, if the spatial correlation to the group is very small (<0.1), this indicates a very noisy estimation of that specific PFM for that subject – which would be worth considering removal. Secondly, if the spatial correlation is artefactually high (>0.99), this indicates that posteriors were not reliably estimable at subject level, and therefore that subject-PFM was reverted to group priors – such modes should be removed. In general, in the example shown here, all the modes were estimated reliably for all the subjects. It is worth noting that some PFMs are generally of higher SNR (e.g., Visual or Motor), and they will be more consistent across subjects (columns with darker red colours), whereas other lower-SNR PFMs will be less consistent across subjects (columns with lighter red colours).

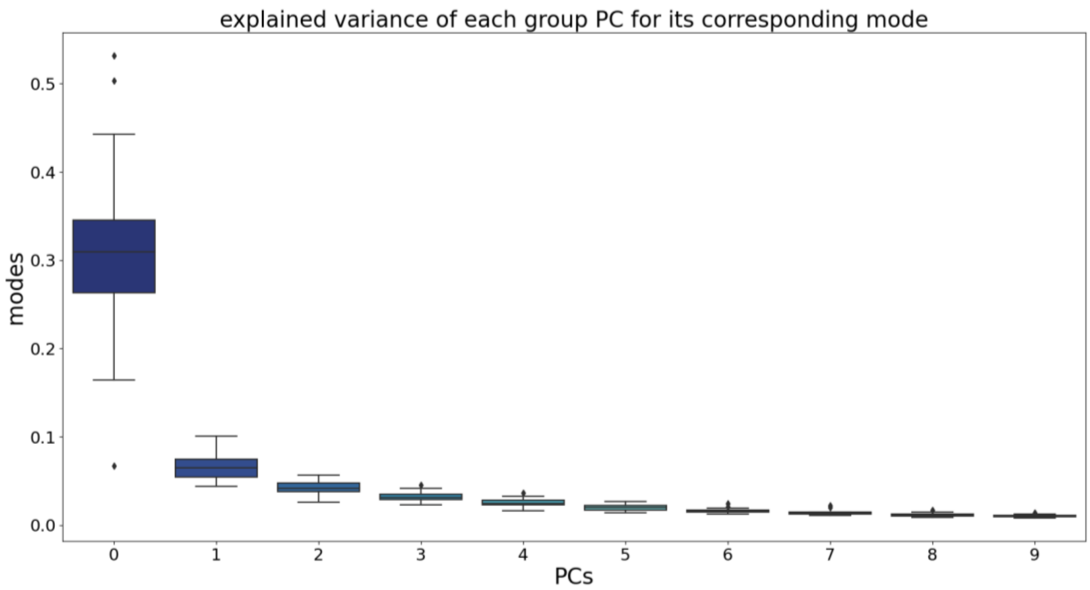


**Figure S 4 Supplement to Section 3.1.1:** Principal Component Analysis was used to test if one temporal component is sufficient to capture temporal variability within conventional large-scale RSNs from lowD decompositions of rfMRI. LowD refers to 25 modes obtained from PFM decomposition of rfMRI in HCP. The top 10 PCs per lowD mode are illustrated on the x-axis, and their explained variance on the y-axis. Boxplots show median and confidence intervals across 25 modes.

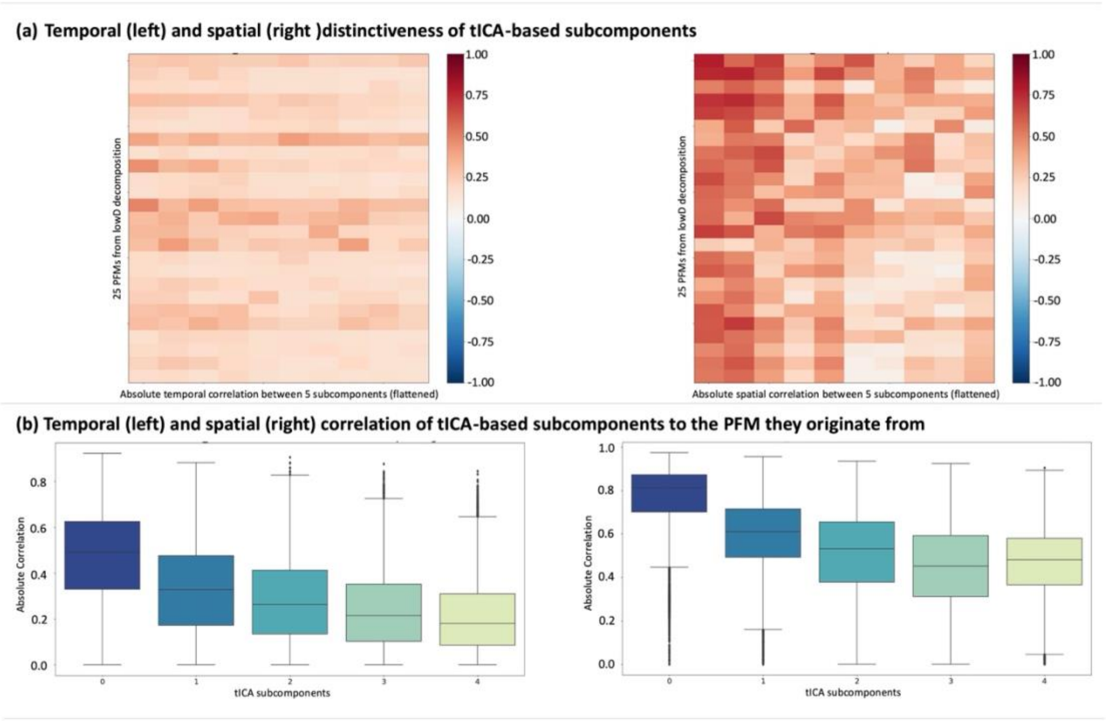


**Figure S 5 Supplement to section 3.1.1:** a) The degree to which 5x25 subcomponents estimated using temporal ICA are spatially (right) and temporally (left) correlated with each other (i.e. testing for distinctiveness). The 25 rows correspond to the 25 modes, and the flattened above-diagonal of 5x5 correlation matrices (correlations between subcomponents estimated within-subject and then averaged over subjects) appear on the x-axis. b) Temporal (left) and spatial (right) correlation of 5x25 subcomponents with their respective lowD modes. Boxplots show median and confidence intervals across 25 modes.

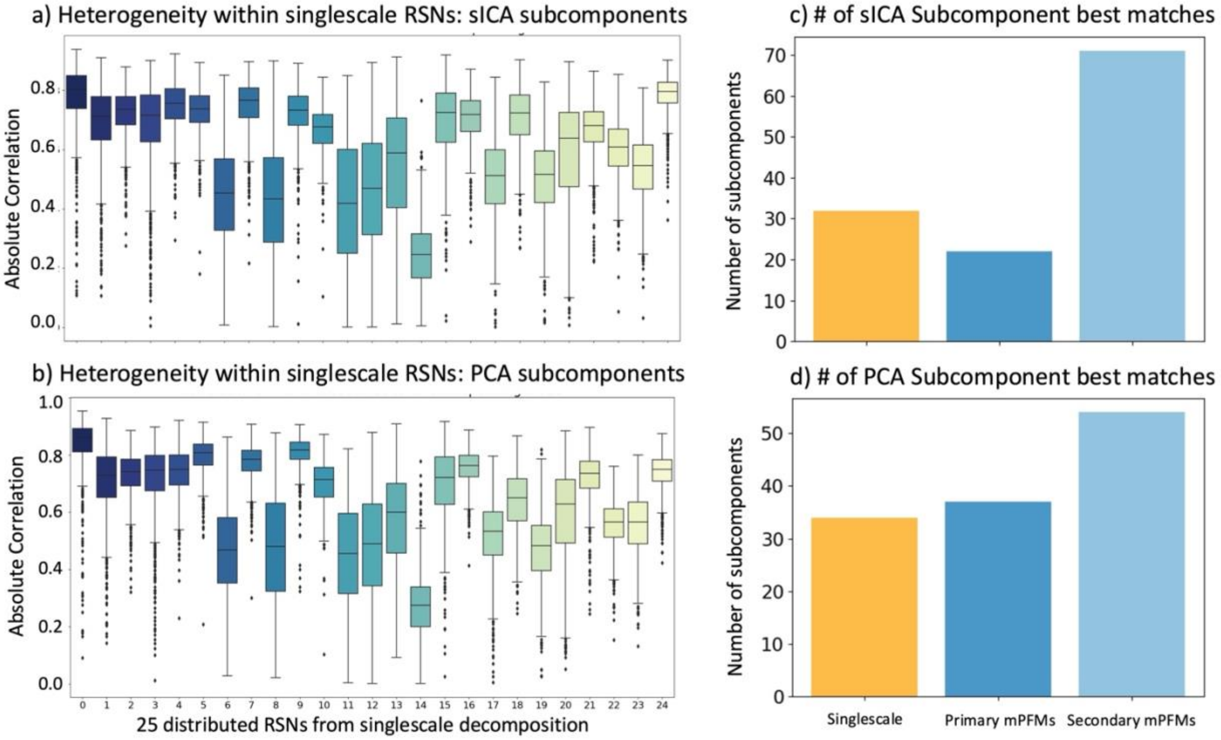


**Figure S 6 Supplement to Section 3.1.1:** A low-dimensional (lowD) PFM decomposition consisting of 25 modes was estimated from cortical rfMRI data of 1003 HCP subjects, yielding conventional large-scale RSNs, and follow-up spatial ICA (top) and PCA (bottom) applied to voxel-wise timeseries within these RSNs to identify temporally-distinct subcomponents. This analysis was conducted to confirm that subcomponent identification results in section 3.1.1 are not specific to the choice of temporal ICA as the subcomponent identification technique. Temporal correlation of the best-matching a) sICA and b) PCA subcomponents to the large-scale RSNs that they originated from; A winner-takes-all approach was applied: c) of the 25x5 sICA subcomponents 32, 22 and 71 were best represented by large-scale decomposition, primary and secondary mPFMs, respectively; d) of the 25x5 PCA subcomponents 34, 37 and 54 were best represented by large-scale decomposition, primary and secondary mPFMs, respectively.

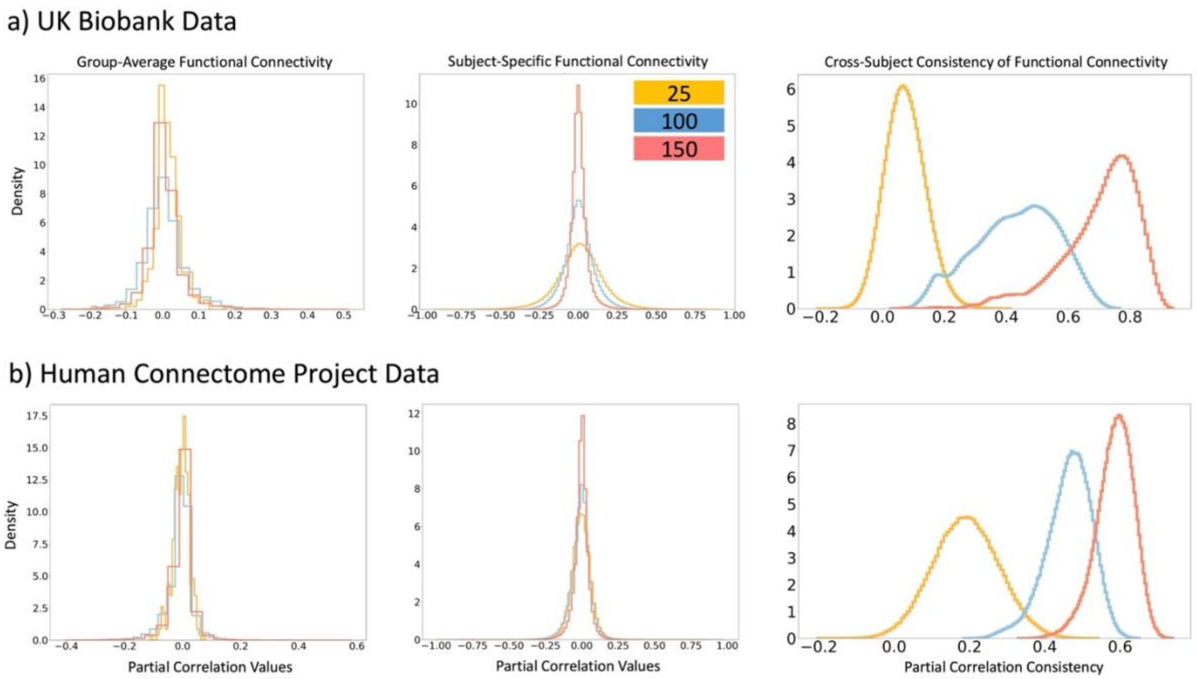


**Figure S 7 Supplement to section 3.1.4:** Functional connectivity values between modes, estimated using regularised partial correlations. Three PFM dimensionalities are compared: 25, 100 and 150 based on a) 4999 subjects in UK Biobank data (volumetric fMRI) and b) 1003 subjects in Human Connectome Project data (cortical CIFTI). Left: distributions of group average Functional connectivity values. Middle: distributions of subject specific functional connectivity values. Right: Cross subject consistencies of Functional connectivity, which is calculated as Pearson correlation coefficient between vectorised functional connectivity matrices across subjects. Subject-specific functional connectivity becomes sparser and more consistent acorss subjects in mPFMs, leading to less sparse group-average functional connectivity.

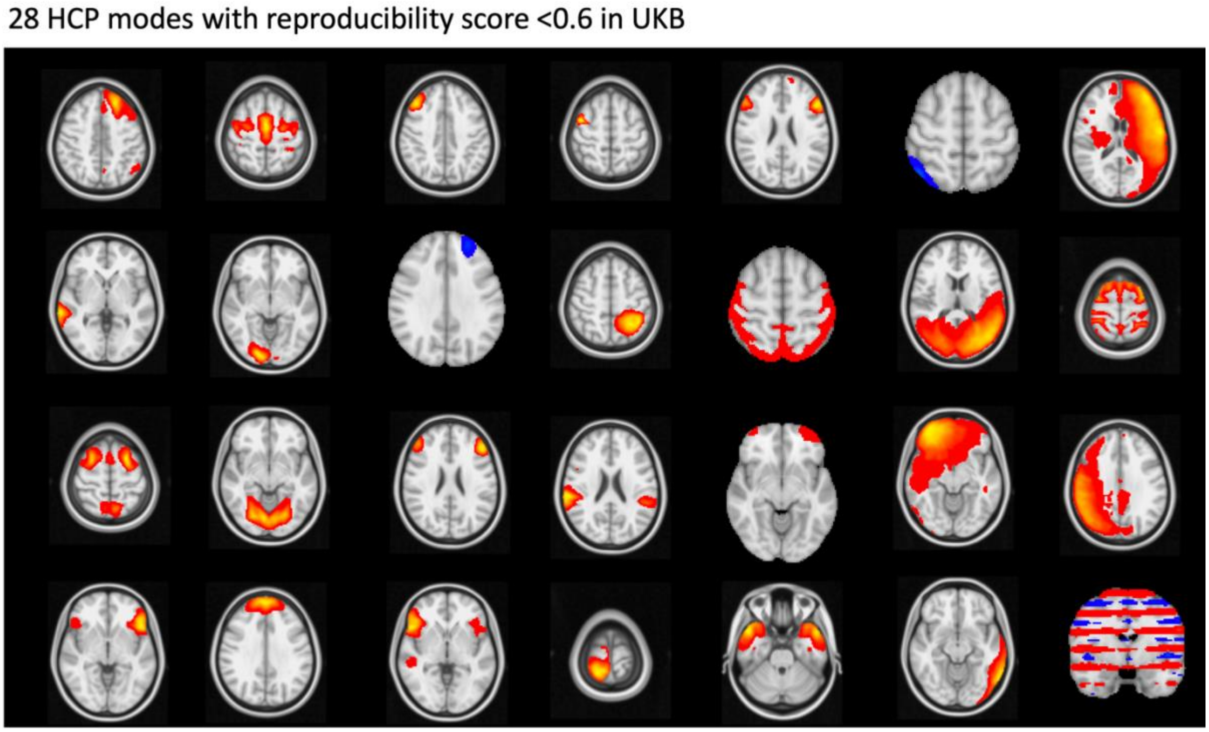


**Figure S 8 Supplement to Figure 5:** 28 Volumetric HCP modes with reproducibility scores of <0.6 in UKB. Several of these modes were artefactual.

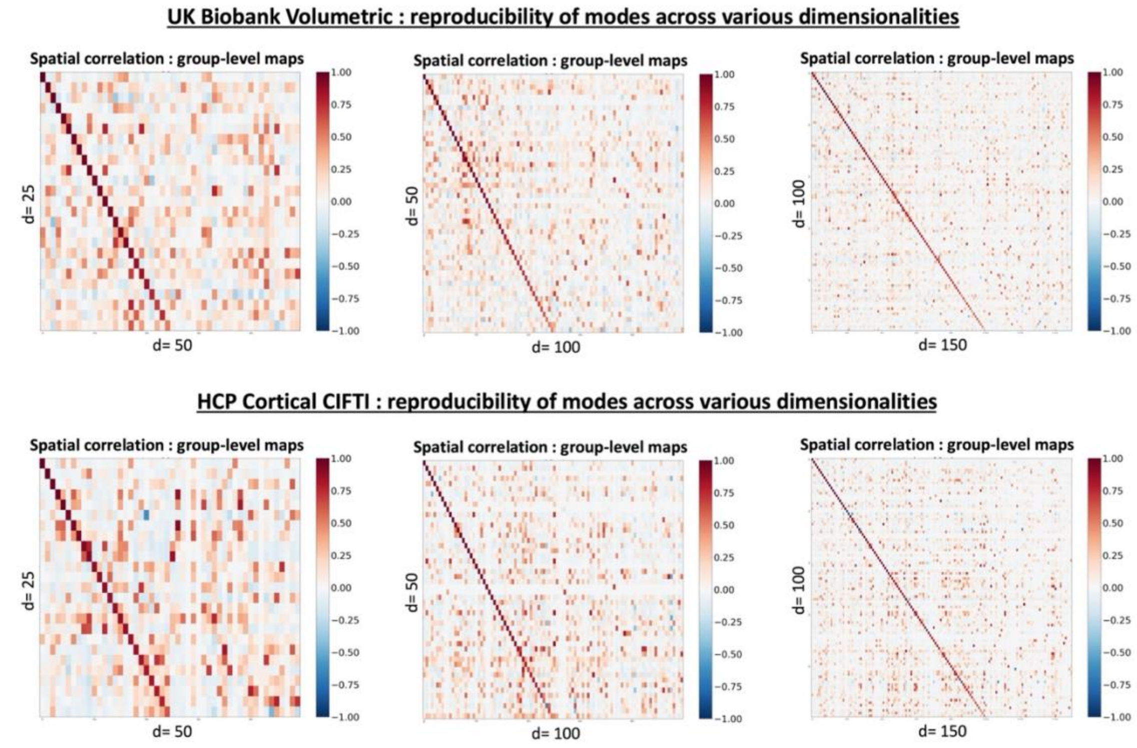


**Figure S 9 Supplement to section 3.2.2.1. Reproducibility of mPFMs across multiple dimensionalities in two independent datasets, UK Biobank and HCP**: starting from low-dimensional d=25 decomposition, we compared 25 vs 50, 50 vs 100 and 100 vs 150. The modes are sorted and paired based on spatial correlations between the group-level spatial maps. Strong diagonal elements show that, as we increase the dimensionality, the existing modes at lower dimensions are largely maintained, and new modes are added to the ensemble. This indicated an eventual addition of more and more secondary PFMs. Mean±STD of the diagonal values: for the top panel, UK Biobank volumetric fMRI: **25 vs 50**: 0.90 ± 0.11, **50 vs 100**: 0.84 ± 0.14, **100 vs 150**: 0.82 ± 0.14. For the bottom panel, HCP Cortical CIFTI fMRI: **25 vs 50**: 0.88 ± 0.08, **50 vs 100**: 0.90 ± 0.11, **100 vs 150**: 0.88 ± 0.11.

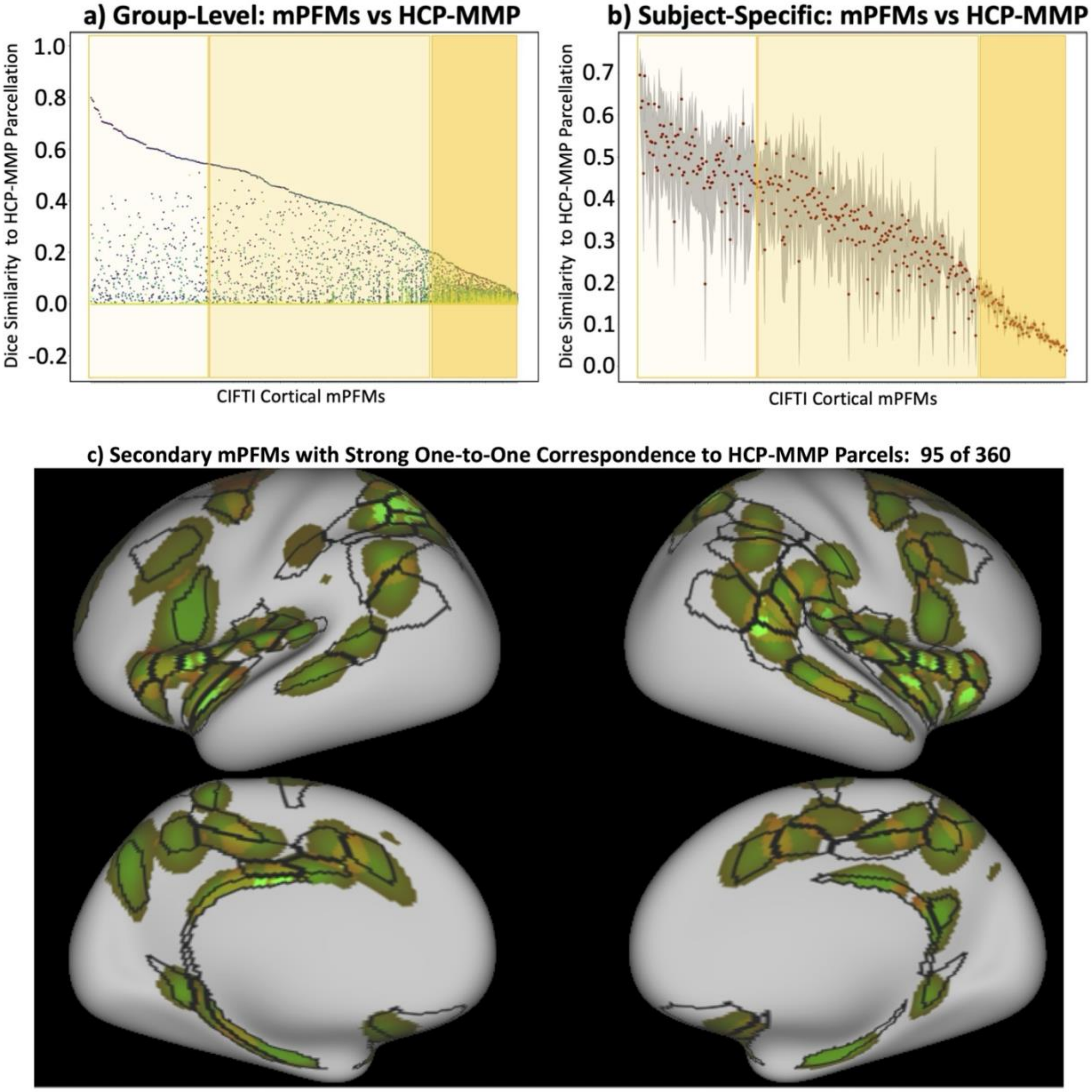


**Figure S 10 Supplement to section 3.2.3:** a) group-level HCP-MMP parcellation with 360 parcels compared to a 360-mPFM decomposition of HCP. 95 modes were parcel-like (lightest shade of yellow) and showed a clear one-to-one match to HCP-MMP parcels. The remaining large-scale and mixed-scale mPFMs are marked with dark and medium shades of yellow, respectively. 73 mPFMs showed less than 0.2 dice similarity to any HCP-MMP parcels (highlighted with the darkest shade of yellow); these included conventional low-dimensional modes and the less well-known variants of large-scale modes, and typically occupied multiple distant sub-regions. b) subject-level HCP MMP (with the same ordering as Fig5a). Median showed in red, and grey error margins show 25 to 75 percentile across subjects. Good pairing for 95 parcel-like modes were observed here as well. c) Top 95 matched pairs of modes between mPFM and HCP-MMP. Black contours denote group-level HCP-MMP parcels and green-brown patches denote thresholded group-level PFMs.

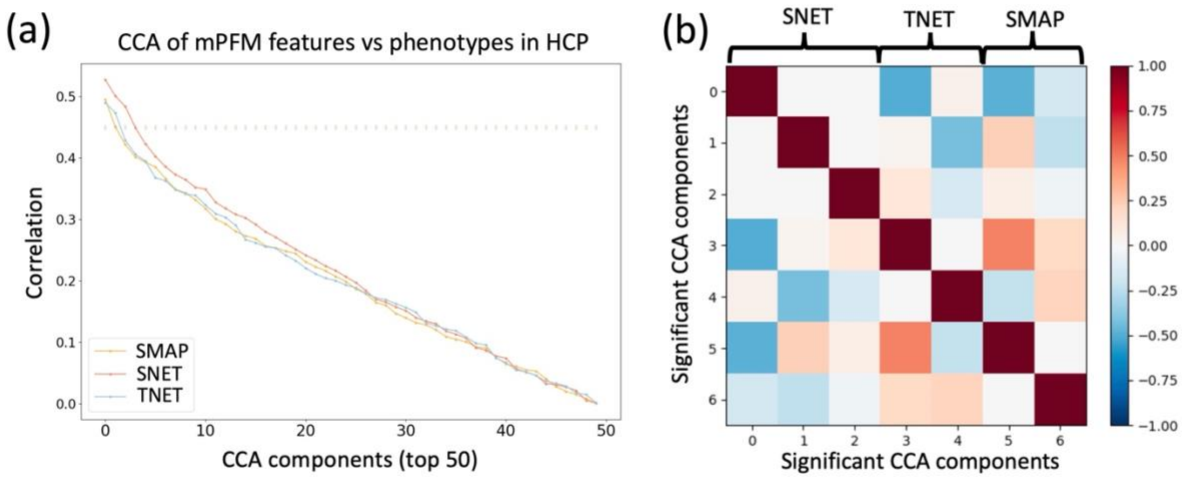


**Figure S 11 Supplement to Figure 6a: shared population covariation between mPFMs and behaviour in HCP:** a) Canonical Correlation Analysis (CCA) was used to compute modes of population co-variation between behavioural traits and spatial maps (SMAPs)/Spatial correlations (SNETs)/temporal correlations (TNETs) of mPFMs. Statistical significance of CCA components was determined using multi-level block permutations that takes family structure of HCP data into account (Winkler et al., 2015). Correlation values for the top CCA components of SMAP, SNET and TNET were found to be 0.49, 0.52 and 0.49, respectively. Using the recently-introduced GEMMR tool (Helmer et al., 2024), we verified that given these correlation values, our utilised sample size (1001) is sufficient to yield at least 85% power and at most 10% error for the association strength, weight, scores and loadings; b) 2, 3 and 2 significant CCA components were identified for SMAPs, SNETs and TNETs, respectively, and their correlations are shown. we found maximum correlations between transformed pairs SNET-SMAP, SNET-TNET, and SMAP-TNET to be -0.498, -0.484 and 0.483, respectively, indicating that the phenotype-transformed SMAPs, SNETs, and TNETs capture distinct aspects of subject variability.

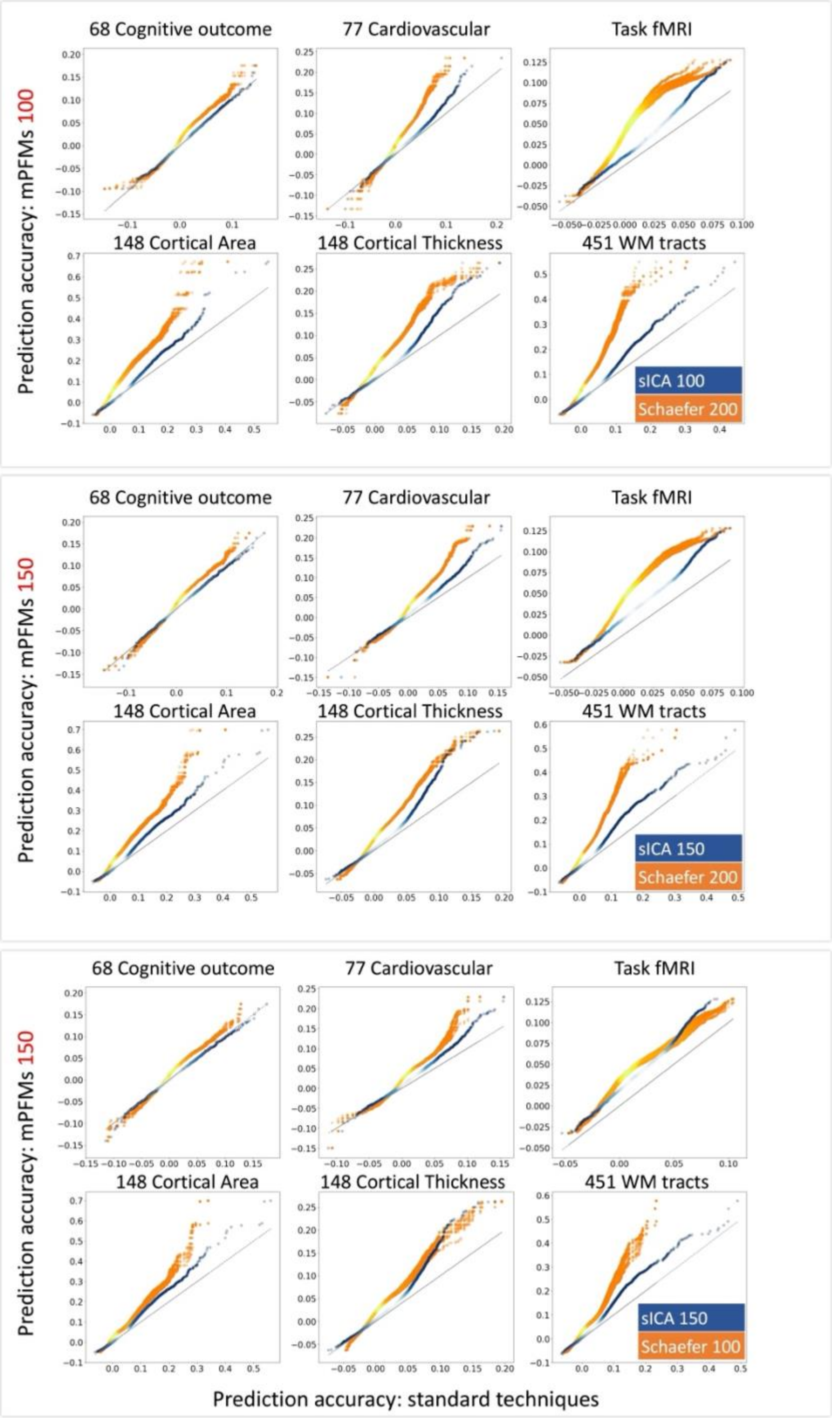


**Figure S 12 Supplement to Figure 6b: replicating trait prediction results of mPFMs vs standard techniques using different choices of dimensionality.** Top panel: comparing mPFM 100 with spatial ICA (sICA, blue) 100 and Schaefer (orange) 200; middle panel: comparing mPFM 150 with sICA 150 and Schaefer 200; bottom panel: comparing mPFM 150 with sICA 150 and Schaefer 100. In all three scenarios, similar to the original result where d=100 was used for all three methods, mPFMs significantly outpe rformed the standard techniques in trait predictions. This was especially evident in comparison to Schaefer parcellation. Note that when the number of modes is mismatched between mPFMs/sICA and Schaefer atlas, we have randomly selected a subset of the modes/parcels (e.g., 100 out of 150 in the bottom panel), and repeated that sub-selection 10 times for plotting (hence 10 orange graphs vs 1 blue, given that sICA and mPFM dimensionalities are always matched).

Table S 1 A summary of terminology used throughout the paper.

| Terminology | Description |
|---|---|
| **Functional Modes** | Modes of functional brain connectivity, where one or multiple brain regions work in synchrony such that they can be described with a single timecourse. |
| **PFMs** | Probabilistic Functional Modes: functional modes estimated using sPROFUMO software, which estimates the modes simultaneously for big populations and individuals, thus yielding *personalised* functional modes. |
| **RSNs** | Resting State Networks: functional modes estimated from resting state fMRI (rfMRI). Resting state refers to brain activity from task-free recordings, where participants are instructed to not think about anything specific while lying in the scanner. |
| **Large-scale or Distributed RSNs/PFMs** | 25 modes from low-dimensional (lowD) decomposition of rfMRI data using PFMs, unless specified otherwise (e.g., in Figure 2). |
| **Multiscale PFMs (mPFMs)** | Modes from high-dimensional (highD) decomposition of rfMRI data using PFMs. Depending on dataset/application, we have used different number of modes as highD decomposition. Specifically, for HCP data we have used 50, 100, 150 or 360 modes, for UK Biobank data we have used 150 or 100 modes. Justifications are included the corresponding Methods subsections. |
| **Primary mPFMs** | 25 distributed modes from high-dimensional PFM decomposition of rfMRI that best-match the large-scale RSNs. |
| **Secondary mPFMs** | New modes from high-dimensional PFM decomposition that emerge as we increase the dimensionality to >=100. |
| **Spatial Maps, or SMAPs** | Spatial organisation of functional modes across the brain voxels. |
| **Functional Connectivity** | A matrix of size $N_{mode} \times N_{mode}$ including partial temporal correlations between timecourses of functional modes, also referred to as TNET. L2/Tikhonov regularisation is used in estimation of partial correlations. |
| **Spatial Connectivity** | A matrix of size $N_{mode} \times N_{mode}$ including spatial correlation coefficients between SMAPs of functional modes, also referred to as SNET. This is an indicator of *spatial overlap* between the modes. |

Table S 2 T-value and p-values related to subcomponent analysis: original subcomponents. Statistically significant p-values (Bonferroni-corrected threshold to account for multiple comparisons) are shown in bold font. Supplement to **Figure 3**c&d.

| Winner: Large-Scale | | Winner: Primary mPFMs | | Winner: Secondary mPFMs | | | |
|---|---|---|---|---|---|---|---|
| **t-values** | **p-values** | **t-values** | **p-values** | **t-values** | **p-values** | **t-values** | **p-values** |
| 0.46 | 6.42e-01 | 19.81 | **5.57e-74** | 22.96 | **4.70e-94** | 23.08 | **7.82e-95** |
| 40.45 | **7.04e-213** | 17.29 | **8.47e-59** | 14.19 | **8.30e-42** | 16.34 | **2.07e-53** |
| 1.86 | 6.35e-02 | 22.53 | **2.74e-91** | 17.81 | **7.82e-62** | 29.73 | **8.81e-140** |
| 7.60 | **6.64e-14** | 14.21 | **7.29e-42** | 4.64 | **4.01e-06** | 17.66 | **5.87e-61** |
| 36.36 | **3.06e-185** | 10.26 | **1.54e-23** | 28.59 | **6.15e-132** | -0.61 | 5.43e-01 |
| 37.73 | **1.35e-194** | 5.33 | **1.21e-07** | 31.04 | **9.76e-149** | 24.86 | **1.12e-106** |
| 15.01 | **4.24e-46** | 44.60 | **3.17e-240** | 19.73 | **1.70e-73** | 15.69 | **8.63e-50** |
| -2.64 | 8.33e-03 | 15.12 | **1.07e-46** | 10.46 | **2.31e-24** | 10.89 | **3.61e-26** |
| 7.04 | **3.49e-12** | 14.02 | **6.60e-41** | 24.87 | **1.03e-106** | -0.01 | 9.94e-01 |
| 7.98 | **4.06e-15** | 57.85 | **1.13e-321** | 10.30 | **1.00e-23** | 4.77 | **2.08e-06** |
| 35.48 | **3.05e-179** | 23.84 | **7.72e-100** | 5.45 | **6.42e-08** | 11.08 | **5.24e-27** |
| 15.05 | **2.63e-46** | 14.47 | **3.29e-43** | 12.29 | **1.95e-32** | 40.87 | **1.13e-215** |
| | | 4.88 | **1.26e-06** | 4.77 | **2.08e-06** | 32.10 | **5.01e-156** |
| | | 16.06 | **8.24e-52** | 16.35 | **1.92e-53** | 18.47 | **8.46e-66** |
| | | 7.10 | **2.36e-12** | 51.75 | **2.76e-285** | 26.74 | **2.64e-119** |
| | | 37.67 | **3.76e-194** | 9.99 | **1.76e-22** | 26.53 | **7.10e-118** |
| | | 10.92 | **2.62e-26** | 27.84 | **8.20e-127** | 16.47 | **4.19e-54** |
| | | 3.23 | **1.27e-03** | 52.06 | **3.32e-287** | 35.81 | **1.68e-181** |
| | | 15.55 | **4.96e-49** | 17.32 | **5.24e-59** | 39.36 | **1.46e-205** |
| | | 17.40 | **1.91e-59** | 44.44 | **3.60e-239** | 61.30 | **0.00e+00** |
| | | 6.98 | **5.22e-12** | 10.31 | **9.63e-24** | 29.06 | **3.88e-135** |
| | | 33.82 | **7.52e-168** | 31.74 | **1.39e-153** | 17.77 | **1.28e-61** |
| | | 37.02 | **9.35e-190** | 24.91 | **5.39e-107** | 29.35 | **3.66e-137** |
| | | 26.00 | **2.47e-114** | 15.64 | **1.69e-49** | 11.63 | **1.99e-29** |
| | | 27.83 | **9.80e-127** | 24.38 | **1.78e-103** | 13.61 | **7.77e-39** |
| | | 16.07 | **6.90e-52** | 21.47 | **2.08e-84** | 34.87 | **4.61e-175** |
| | | 19.13 | **9.34e-70** | 22.20 | **3.81e-89** | 9.84 | **7.32e-22** |
| | | 38.03 | **1.39e-196** | 38.65 | **8.15e-201** | 54.03 | **3.83e-299** |
| | | | | 61.69 | **0.00e+00** | 23.29 | **2.93e-96** |
| | | | | 27.37 | **1.24e-123** | 25.14 | **1.54e-108** |
| | | | | 27.32 | **2.92e-123** | 14.85 | **2.95e-45** |
| | | | | 49.52 | **1.59e-271** | 74.34 | **0.00e+00** |
| | | | | 88.06 | **0.00e+00** | 6.00 | **2.82e-09** |
| | | | | 44.50 | **1.58e-239** | 14.35 | **1.34e-42** |
| | | | | 3.59 | **3.42e-04** | 19.80 | **6.30e-74** |
| | | | | 11.52 | **6.05e-29** | 24.71 | **1.17e-105** |
| | | | | 9.78 | **1.22e-21** | 19.73 | **1.75e-73** |
| | | | | 22.43 | **1.26e-90** | 13.38 | **1.12e-37** |
| | | | | 11.77 | **4.55e-30** | 13.04 | **5.38e-36** |
| | | | | 40.02 | **5.40e-210** | 62.63 | **0.00e+00** |
| | | | | 34.89 | **3.22e-175** | 16.04 | **1.02e-51** |
| | | | | 14.68 | **2.54e-44** | 37.97 | **3.27e-196** |
| | | | | | | 49.84 | **1.71e-273** |

Table S 3 T-value and p-values related to subcomponent analysis: temporally-exclusive subcomponents. Statistically significant p-values (Bonferroni-corrected threshold to account for multiple comparisons) are shown in bold font. Supplement to **Figure 3**e&f.

| Winner: Large-Scale | | | | Winner: Primary mPFMs | | Winner: Secondary mPFMs | |
|---|---|---|---|---|---|---|---|
| **t-values** | **p-values** | **t-values** | **p-values** | **t-values** | **p-values** | **t-values** | **p-values** |
| 30.98 | **2.52e-148** | 46.74 | **6.02e-254** | 1.52 | 1.28e-01 | 6.41 | **2.20e-10** |
| 28.70 | **1.00e-132** | 37.99 | **2.40e-196** | 3.12 | **1.88e-03** | 15.88 | **8.34e-51** |
| 15.48 | **1.20e-48** | 4.87 | **1.31e-06** | 4.21 | **2.78e-05** | 1.55 | 1.22e-01 |
| 4.11 | **4.30e-05** | 34.01 | **3.38e-169** | 1.13 | 2.59e-01 | 6.07 | **1.82e-09** |
| 41.43 | **2.13e-219** | 20.80 | **3.71e-80** | | | 11.45 | **1.27e-28** |
| 1.62 | 1.06e-01 | 35.79 | **2.27e-181** | | | 11.26 | **8.68e-28** |
| 15.50 | **9.33e-49** | 10.34 | **7.19e-24** | | | 1.69 | 9.20e-02 |
| 10.65 | **3.63e-25** | 10.02 | **1.32e-22** | | | | |
| 67.89 | **0.00e+00** | 1.27 | 2.04e-01 | | | | |
| 16.01 | **1.50e-51** | 62.64 | **0.00e+00** | | | | |
| 26.71 | **4.26e-119** | 37.78 | **6.91e-195** | | | | |
| 7.64 | **5.18e-14** | 37.84 | **2.51e-195** | | | | |
| 42.21 | **1.46e-224** | 25.98 | **3.70e-114** | | | | |
| 15.03 | **3.57e-46** | 34.11 | **7.63e-170** | | | | |
| 24.92 | **4.92e-107** | 42.65 | **1.83e-227** | | | | |
| 6.33 | **3.61e-10** | 10.44 | **2.72e-24** | | | | |
| 22.85 | **2.21e-93** | 22.93 | **6.88e-94** | | | | |
| 60.43 | **0.00e+00** | 58.42 | **0.00e+00** | | | | |
| 25.70 | **2.91e-112** | 25.22 | **4.90e-109** | | | | |
| 5.16 | **3.02e-07** | 26.83 | **6.54e-120** | | | | |
| 27.78 | **2.27e-126** | 22.02 | **5.46e-88** | | | | |
| 8.02 | **3.00e-15** | 8.93 | **1.96e-18** | | | | |
| 50.27 | **3.33e-276** | 54.66 | **6.28e-303** | | | | |
| 33.88 | **2.63e-168** | 3.63 | **2.99e-04** | | | | |
| 27.60 | **3.55e-125** | 3.79 | **1.58e-04** | | | | |
| 17.19 | **3.05e-58** | 2.38 | 1.75e-02 | | | | |
| 13.68 | **3.60e-39** | 72.20 | **0.00e+00** | | | | |
| 71.00 | **0.00e+00** | 44.52 | **1.04e-239** | | | | |
| 43.53 | **3.04e-233** | 21.80 | **1.47e-86** | | | | |
| 34.39 | **9.00e-172** | 23.88 | **3.76e-100** | | | | |
| 30.32 | **8.00e-144** | 4.15 | **3.59e-05** | | | | |
| 35.19 | **2.98e-177** | 58.48 | **0.00e+00** | | | | |
| 38.02 | **1.57e-196** | 24.23 | **1.77e-102** | | | | |
| 33.46 | **2.09e-165** | 8.54 | **4.82e-17** | | | | |
| 28.81 | **1.82e-133** | 105.56 | **0.00e+00** | | | | |
| 23.01 | **2.10e-94** | 29.69 | **1.62e-139** | | | | |
| 21.48 | **1.81e-84** | 23.61 | **2.41e-98** | | | | |
| 49.70 | **1.10e-272** | 34.32 | **2.69e-171** | | | | |
| 30.70 | **2.12e-146** | 27.49 | **2.07e-124** | | | | |
| 20.28 | **6.98e-77** | 69.98 | **0.00e+00** | | | | |
| 16.30 | **3.74e-53** | 10.83 | **6.31e-26** | | | | |
| 24.46 | **5.31e-104** | 9.19 | **2.15e-19** | | | | |
| 63.10 | **0.00e+00** | 14.30 | **2.33e-42** | | | | |
| 3.10 | 1.97e-03 | 4.85 | **1.40e-06** | | | | |
| 12.88 | **3.22e-35** | 38.13 | **2.64e-197** | | | | |
| 18.38 | **3.02e-65** | 23.52 | **1.00e-97** | | | | |
| 12.50 | **2.12e-33** | 11.53 | **5.92e-29** | | | | |
| 78.92 | **0.00e+00** | 74.21 | **0.00e+00** | | | | |
| 35.71 | **8.46e-181** | 18.10 | **1.39e-63** | | | | |
| 19.95 | **7.70e-75** | 42.58 | **5.12e-227** | | | | |
| 30.69 | **2.35e-146** | 31.93 | **6.42e-155** | | | | |
| 25.75 | **1.29e-112** | 38.12 | **3.17e-197** | | | | |
| 19.17 | **4.85e-70** | 91.42 | **0.00e+00** | | | | |
| 38.45 | **1.86e-199** | 29.99 | **1.49e-141** | | | | |
| 21.55 | **5.77e-85** | 30.16 | **9.74e-143** | | | | |
| 23.77 | **2.17e-99** | 27.38 | **1.14e-123** | | | | |
| 2.54 | 1.12e-02 | 22.64 | **5.45e-92** | | | | |

Table S 4 T-value and p-values related to subcomponent analysis: spatially-exclusive subcomponents. Statistically significant p-values (Bonferroni-corrected threshold to account for multiple comparisons) are shown in bold font. Supplement to **Figure 3**g&h.

| Winner: Large-Scale | | Winner: Primary mPFMs | | Winner: Secondary mPFMs | | | |
|---|---|---|---|---|---|---|---|
| t-values | p-values | t-values | p-values | t-values | p-values | t-values | p-values |
| 5.69 | **1.66e-08** | 6.72 | **2.99e-11** | 9.19 | **2.26e-19** | 34.20 | **1.78e-170** |
| 5.38 | **9.08e-08** | 5.23 | **2.08e-07** | 7.34 | **4.28e-13** | 26.13 | **3.38e-115** |
| 6.52 | **1.13e-10** | 15.49 | **1.18e-48** | 26.05 | **1.25e-114** | 2.76 | 5.98e-03 |
| 13.65 | **5.09e-39** | 15.99 | **2.08e-51** | 34.10 | **8.66e-170** | 40.76 | **5.93e-215** |
| 0.13 | 8.96e-01 | 21.76 | **2.96e-86** | 27.08 | **1.23e-121** | 18.72 | **2.89e-67** |
| 26.21 | **9.44e-116** | 14.93 | **1.17e-45** | 43.91 | **1.10e-235** | 23.28 | **3.76e-96** |
| 61.72 | **0.00e+00** | 10.59 | **6.59e-25** | 5.19 | **2.51e-07** | 13.26 | **4.26e-37** |
| 22.23 | **2.76e-89** | 18.96 | **1.03e-68** | 4.75 | **2.30e-06** | 53.60 | **1.43e-296** |
| 16.48 | **3.43e-54** | 3.97 | **7.59e-05** | 49.56 | **9.49e-272** | 57.32 | **1.46e-318** |
| 0.59 | 5.55e-01 | 38.36 | **7.99e-199** | 12.44 | **3.84e-33** | 31.82 | **4.05e-154** |
| 5.10 | **4.14e-07** | 8.41 | **1.43e-16** | 31.73 | **1.77e-153** | 9.44 | **2.56e-20** |
| 3.46 | **5.65e-04** | 19.28 | **1.02e-70** | 3.03 | 2.49e-03 | 14.04 | **5.04e-41** |
| 3.33 | **9.01e-04** | 5.47 | **5.58e-08** | 55.94 | **1.62e-310** | 34.32 | **2.67e-171** |
| 1.65 | 9.96e-02 | 52.83 | **6.65e-292** | 14.90 | **1.68e-45** | 26.62 | **1.73e-118** |
| 8.39 | **1.63e-16** | 22.88 | **1.58e-93** | 4.61 | **4.54e-06** | 49.63 | **3.14e-272** |
| 11.01 | **1.13e-26** | 12.22 | **4.24e-32** | 22.67 | **3.51e-92** | 13.06 | **4.35e-36** |
| 19.96 | **7.23e-75** | 3.86 | **1.19e-04** | 20.11 | **7.89e-76** | 26.66 | **8.40e-119** |
| 21.98 | **1.08e-87** | 15.40 | **3.47e-48** | 1.22 | 2.21e-01 | 44.86 | **7.14e-242** |
| 11.82 | **2.91e-30** | 4.54 | **6.24e-06** | 45.25 | **1.96e-244** | 26.79 | **1.24e-119** |
| 15.23 | **2.86e-47** | 27.83 | **9.10e-127** | 15.46 | **1.67e-48** | 21.68 | **8.53e-86** |
| 15.47 | **1.43e-48** | 27.79 | **1.69e-126** | 15.53 | **6.65e-49** | 5.08 | **4.61e-07** |
| | | 17.66 | **6.01e-61** | 0.24 | 8.10e-01 | 31.60 | **1.20e-152** |
| | | 27.53 | **1.12e-124** | 22.53 | **2.79e-91** | 30.37 | **3.89e-144** |
| | | 15.86 | **1.07e-50** | 45.45 | **1.05e-245** | 52.73 | **2.57e-291** |
| | | 21.31 | **2.11e-83** | 3.94 | **8.72e-05** | 14.26 | **3.83e-42** |
| | | 18.86 | **3.69e-68** | 36.17 | **5.61e-184** | 55.50 | **7.10e-308** |
| | | 23.85 | **6.66e-100** | 18.62 | **1.05e-66** | 13.58 | **1.05e-38** |
| | | | | 12.19 | **5.60e-32** | 12.37 | **8.18e-33** |
| | | | | 38.93 | **1.22e-202** | 11.22 | **1.35e-27** |
| | | | | 45.34 | **5.24e-245** | 25.39 | **3.53e-110** |
| | | | | 6.91 | **8.36e-12** | 24.99 | **1.66e-107** |
| | | | | 9.67 | **3.17e-21** | 18.15 | **7.23e-64** |
| | | | | 6.76 | **2.32e-11** | 25.25 | **3.02e-109** |
| | | | | 18.88 | **2.96e-68** | 75.50 | **0.00e+00** |
| | | | | 4.75 | **2.31e-06** | 8.23 | **5.80e-16** |
| | | | | 18.66 | **6.15e-67** | 38.35 | **9.55e-199** |
| | | | | 20.49 | **3.29e-78** | 14.31 | **2.02e-42** |
| | | | | 10.18 | **3.28e-23** | 27.85 | **6.72e-127** |
| | | | | | | 33.14 | **3.38e-163** |

Table S 5 T-value and Bonferroni-corrected (36 comparisons) p-values to compare phenotype-prediction performance of Spatial and Temporal/Functional connectivity features related to: a) LowD large-scale RSNs vs highD mPFMs and b) within-scale vs cross-scale mPFMs. Pri: Primary, Sec: Secondary, Statistically significant values are shown in bold font. Supplement to **Figure 4**.

| | **T-Value (corrected P-Value)** | | | | | |
|---|---|---|---|---|---|---|
| | **Spatial connectivity** | | | **Temporal connectivity** | | |
| | **lowD > mPFMs** | **Pri2Pri > Pri2Sec** | **Sec2Sec > Pri2Sec** | **lowD > mPFMs** | **Pri2Pri > Pri2Sec** | **Sec2Sec > Pri2Sec** |
| **Cognitive** | -1.464 (0.148) | -1.243 (0.219) | **-3.526 (7.92E-04)** | -0.6182 (0.539) | -1.5678 (0.122) | -0.487 (0.628) |
| **GMa** | **-21.657 (1.37E-47)** | **-21.017 (3.89E-46)** | **-14.978 (2.21E-31)** | **-4.6115 (8.88E-06)** | **-2.6633 (8.64E-03)** | **3.3587 (1.01E-03)** |
| **GMt** | **-9.226 (2.81E-16)** | **-12.042 (1.14E-23)** | **-4.916 (2.34E-06)** | -1.0236 (0.31) | -0.1672 (0.867) | **2.0203 (0.045)** |
| **WM** | **-26.469 (6.31E-94)** | **-25.468 (2.19E-89)** | -0.583 (0.56) | 1.0486 (0.295) | **-9.8976 (5.09E-21)** | **7.1892 (2.75E-12)** |
| **Task-Contrast** | **-15.190 (4.15E-35)** | **-7.165 (1.48E-11)** | **-56.461 (1.94E-124)** | **-7.0706 (2.56E-11)** | -0.6513 (0.516) | **35.8817 (7.73E-89)** |
| **Cardiovascular** | **-7.272 (2.68E-10)** | **-4.296 (5.09E-05)** | 0.663 (0.51) | 0.2176 (0.828) | **-5.4334 (6.59E-07)** | -0.5626 (0.575) |

Table S 6 T-values and Bonferroni-corrected (6 comparisons) p-values to compare phenotype-prediction performance of mPFMs vs spatial ICA (sICA) and Schaefer Parcellation of the same dimensionality (100 modes). Statistically significant values have been shown in bold font. Supplement to **Figure 6**.

| | T-value | | | Corrected P-Value | | |
|---|---|---|---|---|---|---|
| | **mPFM > sICA** | **mPFM > Schaefer** | **sICA > Schaefer** | **mPFM vs sICA** | **mPFM vs Schaefer** | **sICA vs Schaefer** |
| **Cognitive** | 1.261 | **7.385** | **7.252** | 0.212 | **3.61E-10** | **6.21E-10** |
| **GMa** | **15.137** | **26.991** | **23.107** | **8.57E-32** | **7.90E-59** | **8.59E-51** |
| **GMt** | **13.964** | **33.923** | **31.499** | **9.64E-29** | **2.09E-71** | **3.06E-67** |
| **WM** | **12.675** | **51.843** | **57.347** | **1.07E-31** | **6.96E-192** | **5.19E-209** |
| **Task-Contrast** | **14.572** | **14.496** | **5.086** | **1.93E-19** | **2.38E-19** | **5.75E-06** |
| **Cardiovascular** | **7.974** | **17.409** | **17.33** | **1.24E-11** | **3.07E-28** | **4.04E-28** |

Table S 7 Names of UK Biobank Imaging Derived Phenotypes (IDPs) related to GM area: 148. Supplement to **Figure 6**.

| IDP # | IDP Name | IDP # | IDP Name |
|---|---|---|---|
| **1** | aparc-a2009s_lh_area_G+S-frontomargin | **75** | aparc-a2009s_rh_area_G+S-frontomargin |
| **2** | aparc-a2009s_lh_area_G+S-occipital-inf | **76** | aparc-a2009s_rh_area_G+S-occipital-inf |
| **3** | aparc-a2009s_lh_area_G+S-paracentral | **77** | aparc-a2009s_rh_area_G+S-paracentral |
| **4** | aparc-a2009s_lh_area_G+S-subcentral | **78** | aparc-a2009s_rh_area_G+S-subcentral |
| **5** | aparc-a2009s_lh_area_G+S-transv-frontopol | **79** | aparc-a2009s_rh_area_G+S-transv-frontopol |
| **6** | aparc-a2009s_lh_area_G+S-cingul-Ant | **80** | aparc-a2009s_rh_area_G+S-cingul-Ant |
| **7** | aparc-a2009s_lh_area_G+S-cingul-Mid-Ant | **81** | aparc-a2009s_rh_area_G+S-cingul-Mid-Ant |
| **8** | aparc-a2009s_lh_area_G+S-cingul-Mid-Post | **82** | aparc-a2009s_rh_area_G+S-cingul-Mid-Post |
| **9** | aparc-a2009s_lh_area_G-cingul-Post-dorsal | **83** | aparc-a2009s_rh_area_G-cingul-Post-dorsal |
| **10** | aparc-a2009s_lh_area_G-cingul-Post-ventral | **84** | aparc-a2009s_rh_area_G-cingul-Post-ventral |
| **11** | aparc-a2009s_lh_area_G-cuneus | **85** | aparc-a2009s_rh_area_G-cuneus |
| **12** | aparc-a2009s_lh_area_G-front-inf-Opercular | **86** | aparc-a2009s_rh_area_G-front-inf-Opercular |
| **13** | aparc-a2009s_lh_area_G-front-inf-Orbital | **87** | aparc-a2009s_rh_area_G-front-inf-Orbital |
| **14** | aparc-a2009s_lh_area_G-front-inf-Triangul | **88** | aparc-a2009s_rh_area_G-front-inf-Triangul |
| **15** | aparc-a2009s_lh_area_G-front-middle | **89** | aparc-a2009s_rh_area_G-front-middle |
| **16** | aparc-a2009s_lh_area_G-front-sup | **90** | aparc-a2009s_rh_area_G-front-sup |
| **17** | aparc-a2009s_lh_area_G-Ins-lg+S-cent-ins | **91** | aparc-a2009s_rh_area_G-Ins-lg+S-cent-ins |
| **18** | aparc-a2009s_lh_area_G-insular-short | **92** | aparc-a2009s_rh_area_G-insular-short |
| **19** | aparc-a2009s_lh_area_G-occipital-middle | **93** | aparc-a2009s_rh_area_G-occipital-middle |
| **20** | aparc-a2009s_lh_area_G-occipital-sup | **94** | aparc-a2009s_rh_area_G-occipital-sup |
| **21** | aparc-a2009s_lh_area_G-oc-temp-lat-fusifor | **95** | aparc-a2009s_rh_area_G-oc-temp-lat-fusifor |
| **22** | aparc-a2009s_lh_area_G-oc-temp-med-Lingual | **96** | aparc-a2009s_rh_area_G-oc-temp-med-Lingual |
| **23** | aparc-a2009s_lh_area_G-oc-temp-med-Parahip | **97** | aparc-a2009s_rh_area_G-oc-temp-med-Parahip |
| **24** | aparc-a2009s_lh_area_G-orbital | **98** | aparc-a2009s_rh_area_G-orbital |
| **25** | aparc-a2009s_lh_area_G-pariet-inf-Angular | **99** | aparc-a2009s_rh_area_G-pariet-inf-Angular |
| **26** | aparc-a2009s_lh_area_G-pariet-inf-Supramar | **100** | aparc-a2009s_rh_area_G-pariet-inf-Supramar |
| **27** | aparc-a2009s_lh_area_G-parietal-sup | **101** | aparc-a2009s_rh_area_G-parietal-sup |
| **28** | aparc-a2009s_lh_area_G-postcentral | **102** | aparc-a2009s_rh_area_G-postcentral |
| **29** | aparc-a2009s_lh_area_G-precentral | **103** | aparc-a2009s_rh_area_G-precentral |
| **30** | aparc-a2009s_lh_area_G-precuneus | **104** | aparc-a2009s_rh_area_G-precuneus |
| **31** | aparc-a2009s_lh_area_G-rectus | **105** | aparc-a2009s_rh_area_G-rectus |
| **32** | aparc-a2009s_lh_area_G-subcallosal | **106** | aparc-a2009s_rh_area_G-subcallosal |
| **33** | aparc-a2009s_lh_area_G-temp-sup-G-T-transv | **107** | aparc-a2009s_rh_area_G-temp-sup-G-T-transv |
| **34** | aparc-a2009s_lh_area_G-temp-sup-Lateral | **108** | aparc-a2009s_rh_area_G-temp-sup-Lateral |
| **35** | aparc-a2009s_lh_area_G-temp-sup-Plan-polar | **109** | aparc-a2009s_rh_area_G-temp-sup-Plan-polar |
| **36** | aparc-a2009s_lh_area_G-temp-sup-Plan-tempo | **110** | aparc-a2009s_rh_area_G-temp-sup-Plan-tempo |
| **37** | aparc-a2009s_lh_area_G-temporal-inf | **111** | aparc-a2009s_rh_area_G-temporal-inf |
| **38** | aparc-a2009s_lh_area_G-temporal-middle | **112** | aparc-a2009s_rh_area_G-temporal-middle |
| **39** | aparc-a2009s_lh_area_Lat-Fis-ant-Horizont | **113** | aparc-a2009s_rh_area_Lat-Fis-ant-Horizont |
| **40** | aparc-a2009s_lh_area_Lat-Fis-ant-Vertical | **114** | aparc-a2009s_rh_area_Lat-Fis-ant-Vertical |
| **41** | aparc-a2009s_lh_area_Lat-Fis-post | **115** | aparc-a2009s_rh_area_Lat-Fis-post |
| **42** | aparc-a2009s_lh_area_Pole-occipital | **116** | aparc-a2009s_rh_area_Pole-occipital |
| **43** | aparc-a2009s_lh_area_Pole-temporal | **117** | aparc-a2009s_rh_area_Pole-temporal |
| **44** | aparc-a2009s_lh_area_S-calcarine | **118** | aparc-a2009s_rh_area_S-calcarine |
| **45** | aparc-a2009s_lh_area_S-central | **119** | aparc-a2009s_rh_area_S-central |
| **46** | aparc-a2009s_lh_area_S-cingul-Marginalis | **120** | aparc-a2009s_rh_area_S-cingul-Marginalis |
| **47** | aparc-a2009s_lh_area_S-circular-insula-ant | **121** | aparc-a2009s_rh_area_S-circular-insula-ant |
| **48** | aparc-a2009s_lh_area_S-circular-insula-inf | **122** | aparc-a2009s_rh_area_S-circular-insula-inf |
| **49** | aparc-a2009s_lh_area_S-circular-insula-sup | **123** | aparc-a2009s_rh_area_S-circular-insula-sup |
| **50** | aparc-a2009s_lh_area_S-collat-transv-ant | **124** | aparc-a2009s_rh_area_S-collat-transv-ant |
| **51** | aparc-a2009s_lh_area_S-collat-transv-post | **125** | aparc-a2009s_rh_area_S-collat-transv-post |
| **52** | aparc-a2009s_lh_area_S-front-inf | **126** | aparc-a2009s_rh_area_S-front-inf |
| **53** | aparc-a2009s_lh_area_S-front-middle | **127** | aparc-a2009s_rh_area_S-front-middle |
| **54** | aparc-a2009s_lh_area_S-front-sup | **128** | aparc-a2009s_rh_area_S-front-sup |
| **55** | aparc-a2009s_lh_area_S-interm-prim-Jensen | **129** | aparc-a2009s_rh_area_S-interm-prim-Jensen |
| **56** | aparc-a2009s_lh_area_S-intrapariet+P-trans | **130** | aparc-a2009s_rh_area_S-intrapariet+P-trans |
| **57** | aparc-a2009s_lh_area_S-oc-middle+Lunatus | **131** | aparc-a2009s_rh_area_S-oc-middle+Lunatus |
| **58** | aparc-a2009s_lh_area_S-oc-sup+transversal | **132** | aparc-a2009s_rh_area_S-oc-sup+transversal |
| **59** | aparc-a2009s_lh_area_S-occipital-ant | **133** | aparc-a2009s_rh_area_S-occipital-ant |
| **60** | aparc-a2009s_lh_area_S-oc-temp-lat | **134** | aparc-a2009s_rh_area_S-oc-temp-lat |
| **61** | aparc-a2009s_lh_area_S-oc-temp-med+Lingual | **135** | aparc-a2009s_rh_area_S-oc-temp-med+Lingual |
| **62** | aparc-a2009s_lh_area_S-orbital-lateral | **136** | aparc-a2009s_rh_area_S-orbital-lateral |
| **63** | aparc-a2009s_lh_area_S-orbital-med-olfact | **137** | aparc-a2009s_rh_area_S-orbital-med-olfact |
| **64** | aparc-a2009s_lh_area_S-orbital-H-Shaped | **138** | aparc-a2009s_rh_area_S-orbital-H-Shaped |
| **65** | aparc-a2009s_lh_area_S-parieto-occipital | **139** | aparc-a2009s_rh_area_S-parieto-occipital |
| **66** | aparc-a2009s_lh_area_S-pericallosal | **140** | aparc-a2009s_rh_area_S-pericallosal |
| **67** | aparc-a2009s_lh_area_S-postcentral | **141** | aparc-a2009s_rh_area_S-postcentral |
| **68** | aparc-a2009s_lh_area_S-precentral-inf-part | **142** | aparc-a2009s_rh_area_S-precentral-inf-part |
| **69** | aparc-a2009s_lh_area_S-precentral-sup-part | **143** | aparc-a2009s_rh_area_S-precentral-sup-part |
| **70** | aparc-a2009s_lh_area_S-suborbital | **144** | aparc-a2009s_rh_area_S-suborbital |
| **71** | aparc-a2009s_lh_area_S-subparietal | **145** | aparc-a2009s_rh_area_S-subparietal |
| **72** | aparc-a2009s_lh_area_S-temporal-inf | **146** | aparc-a2009s_rh_area_S-temporal-inf |
| **73** | aparc-a2009s_lh_area_S-temporal-sup | **147** | aparc-a2009s_rh_area_S-temporal-sup |
| **74** | aparc-a2009s_lh_area_S-temporal-transverse | **148** | aparc-a2009s_rh_area_S-temporal-transverse |

Table S 8 Names of UK Biobank Imaging Derived Phenotypes (IDPs) related to Grey Matter thickness: 148. Supplement to **Figure 6**.

| IDP # | IDP Name | IDP # | IDP Name |
|---|---|---|---|
| 1 | aparc-a2009s_lh_thickness_G+S-frontomargin, | 75 | aparc-a2009s_rh_thickness_G+S-frontomargin, |
| 2 | aparc-a2009s_lh_thickness_G+S-occipital-inf, | 76 | aparc-a2009s_rh_thickness_G+S-occipital-inf, |
| 3 | aparc-a2009s_lh_thickness_G+S-paracentral, | 77 | aparc-a2009s_rh_thickness_G+S-paracentral, |
| 4 | aparc-a2009s_lh_thickness_G+S-subcentral, | 78 | aparc-a2009s_rh_thickness_G+S-subcentral, |
| 5 | aparc-a2009s_lh_thickness_G+S-transv-frontopol, | 79 | aparc-a2009s_rh_thickness_G+S-transv-frontopol, |
| 6 | aparc-a2009s_lh_thickness_G+S-cingul-Ant, | 80 | aparc-a2009s_rh_thickness_G+S-cingul-Ant, |
| 7 | aparc-a2009s_lh_thickness_G+S-cingul-Mid-Ant, | 81 | aparc-a2009s_rh_thickness_G+S-cingul-Mid-Ant, |
| 8 | aparc-a2009s_lh_thickness_G+S-cingul-Mid-Post, | 82 | aparc-a2009s_rh_thickness_G+S-cingul-Mid-Post, |
| 9 | aparc-a2009s_lh_thickness_G-cingul-Post-dorsal, | 83 | aparc-a2009s_rh_thickness_G-cingul-Post-dorsal, |
| 10 | aparc-a2009s_lh_thickness_G-cingul-Post-ventral, | 84 | aparc-a2009s_rh_thickness_G-cingul-Post-ventral, |
| 11 | aparc-a2009s_lh_thickness_G-cuneus, | 85 | aparc-a2009s_rh_thickness_G-cuneus, |
| 12 | aparc-a2009s_lh_thickness_G-front-inf-Opercular, | 86 | aparc-a2009s_rh_thickness_G-front-inf-Opercular, |
| 13 | aparc-a2009s_lh_thickness_G-front-inf-Orbital, | 87 | aparc-a2009s_rh_thickness_G-front-inf-Orbital, |
| 14 | aparc-a2009s_lh_thickness_G-front-inf-Triangul, | 88 | aparc-a2009s_rh_thickness_G-front-inf-Triangul, |
| 15 | aparc-a2009s_lh_thickness_G-front-middle, | 89 | aparc-a2009s_rh_thickness_G-front-middle, |
| 16 | aparc-a2009s_lh_thickness_G-front-sup, | 90 | aparc-a2009s_rh_thickness_G-front-sup, |
| 17 | aparc-a2009s_lh_thickness_G-Ins-lg+S-cent-ins, | 91 | aparc-a2009s_rh_thickness_G-Ins-lg+S-cent-ins, |
| 18 | aparc-a2009s_lh_thickness_G-insular-short, | 92 | aparc-a2009s_rh_thickness_G-insular-short, |
| 19 | aparc-a2009s_lh_thickness_G-occipital-middle, | 93 | aparc-a2009s_rh_thickness_G-occipital-middle, |
| 20 | aparc-a2009s_lh_thickness_G-occipital-sup, | 94 | aparc-a2009s_rh_thickness_G-occipital-sup, |
| 21 | aparc-a2009s_lh_thickness_G-oc-temp-lat-fusifor, | 95 | aparc-a2009s_rh_thickness_G-oc-temp-lat-fusifor, |
| 22 | aparc-a2009s_lh_thickness_G-oc-temp-med-Lingual, | 96 | aparc-a2009s_rh_thickness_G-oc-temp-med-Lingual, |
| 23 | aparc-a2009s_lh_thickness_G-oc-temp-med-Parahip, | 97 | aparc-a2009s_rh_thickness_G-oc-temp-med-Parahip, |
| 24 | aparc-a2009s_lh_thickness_G-orbital, | 98 | aparc-a2009s_rh_thickness_G-orbital, |
| 25 | aparc-a2009s_lh_thickness_G-pariet-inf-Angular, | 99 | aparc-a2009s_rh_thickness_G-pariet-inf-Angular, |
| 26 | aparc-a2009s_lh_thickness_G-pariet-inf-Supramar, | 100 | aparc-a2009s_rh_thickness_G-pariet-inf-Supramar, |
| 27 | aparc-a2009s_lh_thickness_G-parietal-sup, | 101 | aparc-a2009s_rh_thickness_G-parietal-sup, |
| 28 | aparc-a2009s_lh_thickness_G-postcentral, | 102 | aparc-a2009s_rh_thickness_G-postcentral, |
| 29 | aparc-a2009s_lh_thickness_G-precentral, | 103 | aparc-a2009s_rh_thickness_G-precentral, |
| 30 | aparc-a2009s_lh_thickness_G-precuneus, | 104 | aparc-a2009s_rh_thickness_G-precuneus, |
| 31 | aparc-a2009s_lh_thickness_G-rectus, | 105 | aparc-a2009s_rh_thickness_G-rectus, |
| 32 | aparc-a2009s_lh_thickness_G-subcallosal, | 106 | aparc-a2009s_rh_thickness_G-subcallosal, |
| 33 | aparc-a2009s_lh_thickness_G-temp-sup-G-T-transv, | 107 | aparc-a2009s_rh_thickness_G-temp-sup-G-T-transv, |
| 34 | aparc-a2009s_lh_thickness_G-temp-sup-Lateral, | 108 | aparc-a2009s_rh_thickness_G-temp-sup-Lateral, |
| 35 | aparc-a2009s_lh_thickness_G-temp-sup-Plan-polar, | 109 | aparc-a2009s_rh_thickness_G-temp-sup-Plan-polar, |
| 36 | aparc-a2009s_lh_thickness_G-temp-sup-Plan-tempo, | 110 | aparc-a2009s_rh_thickness_G-temp-sup-Plan-tempo, |
| 37 | aparc-a2009s_lh_thickness_G-temporal-inf, | 111 | aparc-a2009s_rh_thickness_G-temporal-inf, |
| 38 | aparc-a2009s_lh_thickness_G-temporal-middle, | 112 | aparc-a2009s_rh_thickness_G-temporal-middle, |
| 39 | aparc-a2009s_lh_thickness_Lat-Fis-ant-Horizont, | 113 | aparc-a2009s_rh_thickness_Lat-Fis-ant-Horizont, |
| 40 | aparc-a2009s_lh_thickness_Lat-Fis-ant-Vertical, | 114 | aparc-a2009s_rh_thickness_Lat-Fis-ant-Vertical, |
| 41 | aparc-a2009s_lh_thickness_Lat-Fis-post, | 115 | aparc-a2009s_rh_thickness_Lat-Fis-post, |
| 42 | aparc-a2009s_lh_thickness_Pole-occipital, | 116 | aparc-a2009s_rh_thickness_Pole-occipital, |
| 43 | aparc-a2009s_lh_thickness_Pole-temporal, | 117 | aparc-a2009s_rh_thickness_Pole-temporal, |
| 44 | aparc-a2009s_lh_thickness_S-calcarine, | 118 | aparc-a2009s_rh_thickness_S-calcarine, |
| 45 | aparc-a2009s_lh_thickness_S-central, | 119 | aparc-a2009s_rh_thickness_S-central, |
| 46 | aparc-a2009s_lh_thickness_S-cingul-Marginalis, | 120 | aparc-a2009s_rh_thickness_S-cingul-Marginalis, |
| 47 | aparc-a2009s_lh_thickness_S-circular-insula-ant, | 121 | aparc-a2009s_rh_thickness_S-circular-insula-ant, |
| 48 | aparc-a2009s_lh_thickness_S-circular-insula-inf, | 122 | aparc-a2009s_rh_thickness_S-circular-insula-inf, |
| 49 | aparc-a2009s_lh_thickness_S-circular-insula-sup, | 123 | aparc-a2009s_rh_thickness_S-circular-insula-sup, |
| 50 | aparc-a2009s_lh_thickness_S-collat-transv-ant, | 124 | aparc-a2009s_rh_thickness_S-collat-transv-ant, |
| 51 | aparc-a2009s_lh_thickness_S-collat-transv-post, | 125 | aparc-a2009s_rh_thickness_S-collat-transv-post, |
| 52 | aparc-a2009s_lh_thickness_S-front-inf, | 126 | aparc-a2009s_rh_thickness_S-front-inf, |
| 53 | aparc-a2009s_lh_thickness_S-front-middle, | 127 | aparc-a2009s_rh_thickness_S-front-middle, |
| 54 | aparc-a2009s_lh_thickness_S-front-sup, | 128 | aparc-a2009s_rh_thickness_S-front-sup, |
| 55 | aparc-a2009s_lh_thickness_S-interm-prim-Jensen, | 129 | aparc-a2009s_rh_thickness_S-interm-prim-Jensen, |
| 56 | aparc-a2009s_lh_thickness_S-intrapariet+P-trans, | 130 | aparc-a2009s_rh_thickness_S-intrapariet+P-trans, |
| 57 | aparc-a2009s_lh_thickness_S-oc-middle+Lunatus, | 131 | aparc-a2009s_rh_thickness_S-oc-middle+Lunatus, |
| 58 | aparc-a2009s_lh_thickness_S-oc-sup+transversal, | 132 | aparc-a2009s_rh_thickness_S-oc-sup+transversal, |
| 59 | aparc-a2009s_lh_thickness_S-occipital-ant, | 133 | aparc-a2009s_rh_thickness_S-occipital-ant, |
| 60 | aparc-a2009s_lh_thickness_S-oc-temp-lat, | 134 | aparc-a2009s_rh_thickness_S-oc-temp-lat, |
| 61 | aparc-a2009s_lh_thickness_S-oc-temp-med+Lingual, | 135 | aparc-a2009s_rh_thickness_S-oc-temp-med+Lingual, |
| 62 | aparc-a2009s_lh_thickness_S-orbital-lateral, | 136 | aparc-a2009s_rh_thickness_S-orbital-lateral, |
| 63 | aparc-a2009s_lh_thickness_S-orbital-med-olfact, | 137 | aparc-a2009s_rh_thickness_S-orbital-med-olfact, |
| 64 | aparc-a2009s_lh_thickness_S-orbital-H-Shaped, | 138 | aparc-a2009s_rh_thickness_S-orbital-H-Shaped, |
| 65 | aparc-a2009s_lh_thickness_S-parieto-occipital, | 139 | aparc-a2009s_rh_thickness_S-parieto-occipital, |
| 66 | aparc-a2009s_lh_thickness_S-pericallosal, | 140 | aparc-a2009s_rh_thickness_S-pericallosal, |
| 67 | aparc-a2009s_lh_thickness_S-postcentral, | 141 | aparc-a2009s_rh_thickness_S-postcentral, |
| 68 | aparc-a2009s_lh_thickness_S-precentral-inf-part, | 142 | aparc-a2009s_rh_thickness_S-precentral-inf-part, |
| 69 | aparc-a2009s_lh_thickness_S-precentral-sup-part, | 143 | aparc-a2009s_rh_thickness_S-precentral-sup-part, |
| 70 | aparc-a2009s_lh_thickness_S-suborbital, | 144 | aparc-a2009s_rh_thickness_S-suborbital, |
| 71 | aparc-a2009s_lh_thickness_S-subparietal, | 145 | aparc-a2009s_rh_thickness_S-subparietal, |
| 72 | aparc-a2009s_lh_thickness_S-temporal-inf, | 146 | aparc-a2009s_rh_thickness_S-temporal-inf, |
| 73 | aparc-a2009s_lh_thickness_S-temporal-sup, | 147 | aparc-a2009s_rh_thickness_S-temporal-sup, |
| 74 | aparc-a2009s_lh_thickness_S-temporal-transverse, | 148 | aparc-a2009s_rh_thickness_S-temporal-transverse, |

Table S 9 Names of UK Biobank Imaging Derived Phenotypes (IDPs) related to White Matter: 453. Supplement to **Figure 6**.

| IDP # | IDP Name | IDP # | IDP Name |
|---|---|---|---|
| 1 | IDP_T2_FLAIR_BIANCA_WMH_volume | 78 | IDP_dMRI_ProbtrackX_FA_unc_r |
| 2 | IDP_T2_FLAIR_BIANCA_periventWMH_volume | 79 | IDP_dMRI_TBSS_MO_Middle_cerebellar_peduncle |
| 3 | IDP_T2_FLAIR_BIANCA_deepWMH_volume | 80 | IDP_dMRI_TBSS_MO_Pontine_crossing_tract |
| 4 | IDP_dMRI_TBSS_FA_Middle_cerebellar_peduncle | 81 | IDP_dMRI_TBSS_MO_Genu_of_corpus_callosum |
| 5 | IDP_dMRI_TBSS_FA_Pontine_crossing_tract | 82 | IDP_dMRI_TBSS_MO_Body_of_corpus_callosum |
| 6 | IDP_dMRI_TBSS_FA_Genu_of_corpus_callosum | 83 | IDP_dMRI_TBSS_MO_Splenium_of_corpus_callosum |
| 7 | IDP_dMRI_TBSS_FA_Body_of_corpus_callosum | 84 | IDP_dMRI_TBSS_MO_Fornix |
| 8 | IDP_dMRI_TBSS_FA_Splenium_of_corpus_callosum | 85 | IDP_dMRI_TBSS_MO_Corticospinal_tract_R |
| 9 | IDP_dMRI_TBSS_FA_Fornix | 86 | IDP_dMRI_TBSS_MO_Corticospinal_tract_L |
| 10 | IDP_dMRI_TBSS_FA_Corticospinal_tract_R | 87 | IDP_dMRI_TBSS_MO_Medial_lemniscus_R |
| 11 | IDP_dMRI_TBSS_FA_Corticospinal_tract_L | 88 | IDP_dMRI_TBSS_MO_Medial_lemniscus_L |
| 12 | IDP_dMRI_TBSS_FA_Medial_lemniscus_R | 89 | IDP_dMRI_TBSS_MO_Inferior_cerebellar_peduncle_R |
| 13 | IDP_dMRI_TBSS_FA_Medial_lemniscus_L | 90 | IDP_dMRI_TBSS_MO_Inferior_cerebellar_peduncle_L |
| 14 | IDP_dMRI_TBSS_FA_Inferior_cerebellar_peduncle_R | 91 | IDP_dMRI_TBSS_MO_Superior_cerebellar_peduncle_R |
| 15 | IDP_dMRI_TBSS_FA_Inferior_cerebellar_peduncle_L | 92 | IDP_dMRI_TBSS_MO_Superior_cerebellar_peduncle_L |
| 16 | IDP_dMRI_TBSS_FA_Superior_cerebellar_peduncle_R | 93 | IDP_dMRI_TBSS_MO_Cerebral_peduncle_R |
| 17 | IDP_dMRI_TBSS_FA_Superior_cerebellar_peduncle_L | 94 | IDP_dMRI_TBSS_MO_Cerebral_peduncle_L |
| 18 | IDP_dMRI_TBSS_FA_Cerebral_peduncle_R | 95 | IDP_dMRI_TBSS_MO_Anterior_limb_of_internal_capsule_R |
| 19 | IDP_dMRI_TBSS_FA_Cerebral_peduncle_L | 96 | IDP_dMRI_TBSS_MO_Anterior_limb_of_internal_capsule_L |
| 20 | IDP_dMRI_TBSS_FA_Anterior_limb_of_internal_capsule_R | 97 | IDP_dMRI_TBSS_MO_Posterior_limb_of_internal_capsule_R |
| 21 | IDP_dMRI_TBSS_FA_Anterior_limb_of_internal_capsule_L | 98 | IDP_dMRI_TBSS_MO_Posterior_limb_of_internal_capsule_L |
| 22 | IDP_dMRI_TBSS_FA_Posterior_limb_of_internal_capsule_R | 99 | IDP_dMRI_TBSS_MO_Retrolenticular_part_of_internal_capsule_R |
| 23 | IDP_dMRI_TBSS_FA_Posterior_limb_of_internal_capsule_L | 100 | IDP_dMRI_TBSS_MO_Retrolenticular_part_of_internal_capsule_L |
| 24 | IDP_dMRI_TBSS_FA_Retrolenticular_part_of_internal_capsule_R | 101 | IDP_dMRI_TBSS_MO_Anterior_corona_radiata_R |
| 25 | IDP_dMRI_TBSS_FA_Retrolenticular_part_of_internal_capsule_L | 102 | IDP_dMRI_TBSS_MO_Anterior_corona_radiata_L |
| 26 | IDP_dMRI_TBSS_FA_Anterior_corona_radiata_R | 103 | IDP_dMRI_TBSS_MO_Superior_corona_radiata_R |
| 27 | IDP_dMRI_TBSS_FA_Anterior_corona_radiata_L | 104 | IDP_dMRI_TBSS_MO_Superior_corona_radiata_L |
| 28 | IDP_dMRI_TBSS_FA_Superior_corona_radiata_R | 105 | IDP_dMRI_TBSS_MO_Posterior_corona_radiata_R |
| 29 | IDP_dMRI_TBSS_FA_Superior_corona_radiata_L | 106 | IDP_dMRI_TBSS_MO_Posterior_corona_radiata_L |
| 30 | IDP_dMRI_TBSS_FA_Posterior_corona_radiata_R | 107 | IDP_dMRI_TBSS_MO_Posterior_thalamic_radiation_R |
| 31 | IDP_dMRI_TBSS_FA_Posterior_corona_radiata_L | 108 | IDP_dMRI_TBSS_MO_Posterior_thalamic_radiation_L |
| 32 | IDP_dMRI_TBSS_FA_Posterior_thalamic_radiation_R | 109 | IDP_dMRI_TBSS_MO_Sagittal_stratum_R |
| 33 | IDP_dMRI_TBSS_FA_Posterior_thalamic_radiation_L | 110 | IDP_dMRI_TBSS_MO_Sagittal_stratum_L |
| 34 | IDP_dMRI_TBSS_FA_Sagittal_stratum_R | 111 | IDP_dMRI_TBSS_MO_External_capsule_R |
| 35 | IDP_dMRI_TBSS_FA_Sagittal_stratum_L | 112 | IDP_dMRI_TBSS_MO_External_capsule_L |
| 36 | IDP_dMRI_TBSS_FA_External_capsule_R | 113 | IDP_dMRI_TBSS_MO_Cingulum_cingulate_gyrus_R |
| 37 | IDP_dMRI_TBSS_FA_External_capsule_L | 114 | IDP_dMRI_TBSS_MO_Cingulum_cingulate_gyrus_L |
| 38 | IDP_dMRI_TBSS_FA_Cingulum_cingulate_gyrus_R | 115 | IDP_dMRI_TBSS_MO_Cingulum_hippocampus_R |
| 39 | IDP_dMRI_TBSS_FA_Cingulum_cingulate_gyrus_L | 116 | IDP_dMRI_TBSS_MO_Cingulum_hippocampus_L |
| 40 | IDP_dMRI_TBSS_FA_Cingulum_hippocampus_R | 117 | IDP_dMRI_TBSS_MO_Fornix_cres+Stria_terminalis_R |
| 41 | IDP_dMRI_TBSS_FA_Cingulum_hippocampus_L | 118 | IDP_dMRI_TBSS_MO_Fornix_cres+Stria_terminalis_L |
| 42 | IDP_dMRI_TBSS_FA_Fornix_cres+Stria_terminalis_R | 119 | IDP_dMRI_TBSS_MO_Superior_longitudinal_fasciculus_R |
| 43 | IDP_dMRI_TBSS_FA_Fornix_cres+Stria_terminalis_L | 120 | IDP_dMRI_TBSS_MO_Superior_longitudinal_fasciculus_L |
| 44 | IDP_dMRI_TBSS_FA_Superior_longitudinal_fasciculus_R | 121 | IDP_dMRI_TBSS_MO_Superior_fronto-occipital_fasciculus_R |
| 45 | IDP_dMRI_TBSS_FA_Superior_longitudinal_fasciculus_L | 122 | IDP_dMRI_TBSS_MO_Superior_fronto-occipital_fasciculus_L |
| 46 | IDP_dMRI_TBSS_FA_Superior_fronto-occipital_fasciculus_R | 123 | IDP_dMRI_TBSS_MO_Uncinate_fasciculus_R |
| 47 | IDP_dMRI_TBSS_FA_Superior_fronto-occipital_fasciculus_L | 124 | IDP_dMRI_TBSS_MO_Uncinate_fasciculus_L |
| 48 | IDP_dMRI_TBSS_FA_Uncinate_fasciculus_R | 125 | IDP_dMRI_TBSS_MO_Tapetum_R |
| 49 | IDP_dMRI_TBSS_FA_Uncinate_fasciculus_L | 126 | IDP_dMRI_TBSS_MO_Tapetum_L |
| 50 | IDP_dMRI_TBSS_FA_Tapetum_R | 127 | IDP_dMRI_ProbtrackX_MO_ar_l |
| 51 | IDP_dMRI_TBSS_FA_Tapetum_L | 128 | IDP_dMRI_ProbtrackX_MO_ar_r |
| 52 | IDP_dMRI_ProbtrackX_FA_ar_l | 129 | IDP_dMRI_ProbtrackX_MO_atr_l |
| 53 | IDP_dMRI_ProbtrackX_FA_ar_r | 130 | IDP_dMRI_ProbtrackX_MO_atr_r |
| 54 | IDP_dMRI_ProbtrackX_FA_atr_l | 131 | IDP_dMRI_ProbtrackX_MO_cgc_l |
| 55 | IDP_dMRI_ProbtrackX_FA_atr_r | 132 | IDP_dMRI_ProbtrackX_MO_cgc_r |
| 56 | IDP_dMRI_ProbtrackX_FA_cgc_l | 133 | IDP_dMRI_ProbtrackX_MO_cgh_l |
| 57 | IDP_dMRI_ProbtrackX_FA_cgc_r | 134 | IDP_dMRI_ProbtrackX_MO_cgh_r |
| 58 | IDP_dMRI_ProbtrackX_FA_cgh_l | 135 | IDP_dMRI_ProbtrackX_MO_cst_l |
| 59 | IDP_dMRI_ProbtrackX_FA_cgh_r | 136 | IDP_dMRI_ProbtrackX_MO_cst_r |
| 60 | IDP_dMRI_ProbtrackX_FA_cst_l | 137 | IDP_dMRI_ProbtrackX_MO_fma |
| 61 | IDP_dMRI_ProbtrackX_FA_cst_r | 138 | IDP_dMRI_ProbtrackX_MO_fmi |
| 62 | IDP_dMRI_ProbtrackX_FA_fma | 139 | IDP_dMRI_ProbtrackX_MO_ifo_l |
| 63 | IDP_dMRI_ProbtrackX_FA_fmi | 140 | IDP_dMRI_ProbtrackX_MO_ifo_r |
| 64 | IDP_dMRI_ProbtrackX_FA_ifo_l | 141 | IDP_dMRI_ProbtrackX_MO_ilf_l |
| 65 | IDP_dMRI_ProbtrackX_FA_ifo_r | 142 | IDP_dMRI_ProbtrackX_MO_ilf_r |
| 66 | IDP_dMRI_ProbtrackX_FA_ilf_l | 143 | IDP_dMRI_ProbtrackX_MO_mcp |
| 67 | IDP_dMRI_ProbtrackX_FA_ilf_r | 144 | IDP_dMRI_ProbtrackX_MO_ml_l |
| 68 | IDP_dMRI_ProbtrackX_FA_mcp | 145 | IDP_dMRI_ProbtrackX_MO_ml_r |
| 69 | IDP_dMRI_ProbtrackX_FA_ml_l | 146 | IDP_dMRI_ProbtrackX_MO_ptr_l |
| 70 | IDP_dMRI_ProbtrackX_FA_ml_r | 147 | IDP_dMRI_ProbtrackX_MO_ptr_r |
| 71 | IDP_dMRI_ProbtrackX_FA_ptr_l | 148 | IDP_dMRI_ProbtrackX_MO_slf_l |
| 72 | IDP_dMRI_ProbtrackX_FA_ptr_r | 149 | IDP_dMRI_ProbtrackX_MO_slf_r |
| 73 | IDP_dMRI_ProbtrackX_FA_slf_l | 150 | IDP_dMRI_ProbtrackX_MO_str_l |
| 74 | IDP_dMRI_ProbtrackX_FA_slf_r | 151 | IDP_dMRI_ProbtrackX_MO_str_r |
| 75 | IDP_dMRI_ProbtrackX_FA_str_l | 152 | IDP_dMRI_ProbtrackX_MO_unc_l |
| 76 | IDP_dMRI_ProbtrackX_FA_str_r | 153 | IDP_dMRI_ProbtrackX_MO_unc_r |
| 77 | IDP_dMRI_ProbtrackX_FA_unc_l | 154 | IDP_dMRI_TBSS_MD_Middle_cerebellar_peduncle |

Table S 10 Names of UK Biobank Imaging Derived Phenotypes (IDPs) related to White Matter-continued: 453. Supplement to **Figure 6**.

| IDP # | IDP Name | IDP # | IDP Name |
|---|---|---|---|
| 155 | IDP_dMRI_TBSS_MD_Pontine_crossing_tract | 232 | IDP_dMRI_TBSS_ICVF_Body_of_corpus_callosum |
| 156 | IDP_dMRI_TBSS_MD_Genu_of_corpus_callosum | 233 | IDP_dMRI_TBSS_ICVF_Splenium_of_corpus_callosum |
| 157 | IDP_dMRI_TBSS_MD_Body_of_corpus_callosum | 234 | IDP_dMRI_TBSS_ICVF_Fornix |
| 158 | IDP_dMRI_TBSS_MD_Splenium_of_corpus_callosum | 235 | IDP_dMRI_TBSS_ICVF_Corticospinal_tract_R |
| 159 | IDP_dMRI_TBSS_MD_Fornix | 236 | IDP_dMRI_TBSS_ICVF_Corticospinal_tract_L |
| 160 | IDP_dMRI_TBSS_MD_Corticospinal_tract_R | 237 | IDP_dMRI_TBSS_ICVF_Medial_lemniscus_R |
| 161 | IDP_dMRI_TBSS_MD_Corticospinal_tract_L | 238 | IDP_dMRI_TBSS_ICVF_Medial_lemniscus_L |
| 162 | IDP_dMRI_TBSS_MD_Medial_lemniscus_R | 239 | IDP_dMRI_TBSS_ICVF_Inferior_cerebellar_peduncle_R |
| 163 | IDP_dMRI_TBSS_MD_Medial_lemniscus_L | 240 | IDP_dMRI_TBSS_ICVF_Inferior_cerebellar_peduncle_L |
| 164 | IDP_dMRI_TBSS_MD_Inferior_cerebellar_peduncle_R | 241 | IDP_dMRI_TBSS_ICVF_Superior_cerebellar_peduncle_R |
| 165 | IDP_dMRI_TBSS_MD_Inferior_cerebellar_peduncle_L | 242 | IDP_dMRI_TBSS_ICVF_Superior_cerebellar_peduncle_L |
| 166 | IDP_dMRI_TBSS_MD_Superior_cerebellar_peduncle_R | 243 | IDP_dMRI_TBSS_ICVF_Cerebral_peduncle_R |
| 167 | IDP_dMRI_TBSS_MD_Superior_cerebellar_peduncle_L | 244 | IDP_dMRI_TBSS_ICVF_Cerebral_peduncle_L |
| 168 | IDP_dMRI_TBSS_MD_Cerebral_peduncle_R | 245 | IDP_dMRI_TBSS_ICVF_Anterior_limb_of_internal_capsule_R |
| 169 | IDP_dMRI_TBSS_MD_Cerebral_peduncle_L | 246 | IDP_dMRI_TBSS_ICVF_Anterior_limb_of_internal_capsule_L |
| 170 | IDP_dMRI_TBSS_MD_Anterior_limb_of_internal_capsule_R | 247 | IDP_dMRI_TBSS_ICVF_Posterior_limb_of_internal_capsule_R |
| 171 | IDP_dMRI_TBSS_MD_Anterior_limb_of_internal_capsule_L | 248 | IDP_dMRI_TBSS_ICVF_Posterior_limb_of_internal_capsule_L |
| 172 | IDP_dMRI_TBSS_MD_Posterior_limb_of_internal_capsule_R | 249 | IDP_dMRI_TBSS_ICVF_Retrolenticular_part_of_internal_capsule_R |
| 173 | IDP_dMRI_TBSS_MD_Posterior_limb_of_internal_capsule_L | 250 | IDP_dMRI_TBSS_ICVF_Retrolenticular_part_of_internal_capsule_L |
| 174 | IDP_dMRI_TBSS_MD_Retrolenticular_part_of_internal_capsule_R | 251 | IDP_dMRI_TBSS_ICVF_Anterior_corona_radiata_R |
| 175 | IDP_dMRI_TBSS_MD_Retrolenticular_part_of_internal_capsule_L | 252 | IDP_dMRI_TBSS_ICVF_Anterior_corona_radiata_L |
| 176 | IDP_dMRI_TBSS_MD_Anterior_corona_radiata_R | 253 | IDP_dMRI_TBSS_ICVF_Superior_corona_radiata_R |
| 177 | IDP_dMRI_TBSS_MD_Anterior_corona_radiata_L | 254 | IDP_dMRI_TBSS_ICVF_Superior_corona_radiata_L |
| 178 | IDP_dMRI_TBSS_MD_Superior_corona_radiata_R | 255 | IDP_dMRI_TBSS_ICVF_Posterior_corona_radiata_R |
| 179 | IDP_dMRI_TBSS_MD_Superior_corona_radiata_L | 256 | IDP_dMRI_TBSS_ICVF_Posterior_corona_radiata_L |
| 180 | IDP_dMRI_TBSS_MD_Posterior_corona_radiata_R | 257 | IDP_dMRI_TBSS_ICVF_Posterior_thalamic_radiation_R |
| 181 | IDP_dMRI_TBSS_MD_Posterior_corona_radiata_L | 258 | IDP_dMRI_TBSS_ICVF_Posterior_thalamic_radiation_L |
| 182 | IDP_dMRI_TBSS_MD_Posterior_thalamic_radiation_R | 259 | IDP_dMRI_TBSS_ICVF_Sagittal_stratum_R |
| 183 | IDP_dMRI_TBSS_MD_Posterior_thalamic_radiation_L | 260 | IDP_dMRI_TBSS_ICVF_Sagittal_stratum_L |
| 184 | IDP_dMRI_TBSS_MD_Sagittal_stratum_R | 261 | IDP_dMRI_TBSS_ICVF_External_capsule_R |
| 185 | IDP_dMRI_TBSS_MD_Sagittal_stratum_L | 262 | IDP_dMRI_TBSS_ICVF_External_capsule_L |
| 186 | IDP_dMRI_TBSS_MD_External_capsule_R | 263 | IDP_dMRI_TBSS_ICVF_Cingulum_cingulate_gyrus_R |
| 187 | IDP_dMRI_TBSS_MD_External_capsule_L | 264 | IDP_dMRI_TBSS_ICVF_Cingulum_cingulate_gyrus_L |
| 188 | IDP_dMRI_TBSS_MD_Cingulum_cingulate_gyrus_R | 265 | IDP_dMRI_TBSS_ICVF_Cingulum_hippocampus_R |
| 189 | IDP_dMRI_TBSS_MD_Cingulum_cingulate_gyrus_L | 266 | IDP_dMRI_TBSS_ICVF_Cingulum_hippocampus_L |
| 190 | IDP_dMRI_TBSS_MD_Cingulum_hippocampus_R | 267 | IDP_dMRI_TBSS_ICVF_Fornix_cres+Stria_terminalis_R |
| 191 | IDP_dMRI_TBSS_MD_Cingulum_hippocampus_L | 268 | IDP_dMRI_TBSS_ICVF_Fornix_cres+Stria_terminalis_L |
| 192 | IDP_dMRI_TBSS_MD_Fornix_cres+Stria_terminalis_R | 269 | IDP_dMRI_TBSS_ICVF_Superior_longitudinal_fasciculus_R |
| 193 | IDP_dMRI_TBSS_MD_Fornix_cres+Stria_terminalis_L | 270 | IDP_dMRI_TBSS_ICVF_Superior_longitudinal_fasciculus_L |
| 194 | IDP_dMRI_TBSS_MD_Superior_longitudinal_fasciculus_R | 271 | IDP_dMRI_TBSS_ICVF_Superior_fronto-occipital_fasciculus_R |
| 195 | IDP_dMRI_TBSS_MD_Superior_longitudinal_fasciculus_L | 272 | IDP_dMRI_TBSS_ICVF_Superior_fronto-occipital_fasciculus_L |
| 196 | IDP_dMRI_TBSS_MD_Superior_fronto-occipital_fasciculus_R | 273 | IDP_dMRI_TBSS_ICVF_Uncinate_fasciculus_R |
| 197 | IDP_dMRI_TBSS_MD_Superior_fronto-occipital_fasciculus_L | 274 | IDP_dMRI_TBSS_ICVF_Uncinate_fasciculus_L |
| 198 | IDP_dMRI_TBSS_MD_Uncinate_fasciculus_R | 275 | IDP_dMRI_TBSS_ICVF_Tapetum_R |
| 199 | IDP_dMRI_TBSS_MD_Uncinate_fasciculus_L | 276 | IDP_dMRI_TBSS_ICVF_Tapetum_L |
| 200 | IDP_dMRI_TBSS_MD_Tapetum_R | 277 | IDP_dMRI_ProbtrackX_ICVF_ar_l |
| 201 | IDP_dMRI_TBSS_MD_Tapetum_L | 278 | IDP_dMRI_ProbtrackX_ICVF_ar_r |
| 202 | IDP_dMRI_ProbtrackX_MD_ar_l | 279 | IDP_dMRI_ProbtrackX_ICVF_atr_l |
| 203 | IDP_dMRI_ProbtrackX_MD_ar_r | 280 | IDP_dMRI_ProbtrackX_ICVF_atr_r |
| 204 | IDP_dMRI_ProbtrackX_MD_atr_l | 281 | IDP_dMRI_ProbtrackX_ICVF_cgc_l |
| 205 | IDP_dMRI_ProbtrackX_MD_atr_r | 282 | IDP_dMRI_ProbtrackX_ICVF_cgc_r |
| 206 | IDP_dMRI_ProbtrackX_MD_cgc_l | 283 | IDP_dMRI_ProbtrackX_ICVF_cgh_l |
| 207 | IDP_dMRI_ProbtrackX_MD_cgc_r | 284 | IDP_dMRI_ProbtrackX_ICVF_cgh_r |
| 208 | IDP_dMRI_ProbtrackX_MD_cgh_l | 285 | IDP_dMRI_ProbtrackX_ICVF_cst_l |
| 209 | IDP_dMRI_ProbtrackX_MD_cgh_r | 286 | IDP_dMRI_ProbtrackX_ICVF_cst_r |
| 210 | IDP_dMRI_ProbtrackX_MD_cst_l | 287 | IDP_dMRI_ProbtrackX_ICVF_fma |
| 211 | IDP_dMRI_ProbtrackX_MD_cst_r | 288 | IDP_dMRI_ProbtrackX_ICVF_fmi |
| 212 | IDP_dMRI_ProbtrackX_MD_fma | 289 | IDP_dMRI_ProbtrackX_ICVF_ifo_l |
| 213 | IDP_dMRI_ProbtrackX_MD_fmi | 290 | IDP_dMRI_ProbtrackX_ICVF_ifo_r |
| 214 | IDP_dMRI_ProbtrackX_MD_ifo_l | 291 | IDP_dMRI_ProbtrackX_ICVF_ilf_l |
| 215 | IDP_dMRI_ProbtrackX_MD_ifo_r | 292 | IDP_dMRI_ProbtrackX_ICVF_ilf_r |
| 216 | IDP_dMRI_ProbtrackX_MD_ilf_l | 293 | IDP_dMRI_ProbtrackX_ICVF_mcp |
| 217 | IDP_dMRI_ProbtrackX_MD_ilf_r | 294 | IDP_dMRI_ProbtrackX_ICVF_ml_l |
| 218 | IDP_dMRI_ProbtrackX_MD_mcp | 295 | IDP_dMRI_ProbtrackX_ICVF_ml_r |
| 219 | IDP_dMRI_ProbtrackX_MD_ml_l | 296 | IDP_dMRI_ProbtrackX_ICVF_ptr_l |
| 220 | IDP_dMRI_ProbtrackX_MD_ml_r | 297 | IDP_dMRI_ProbtrackX_ICVF_ptr_r |
| 221 | IDP_dMRI_ProbtrackX_MD_ptr_l | 298 | IDP_dMRI_ProbtrackX_ICVF_slf_l |
| 222 | IDP_dMRI_ProbtrackX_MD_ptr_r | 299 | IDP_dMRI_ProbtrackX_ICVF_slf_r |
| 223 | IDP_dMRI_ProbtrackX_MD_slf_l | 300 | IDP_dMRI_ProbtrackX_ICVF_str_l |
| 224 | IDP_dMRI_ProbtrackX_MD_slf_r | 301 | IDP_dMRI_ProbtrackX_ICVF_str_r |
| 225 | IDP_dMRI_ProbtrackX_MD_str_l | 302 | IDP_dMRI_ProbtrackX_ICVF_unc_l |
| 226 | IDP_dMRI_ProbtrackX_MD_str_r | 303 | IDP_dMRI_ProbtrackX_ICVF_unc_r |
| 227 | IDP_dMRI_ProbtrackX_MD_unc_l | 304 | IDP_dMRI_TBSS_OD_Middle_cerebellar_peduncle |
| 228 | IDP_dMRI_ProbtrackX_MD_unc_r | 305 | IDP_dMRI_TBSS_OD_Pontine_crossing_tract |
| 229 | IDP_dMRI_TBSS_ICVF_Middle_cerebellar_peduncle | 306 | IDP_dMRI_TBSS_OD_Genu_of_corpus_callosum |
| 230 | IDP_dMRI_TBSS_ICVF_Pontine_crossing_tract | 307 | IDP_dMRI_TBSS_OD_Body_of_corpus_callosum |
| 231 | IDP_dMRI_TBSS_ICVF_Genu_of_corpus_callosum | 308 | IDP_dMRI_TBSS_OD_Splenium_of_corpus_callosum |

Table S 11 Names of UK Biobank Imaging Derived Phenotypes (IDPs) related to White Matter-continued: 453. Supplement to **Figure 6**.

| IDP # | IDP Name | IDP # | IDP Name |
|---|---|---|---|
| **309** | IDP_dMRI_TBSS_OD_Fornix | **386** | IDP_dMRI_TBSS_ISOVF_Corticospinal_tract_L |
| **310** | IDP_dMRI_TBSS_OD_Corticospinal_tract_R | **387** | IDP_dMRI_TBSS_ISOVF_Medial_lemniscus_R |
| **311** | IDP_dMRI_TBSS_OD_Corticospinal_tract_L | **388** | IDP_dMRI_TBSS_ISOVF_Medial_lemniscus_L |
| **312** | IDP_dMRI_TBSS_OD_Medial_lemniscus_R | **389** | IDP_dMRI_TBSS_ISOVF_Inferior_cerebellar_peduncle_R |
| **313** | IDP_dMRI_TBSS_OD_Medial_lemniscus_L | **390** | IDP_dMRI_TBSS_ISOVF_Inferior_cerebellar_peduncle_L |
| **314** | IDP_dMRI_TBSS_OD_Inferior_cerebellar_peduncle_R | **391** | IDP_dMRI_TBSS_ISOVF_Superior_cerebellar_peduncle_R |
| **315** | IDP_dMRI_TBSS_OD_Inferior_cerebellar_peduncle_L | **392** | IDP_dMRI_TBSS_ISOVF_Superior_cerebellar_peduncle_L |
| **316** | IDP_dMRI_TBSS_OD_Superior_cerebellar_peduncle_R | **393** | IDP_dMRI_TBSS_ISOVF_Cerebral_peduncle_R |
| **317** | IDP_dMRI_TBSS_OD_Superior_cerebellar_peduncle_L | **394** | IDP_dMRI_TBSS_ISOVF_Cerebral_peduncle_L |
| **318** | IDP_dMRI_TBSS_OD_Cerebral_peduncle_R | **395** | IDP_dMRI_TBSS_ISOVF_Anterior_limb_of_internal_capsule_R |
| **319** | IDP_dMRI_TBSS_OD_Cerebral_peduncle_L | **396** | IDP_dMRI_TBSS_ISOVF_Anterior_limb_of_internal_capsule_L |
| **320** | IDP_dMRI_TBSS_OD_Anterior_limb_of_internal_capsule_R | **397** | IDP_dMRI_TBSS_ISOVF_Posterior_limb_of_internal_capsule_R |
| **321** | IDP_dMRI_TBSS_OD_Anterior_limb_of_internal_capsule_L | **398** | IDP_dMRI_TBSS_ISOVF_Posterior_limb_of_internal_capsule_L |
| **322** | IDP_dMRI_TBSS_OD_Posterior_limb_of_internal_capsule_R | **399** | IDP_dMRI_TBSS_ISOVF_Retrolenticular_part_of_internal_capsule_R |
| **323** | IDP_dMRI_TBSS_OD_Posterior_limb_of_internal_capsule_L | **400** | IDP_dMRI_TBSS_ISOVF_Retrolenticular_part_of_internal_capsule_L |
| **324** | IDP_dMRI_TBSS_OD_Retrolenticular_part_of_internal_capsule_R | **401** | IDP_dMRI_TBSS_ISOVF_Anterior_corona_radiata_R |
| **325** | IDP_dMRI_TBSS_OD_Retrolenticular_part_of_internal_capsule_L | **402** | IDP_dMRI_TBSS_ISOVF_Anterior_corona_radiata_L |
| **326** | IDP_dMRI_TBSS_OD_Anterior_corona_radiata_R | **403** | IDP_dMRI_TBSS_ISOVF_Superior_corona_radiata_R |
| **327** | IDP_dMRI_TBSS_OD_Anterior_corona_radiata_L | **404** | IDP_dMRI_TBSS_ISOVF_Superior_corona_radiata_L |
| **328** | IDP_dMRI_TBSS_OD_Superior_corona_radiata_R | **405** | IDP_dMRI_TBSS_ISOVF_Posterior_corona_radiata_R |
| **329** | IDP_dMRI_TBSS_OD_Superior_corona_radiata_L | **406** | IDP_dMRI_TBSS_ISOVF_Posterior_corona_radiata_L |
| **330** | IDP_dMRI_TBSS_OD_Posterior_corona_radiata_R | **407** | IDP_dMRI_TBSS_ISOVF_Posterior_thalamic_radiation_R |
| **331** | IDP_dMRI_TBSS_OD_Posterior_corona_radiata_L | **408** | IDP_dMRI_TBSS_ISOVF_Posterior_thalamic_radiation_L |
| **332** | IDP_dMRI_TBSS_OD_Posterior_thalamic_radiation_R | **409** | IDP_dMRI_TBSS_ISOVF_Sagittal_stratum_R |
| **333** | IDP_dMRI_TBSS_OD_Posterior_thalamic_radiation_L | **410** | IDP_dMRI_TBSS_ISOVF_Sagittal_stratum_L |
| **334** | IDP_dMRI_TBSS_OD_Sagittal_stratum_R | **411** | IDP_dMRI_TBSS_ISOVF_External_capsule_R |
| **335** | IDP_dMRI_TBSS_OD_Sagittal_stratum_L | **412** | IDP_dMRI_TBSS_ISOVF_External_capsule_L |
| **336** | IDP_dMRI_TBSS_OD_External_capsule_R | **413** | IDP_dMRI_TBSS_ISOVF_Cingulum_cingulate_gyrus_R |
| **337** | IDP_dMRI_TBSS_OD_External_capsule_L | **414** | IDP_dMRI_TBSS_ISOVF_Cingulum_cingulate_gyrus_L |
| **338** | IDP_dMRI_TBSS_OD_Cingulum_cingulate_gyrus_R | **415** | IDP_dMRI_TBSS_ISOVF_Cingulum_hippocampus_R |
| **339** | IDP_dMRI_TBSS_OD_Cingulum_cingulate_gyrus_L | **416** | IDP_dMRI_TBSS_ISOVF_Cingulum_hippocampus_L |
| **340** | IDP_dMRI_TBSS_OD_Cingulum_hippocampus_R | **417** | IDP_dMRI_TBSS_ISOVF_Fornix_cres+Stria_terminalis_R |
| **341** | IDP_dMRI_TBSS_OD_Cingulum_hippocampus_L | **418** | IDP_dMRI_TBSS_ISOVF_Fornix_cres+Stria_terminalis_L |
| **342** | IDP_dMRI_TBSS_OD_Fornix_cres+Stria_terminalis_R | **419** | IDP_dMRI_TBSS_ISOVF_Superior_longitudinal_fasciculus_R |
| **343** | IDP_dMRI_TBSS_OD_Fornix_cres+Stria_terminalis_L | **420** | IDP_dMRI_TBSS_ISOVF_Superior_longitudinal_fasciculus_L |
| **344** | IDP_dMRI_TBSS_OD_Superior_longitudinal_fasciculus_R | **421** | IDP_dMRI_TBSS_ISOVF_Superior_fronto-occipital_fasciculus_R |
| **345** | IDP_dMRI_TBSS_OD_Superior_longitudinal_fasciculus_L | **422** | IDP_dMRI_TBSS_ISOVF_Superior_fronto-occipital_fasciculus_L |
| **346** | IDP_dMRI_TBSS_OD_Superior_fronto-occipital_fasciculus_R | **423** | IDP_dMRI_TBSS_ISOVF_Uncinate_fasciculus_R |
| **347** | IDP_dMRI_TBSS_OD_Superior_fronto-occipital_fasciculus_L | **424** | IDP_dMRI_TBSS_ISOVF_Uncinate_fasciculus_L |
| **348** | IDP_dMRI_TBSS_OD_Uncinate_fasciculus_R | **425** | IDP_dMRI_TBSS_ISOVF_Tapetum_R |
| **349** | IDP_dMRI_TBSS_OD_Uncinate_fasciculus_L | **426** | IDP_dMRI_TBSS_ISOVF_Tapetum_L |
| **350** | IDP_dMRI_TBSS_OD_Tapetum_R | **427** | IDP_dMRI_ProbtrackX_ISOVF_ar_l |
| **351** | IDP_dMRI_TBSS_OD_Tapetum_L | **428** | IDP_dMRI_ProbtrackX_ISOVF_ar_r |
| **352** | IDP_dMRI_ProbtrackX_OD_ar_l | **429** | IDP_dMRI_ProbtrackX_ISOVF_atr_l |
| **353** | IDP_dMRI_ProbtrackX_OD_ar_r | **430** | IDP_dMRI_ProbtrackX_ISOVF_atr_r |
| **354** | IDP_dMRI_ProbtrackX_OD_atr_l | **431** | IDP_dMRI_ProbtrackX_ISOVF_cgc_l |
| **355** | IDP_dMRI_ProbtrackX_OD_atr_r | **432** | IDP_dMRI_ProbtrackX_ISOVF_cgc_r |
| **356** | IDP_dMRI_ProbtrackX_OD_cgc_l | **433** | IDP_dMRI_ProbtrackX_ISOVF_cgh_l |
| **357** | IDP_dMRI_ProbtrackX_OD_cgc_r | **434** | IDP_dMRI_ProbtrackX_ISOVF_cgh_r |
| **358** | IDP_dMRI_ProbtrackX_OD_cgh_l | **435** | IDP_dMRI_ProbtrackX_ISOVF_cst_l |
| **359** | IDP_dMRI_ProbtrackX_OD_cgh_r | **436** | IDP_dMRI_ProbtrackX_ISOVF_cst_r |
| **360** | IDP_dMRI_ProbtrackX_OD_cst_l | **437** | IDP_dMRI_ProbtrackX_ISOVF_fma |
| **361** | IDP_dMRI_ProbtrackX_OD_cst_r | **438** | IDP_dMRI_ProbtrackX_ISOVF_fmi |
| **362** | IDP_dMRI_ProbtrackX_OD_fma | **439** | IDP_dMRI_ProbtrackX_ISOVF_ifo_l |
| **363** | IDP_dMRI_ProbtrackX_OD_fmi | **440** | IDP_dMRI_ProbtrackX_ISOVF_ifo_r |
| **364** | IDP_dMRI_ProbtrackX_OD_ifo_l | **441** | IDP_dMRI_ProbtrackX_ISOVF_ilf_l |
| **365** | IDP_dMRI_ProbtrackX_OD_ifo_r | **442** | IDP_dMRI_ProbtrackX_ISOVF_ilf_r |
| **366** | IDP_dMRI_ProbtrackX_OD_ilf_l | **443** | IDP_dMRI_ProbtrackX_ISOVF_mcp |
| **367** | IDP_dMRI_ProbtrackX_OD_ilf_r | **444** | IDP_dMRI_ProbtrackX_ISOVF_ml_l |
| **368** | IDP_dMRI_ProbtrackX_OD_mcp | **445** | IDP_dMRI_ProbtrackX_ISOVF_ml_r |
| **369** | IDP_dMRI_ProbtrackX_OD_ml_l | **446** | IDP_dMRI_ProbtrackX_ISOVF_ptr_l |
| **370** | IDP_dMRI_ProbtrackX_OD_ml_r | **447** | IDP_dMRI_ProbtrackX_ISOVF_ptr_r |
| **371** | IDP_dMRI_ProbtrackX_OD_ptr_l | **448** | IDP_dMRI_ProbtrackX_ISOVF_slf_l |
| **372** | IDP_dMRI_ProbtrackX_OD_ptr_r | **449** | IDP_dMRI_ProbtrackX_ISOVF_slf_r |
| **373** | IDP_dMRI_ProbtrackX_OD_slf_l | **450** | IDP_dMRI_ProbtrackX_ISOVF_str_l |
| **374** | IDP_dMRI_ProbtrackX_OD_slf_r | **451** | IDP_dMRI_ProbtrackX_ISOVF_str_r |
| **375** | IDP_dMRI_ProbtrackX_OD_str_l | **452** | IDP_dMRI_ProbtrackX_ISOVF_unc_l |
| **376** | IDP_dMRI_ProbtrackX_OD_str_r | **453** | IDP_dMRI_ProbtrackX_ISOVF_unc_r |
| **377** | IDP_dMRI_ProbtrackX_OD_unc_l | | |
| **378** | IDP_dMRI_ProbtrackX_OD_unc_r | | |
| **379** | IDP_dMRI_TBSS_ISOVF_Middle_cerebellar_peduncle | | |
| **380** | IDP_dMRI_TBSS_ISOVF_Pontine_crossing_tract | | |
| **381** | IDP_dMRI_TBSS_ISOVF_Genu_of_corpus_callosum | | |
| **382** | IDP_dMRI_TBSS_ISOVF_Body_of_corpus_callosum | | |
| **383** | IDP_dMRI_TBSS_ISOVF_Splenium_of_corpus_callosum | | |
| **384** | IDP_dMRI_TBSS_ISOVF_Fornix | | |
| **385** | IDP_dMRI_TBSS_ISOVF_Corticospinal_tract_R | | |
| | | | |

Table S 12 Names of UK Biobank Non-Imaging Derived Phenotypes (nIDPs) related to Cardiovascular Health: 77. Supplement to **Figure 6**.

| nIDP # | nIDP Name | nIDP # | nIDP Name |
|---|---|---|---|
| **1** | Pulse rate, automated reading (0.0), | **39** | Stroke volume during PWA (2.1), |
| **2** | Pulse rate, automated reading (0.1), | **40** | Mean arterial pressure during PWA (2.0), |
| **3** | Pulse rate, automated reading (2.0), | **41** | Mean arterial pressure during PWA (2.1), |
| **4** | Pulse rate, automated reading (2.1), | **42** | Ventricular rate (2.0), |
| **5** | Diastolic blood pressure, automated reading (0.0), | **43** | P duration (2.0), |
| **6** | Diastolic blood pressure, automated reading (0.1), | **44** | QRS duration (2.0), |
| **7** | Diastolic blood pressure, automated reading (2.0), | **45** | Systolic brachial blood pressure (2.0), |
| **8** | Diastolic blood pressure, automated reading (2.1), | **46** | Diastolic brachial blood pressure (2.0), |
| **9** | Systolic blood pressure, automated reading (0.0), | **47** | Cardiac index during PWA (2.0), |
| **10** | Systolic blood pressure, automated reading (0.1), | **48** | Cardiac index during PWA (2.1), |
| **11** | Systolic blood pressure, automated reading (2.0), | **49** | Pulse wave Arterial Stiffness index (0.0), |
| **12** | Systolic blood pressure, automated reading (2.1), | **50** | Pulse wave Arterial Stiffness index (2.0), |
| **13** | Pulse rate (0.0), | **51** | PQ interval (2.0), |
| **14** | Pulse rate (2.0), | **52** | QT interval (2.0), |
| **15** | Pulse wave reflection index (0.0), | **53** | QTC interval (2.0), |
| **16** | Pulse wave reflection index (2.0), | **54** | RR interval (2.0), |
| **17** | Pulse wave peak to peak time (0.0), | **55** | PP interval (2.0), |
| **18** | Pulse wave peak to peak time (2.0), | **56** | P axis (2.0), |
| **19** | Heart rate during PWA (2.0), | **57** | R axis (2.0), |
| **20** | Heart rate during PWA (2.1), | **58** | T axis (2.0), |
| **21** | Peripheral pulse pressure during PWA (2.0), | **59** | QRS num (2.0), |
| **22** | Central systolic blood pressure during PWA (2.0), | **60** | LV ejection fraction (2.0), |
| **23** | Central pulse pressure during PWA (2.0), | **61** | LV end diastolic volume (2.0), |
| **24** | Number of beats in waveform average for PWA (2.0), | **62** | LV end systolic volume (2.0), |
| **25** | Number of beats in waveform average for PWA (2.1), | **63** | LV stroke volume (2.0), |
| **26** | Central augmentation pressure during PWA (2.0), | **64** | Cardiac output (2.0), |
| **27** | Central augmentation pressure during PWA (2.1), | **65** | Average heart rate (2.0), |
| **28** | Augmentation index for PWA (2.0), | **66** | Minimum carotid IMT (intima-medial thickness) at 120 degrees (2.0), |
| **29** | Augmentation index for PWA (2.1), | **67** | Mean carotid IMT (intima-medial thickness) at 120 degrees (2.0), |
| **30** | Cardiac output during PWA (2.0), | **68** | Maximum carotid IMT (intima-medial thickness) at 120 degrees (2.0), |
| **31** | Cardiac output during PWA (2.1), | **69** | Minimum carotid IMT (intima-medial thickness) at 150 degrees (2.0), |
| **32** | End systolic pressure during PWA (2.0), | **70** | Mean carotid IMT (intima-medial thickness) at 150 degrees (2.0), |
| **33** | End systolic pressure during PWA (2.1), | **71** | Maximum carotid IMT (intima-medial thickness) at 150 degrees (2.0), |
| **34** | End systolic pressure index during PWA (2.0), | **72** | Minimum carotid IMT (intima-medial thickness) at 210 degrees (2.0), |
| **35** | End systolic pressure index during PWA (2.1), | **73** | Mean carotid IMT (intima-medial thickness) at 210 degrees (2.0), |
| **36** | Total peripheral resistance during PWA (2.0), | **74** | Maximum carotid IMT (intima-medial thickness) at 210 degrees (2.0), |
| **37** | Total peripheral resistance during PWA (2.1), | **75** | Minimum carotid IMT (intima-medial thickness) at 240 degrees (2.0), |
| **38** | Stroke volume during PWA (2.0), | **76** | Mean carotid IMT (intima-medial thickness) at 240 degrees (2.0), |
| | | **77** | Maximum carotid IMT (intima-medial thickness) at 240 degrees (2.0) |

Table S 13 Names of UK Biobank Non-Imaging Derived Phenotypes (nIDPs) related to Cognition: 68. Supplement to **Figure 6**.

| nIDP # | nIDP Name |
|---|---|
| 1 | Number of incorrect matches in round (0.1) |
| 2 | Number of incorrect matches in round (0.2) |
| 3 | Number of incorrect matches in round (2.1) |
| 4 | Number of incorrect matches in round (2.2) |
| 5 | Number of incorrect matches in round (2.3) |
| 6 | Number of times snap-button pressed (0.0) |
| 7 | Number of times snap-button pressed (0.1) |
| 8 | Number of times snap-button pressed (0.2) |
| 9 | Number of times snap-button pressed (0.3) |
| 10 | Number of times snap-button pressed (0.4) |
| 11 | Number of times snap-button pressed (0.10) |
| 12 | Number of times snap-button pressed (0.11) |
| 13 | Number of times snap-button pressed (2.0) |
| 14 | Number of times snap-button pressed (2.1) |
| 15 | Number of times snap-button pressed (2.2) |
| 16 | Number of times snap-button pressed (2.3) |
| 17 | Number of times snap-button pressed (2.4) |
| 18 | Number of times snap-button pressed (2.5) |
| 19 | Number of times snap-button pressed (2.7) |
| 20 | Number of times snap-button pressed (2.10) |
| 21 | Number of times snap-button pressed (2.11) |
| 22 | Prospective memory result (0.0) |
| 23 | Prospective memory result (2.0) |
| 24 | FI8 : chained arithmetic (2.0) |
| 25 | FI9 : concept interpolation (2.0) |
| 26 | Duration to complete numeric path (trail #1) (2.0) |
| 27 | Mean time to correctly identify matches (2.0) |
| 28 | Number of incorrect matches in round (0.0) |
| 29 | Time elapsed (2.0) |
| 30 | Time elapsed (2.1) |
| 31 | Time elapsed (2.2) |
| 32 | Time elapsed (2.3) |
| 33 | Time elapsed (2.4) |
| 34 | Time elapsed (2.5) |
| 35 | Time elapsed (2.6) |
| 36 | Time elapsed (2.7) |
| 37 | Time elapsed (2.8) |
| 38 | Digits entered correctly (2.0) |
| 39 | Digits entered correctly (2.1) |
| 40 | Digits entered correctly (2.2) |
| 41 | Digits entered correctly (2.3) |
| 42 | Digits entered correctly (2.4) |
| 43 | Digits entered correctly (2.5) |
| 44 | Digits entered correctly (2.6) |
| 45 | Digits entered correctly (2.7) |
| 46 | Total errors traversing numeric path (trail #1) (2.0) |
| 47 | Mean time to correctly identify matches (0.0) |
| 48 | Fluid intelligence score (0.0) |
| 49 | Fluid intelligence score (2.0) |
| 50 | Duration to complete numeric path (trail #1) (0.0) |
| 51 | Duration to complete alphanumeric path (trail #2) (0.0) |
| 52 | Digits entered correctly (2.8) |
| 53 | Maximum digits remembered correctly (2.0) |
| 54 | Attempted fluid intelligence (FI) test. (2.0) |
| 55 | FI1 : numeric addition test (0.0) |
| 56 | FI1 : numeric addition test (2.0) |
| 57 | FI3 : word interpolation (0.0) |
| 58 | FI3 : word interpolation (2.0) |
| 59 | FI4 : positional arithmetic (0.0) |
| 60 | FI4 : positional arithmetic (2.0) |
| 61 | FI5 : family relationship calculation (0.0) |
| 62 | FI5 : family relationship calculation (2.0) |
| 63 | FI6 : conditional arithmetic (0.0) |
| 64 | FI6 : conditional arithmetic (2.0) |
| 65 | FI7 : synonym (0.0) |
| 66 | FI7 : synonym (2.0) |
| 67 | Duration to complete alphanumeric path (trail #2) (2.0) |
| 68 | Number of puzzles correctly solved (2.0) |

Table S 14 Names of Human Connectome Project Non-Imaging Derived Phenotypes (nIDPs) used in CCA. Supplement to **Figure 6**.

| nIDP # | nIDP Name | nIDP # | nIDP Name |
|---|---|---|---|
| 1 | PicVocab_Unadj | 80 | ASR_Witd_Pct |
| 2 | PicVocab_AgeAdj | 81 | IWRD_TOT |
| 3 | PMAT24_A_CR | 82 | PainInterf_Tscore |
| 4 | DDisc_AUC_200 | 83 | MMSE_Score |
| 5 | THC | 84 | SSAGA_Alc_12_Frq_Drk |
| 6 | LifeSatisf_Unadj | 85 | Odor_Unadj |
| 7 | ListSort_AgeAdj | 86 | SSAGA_Alc_D4_Ab_Sx |
| 8 | ReadEng_Unadj | 87 | SSAGA_Mj_Use |
| 9 | SCPT_SPEC | 88 | ASR_Aggr_Raw |
| 10 | ReadEng_AgeAdj | 89 | SSAGA_Mj_Ab_Dep |
| 11 | ListSort_Unadj | 90 | DSM_Somp_Raw |
| 12 | DDisc_AUC_40K | 91 | FearSomat_Unadj |
| 13 | Avg_Weekday_Any_Tobacco_7days | 92 | SSAGA_Alc_12_Drinks_Per_Day |
| 14 | Num_Days_Used_Any_Tobacco_7days | 93 | Mars_Log_Score |
| 15 | Total_Any_Tobacco_7days | 94 | SelfEff_Unadj |
| 16 | PicSeq_AgeAdj | 95 | SCPT_SEN |
| 17 | FamHist_Fath_DrgAlc | 96 | NEOFAC_N |
| 18 | PicSeq_Unadj | 97 | SSAGA_Agoraphobia |
| 19 | Avg_Weekday_Cigarettes_7days | 98 | ASR_Intn_T |
| 20 | Avg_Weekend_Any_Tobacco_7days | 99 | AngHostil_Unadj |
| 21 | Total_Cigarettes_7days | 100 | Num_Days_Drank_7days |
| 22 | Dexterity_AgeAdj | 101 | SSAGA_Times_Used_Cocaine |
| 23 | Avg_Weekend_Cigarettes_7days | 102 | Loneliness_Unadj |
| 24 | Dexterity_Unadj | 103 | ASR_Intn_Raw |
| 25 | Times_Used_Any_Tobacco_Today | 104 | SSAGA_Alc_Hvy_Drinks_Per_Day |
| 26 | PSQI_Score | 105 | MeanPurp_Unadj |
| 27 | AngAggr_Unadj | 106 | DSM_Avoid_Pct |
| 28 | Taste_AgeAdj | 107 | NEOFAC_E |
| 29 | ASR_Rule_Raw | 108 | Total_Beer_Wine_Cooler_7days |
| 30 | Taste_Unadj | 109 | DSM_Avoid_Raw |
| 31 | ASR_Thot_Raw | 110 | Avg_Weekday_Wine_7days |
| 32 | EVA_Denom | 111 | Flanker_AgeAdj |
| 33 | SSAGA_TB_Still_Smoking | 112 | ASR_Anxd_Pct |
| 34 | FamHist_Fath_None | 113 | Avg_Weekend_Beer_Wine_Cooler_7days |
| 35 | ASR_Thot_Pct | 114 | SSAGA_Alc_D4_Ab_Dx |
| 36 | PercStress_Unadj | 115 | Total_Drinks_7days |
| 37 | ProcSpeed_AgeAdj | 116 | SSAGA_Alc_Hvy_Max_Drinks |
| 38 | ASR_Rule_Pct | 117 | FearAffect_Unadj |
| 39 | ProcSpeed_Unadj | 118 | Total_Wine_7days |
| 40 | DSM_Antis_Raw | 119 | Avg_Weekday_Drinks_7days |
| 41 | ER40_CR | 120 | ER40SAD |
| 42 | NEOFAC_A | 121 | Flanker_Unadj |
| 43 | ASR_Crit_Raw | 122 | ER40FEAR |
| 44 | VSPLOT_TC | 123 | Avg_Weekday_Beer_Wine_Cooler_7days |
| 45 | NEOFAC_O | 124 | SSAGA_Times_Used_Illicits |
| 46 | ER40ANG | 125 | Avg_Weekend_Drinks_7days |
| 47 | VSPLOT_OFF | 126 | SSAGA_Alc_D4_Dp_Sx |
| 48 | SSAGA_Times_Used_Stimulants | 127 | NEOFAC_C |
| 49 | ASR_Soma_Pct | 128 | Total_Hard_Liquor_7days |
| 50 | SSAGA_Mj_Times_Used | 129 | Correction |
| 51 | DSM_Antis_Pct | 130 | SSAGA_Alc_Hvy_Frq_5plus |
| 52 | CardSort_AgeAdj | 131 | DSM_Adh_Pct |
| 53 | ASR_Extn_Raw | 132 | ASR_Attn_Pct |
| 54 | ASR_Oth_Raw | 133 | VSPLOT_CRTE |
| 55 | ASR_Totp_T | 134 | SSAGA_Depressive_Ep |
| 56 | ASR_Extn_T | 135 | AngAffect_Unadj |
| 57 | ASR_Totp_Raw | 136 | SSAGA_PanicDisorder |
| 58 | EmotSupp_Unadj | 137 | Avg_Weekend_Hard_Liquor_7days |
| 59 | DSM_Anxi_Pct | 138 | FamHist_Moth_Dep |
| 60 | PercReject_Unadj | 139 | ASR_Anxd_Raw |
| 61 | ER40NOE | 140 | SSAGA_Times_Used_Opiates |
| 62 | DSM_Anxi_Raw | 141 | SSAGA_Times_Used_Sedatives |
| 63 | ASR_TAO_Sum | 142 | SSAGA_Alc_Hvy_Frq |
| 64 | SSAGA_TB_Smoking_History | 143 | SSAGA_Alc_12_Frq_5plus |
| 65 | CardSort_Unadj | 144 | Friendship_Unadj |
| 66 | PosAffect_Unadj | 145 | SSAGA_Depressive_Sx |
| 67 | SSAGA_ChildhoodConduct | 146 | ASR_Attn_Raw |
| 68 | Odor_AgeAdj | 147 | ASR_Intr_Raw |
| 69 | ASR_Witd_Raw | 148 | SSAGA_Alc_12_Frq |
| 70 | SSAGA_Alc_Hvy_Frq_Drk | 149 | FamHist_Fath_Dep |
| 71 | ASR_Soma_Raw | 150 | InstruSupp_Unadj |
| 72 | DSM_Depr_Pct | 151 | ASR_Intr_Pct |
| 73 | ASR_Aggr_Pct | 152 | SSAGA_Times_Used_Hallucinogens |
| 74 | SSAGA_Alc_12_Max_Drinks | 153 | Avg_Weekend_Wine_7days |
| 75 | DSM_Depr_Raw | 154 | FamHist_Moth_None |
| 76 | Mars_Final | 155 | Sadness_Unadj |
| 77 | PercHostil_Unadj | 156 | DSM_Hype_Raw |
| 78 | DSM_Somp_Pct | 157 | DSM_Adh_Raw |
| 79 | SSAGA_Alc_Age_1st_Use | 158 | DSM_Inat_Raw |
